# Bioactive Pyridone Alkaloids from a Deep-Sea-Derived Fungus *Arthrinium* sp. UJNMF0008

**DOI:** 10.3390/md16050174

**Published:** 2018-05-22

**Authors:** Jie Bao, Huijuan Zhai, Kongkai Zhu, Jin-Hai Yu, Yuying Zhang, Yinyin Wang, Cheng-Shi Jiang, Xiaoyong Zhang, Yun Zhang, Hua Zhang

**Affiliations:** 1 School of Biological Science and Technology, University of Jinan, 336 West Road of Nan Xinzhuang, Jinan 250022, China; bio_baoj@ujn.edu.cn (J.B.); zhai18363005528@163.com (H.Z.); hkhhh.k@163.com (K.Z.); yujinhai12@sina.com (J.-H.Y.); yuyingzhang2008@163.com (Y.Z.); bio_wangyy@ujn.edu.cn (Y.W.); jiangchengshi-20@163.com (C.-S.J.); 2 College of Marine Sciences, South China Agricultural University, 483 Wushan Road, Guangzhou 510642, China; zhangxiaoyong@scau.edu.cn; 3 Key Laboratory of Tropical Marine Bio-Resources and Ecology, South China Sea Institute of Oceanology, Chinese Academy of Sciences, 164 West Xingang Road, Guangzhou 510301, China; zhangyun@scsio.ac.cn

**Keywords:** *Arthrinium*, pyridone alkaloid, antibacterial activity, cytotoxicity

## Abstract

Eight new 4-hydroxy-2-pyridone alkaloids arthpyrones D–K (**1**–**8**), along with two known analogues apiosporamide (**9**) and arthpyrone B (**10**), were isolated from a deep-sea-derived fungus *Arthrinium* sp. UJNMF0008. The structures of the isolated compounds were elucidated on the basis of spectroscopic methods with that of **1** being established by chemical transformation and X-ray diffraction analysis. Compounds **1** and **2** bore an ester functionality linking the pyridone and decalin moieties first reported in this class of metabolites, while **3** and **4** incorporated a rare natural hexa- or tetrahydrobenzofuro[3,2-*c*]pyridin-3(2*H*)-one motif. Compounds **3**–**6** and **9** exhibited moderate to significant antibacterial activity against *Mycobacterium smegmatis* and *Staphylococcus aureus* with IC_50_ values ranging from 1.66–42.8 μM, while **9** displayed cytotoxicity against two human osteosarcoma cell lines (U2OS and MG63) with IC_50_ values of 19.3 and 11.7 μM, respectively.

## 1. Introduction

The increasing antibiotic resistance and high spreading rate of pathogenic bacteria have become severe public healthcare threats globally, and the efforts towards new antibacterial drugs remain a pressing task [1]. Marine fungi have been considered to be an invaluable resource for bioactive compounds and play an important role in the search for novel antimicrobial compounds [1,2,3]. Fungi of the genus *Arthrinium* are widely distributed throughout the world and have proven to produce diverse secondary metabolites with a variety of bioactivities, including cytotoxicity [4,5,6,7], antimicrobial [8], anti-HSV [9], AChE inhibitory [4], COX-2 inhibitory [10], syncytium formation inhibitory [11] and Maxi-K channel modulatory [12] activities, as well as lethality against brine shrimp [13].

**Figure 1:**
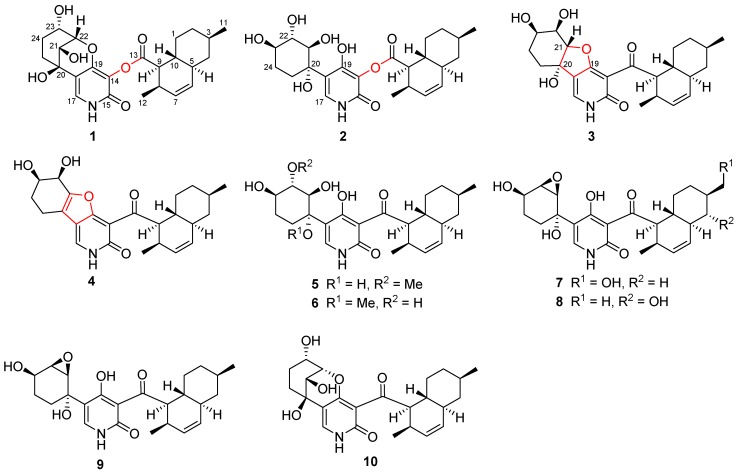
Chemical structures of **1**–**10**.

During the course of our ongoing pursuit for antimicrobial compounds from marine-derived fungi, an *Arthrinium* sp. UJNMF0008 from a deep-sea sediment sample, collected in the South China Sea (17°55′00″ N, 115°55′31″ E; 3858 m depth), came to our attention owing to its strong inhibitory activity against *Staphylococcus aureus.* Further chemical investigation on a large-scale culture of this fungal strain led to the isolation of eight previously unreported 4-hydroxy-2-pyridone alkaloids. arthpyrones D–K (**1**–**8**), together with two previously reported co-metabolites, apiosporamide (**9**) [14,15] and arthpyrone B (**10**) [4]. The structures (Figure 1) of the isolated compounds were characterized on the basis of comprehensive spectroscopic analyses, and their absolute configurations were assigned by different means including ECD comparison, chemical transformation and X-ray crystallography. All the isolates were tested for their antimicrobial, cytotoxic and acetylcholinesterase (AChE) inhibitory activities. The assay results established that **3**–**6** and **9** were mild to significant antibacterial agents against *Mycobacterium smegmatis* and *S. aureus*, while **9** showed moderate cytotoxicity against two human osteosarcoma cell lines U2OS and MG63.

## 2. Results and discussion

### 2.1. Structure Elucidation

**Figure 2:**
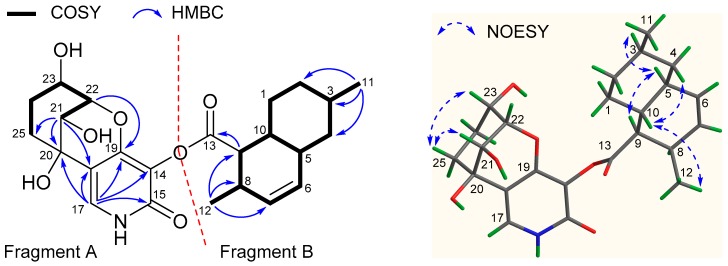
Key 2D NMR correlations for **1**.

Compound **1** was obtained as white powder. The HR-ESIMS ion for **1** at *m*/*z* 444.2032 ([M − H]^−^, calcd. 444.2028) suggested a molecular formula of C_24_H_31_NO_7_, which was 16 amu more than that of its co-metabolite arthpyrone B (**10**) [4], supportive of an oxygenated analogue. Detailed analysis of the ^1^H and ^13^C NMR data (Table 1 and Table 2) indicated that **1** incorporated a skeleton similar to that of arthpyrone B (**10**) [4], with a likely additional ester group as supported by the NMR differences of the remarkably upfield shifted C-13 resonance (from 208.4 in **10** to 172.9 in **1**) and of adjacent C-9, C-14, C-15, C-17 and C-19 signals. This deduction was further confirmed by inspection of 2D NMR data. The COSY correlations (Figure 2) of **1** revealed two spin coupling systems from H_2_-1–H-10 (including branch fragments from H-3–H_3_-11 and H-8–H_3_-12) of a decalin moiety and from H-21–H_2_-25. Subsequent examination of HMBC data (Figure 2) revealed key correlations from H-17 to C-14, C-15, C-18, C-19 and C-20, H-21 to C-18, C-20 and C-25, H-22 to C-19 and H-9 to C-13, leading to the construction of Fragments A and B that were identical to those in **10**. The two fragments were linked via an ester bond as demonstrated by the MS difference (amu 16) and ^13^C NMR chemical shift variations for C-13 (Δ*δ*_C_ −35.5) and C-14 (Δ*δ*_C_ 10.9) between **1** and **10**. The planar structure of **1** was thus established with an ester bridge between the pyridone and decalin moieties.

**Figure 3:**
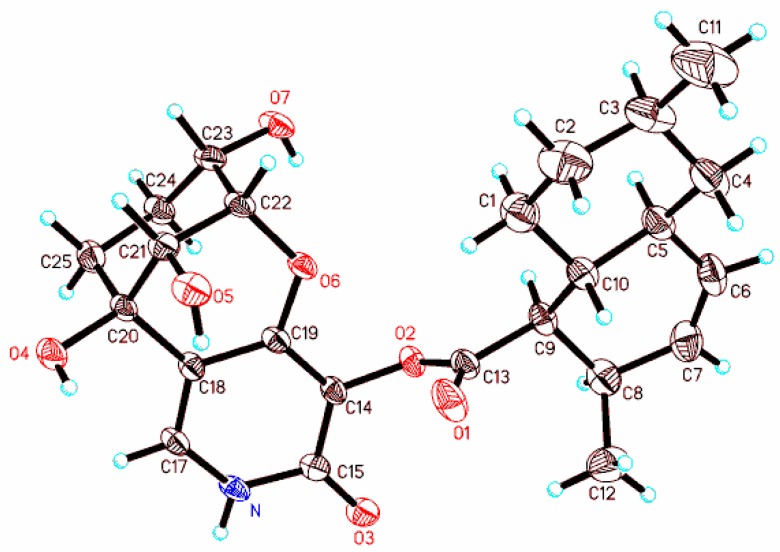
ORTEP view of **1**.

The relative stereochemistry of **1** was assigned to be identical to that of arthpyrone B (**10**) based on analyses of NOESY data and ^1^H–^1^H couplings. More specifically, the key NOESY correlations from H-10 (*δ*_H_ 1.39) to both H-4b (*δ*_H_ 0.78) and H_3_-12 (*δ*_H_ 1.11) (1,3-diaxial relationship) suggested that H-4b, H-10 and CH_3_-12 were axially located on the same side. Meanwhile, the correlations from H-5 (*δ*_H_ 1.80) to both H-3 (*δ*_H_ 1.52) and H-9 (*δ*_H_ 2.90) also indicated their axial and co-facial positions. The relative configuration of Fragment B was thus defined with a *trans*-conjunction. In addition, the relative configuration of Fragment A was supported by the NOESY correlations from H-25a (*δ*_H_ 1.86) to both H-21 (*δ*_H_ 3.74) and H-23 (*δ*_H_ 3.84), as well as *J*_21,22_ (2.4 Hz) and *J*_22,23_ (2.4 Hz). Unfortunately, the stereochemical relationship between Fragments A and B could not be determined due to the interruption of the achiral pyridone unit. However, a further transformation of **10** to **1** (see the Experimental Section) via Baeyer–Villiger oxidation connected the chemical relationship between the two co-metabolites and also confirmed the structural assignment. Finally, a suitable single crystal of **1** was obtained from the MeOH-H_2_O binary system and subject to X-ray diffraction analysis, which not only verified the structure of **1**, but also established its absolute stereochemistry (Figure 3) as 3*R*, 5*S*, 8*R*, 9*R*, 10*R*, 20*R*, 21*S*, 22*S*, 23*S* (Flack parameter, 0.0(3)). Compound **1** was thus unambiguously characterized and was named arthpyrone D, following arthpyrones A−C, isolated from another fungus of the same genus [4]. It is interesting to point out that the assigned C-20 configuration for **1** is opposite that described for arthpyrones A and B [4]. By careful examination of the reported ORTEP view of arthpyrone A, it is clear that both arthpyrones A and B also bear a 20*R* absolute configuration, while the authors inverted the C-20 stereochemistry by mistake when drawing the structures in two dimensions.

**Figure 4:**
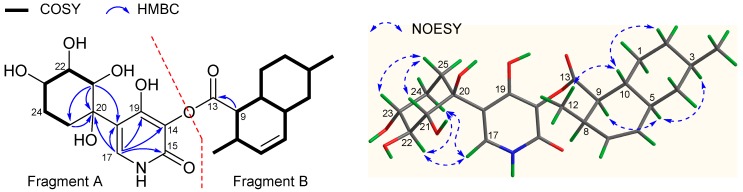
Key 2D NMR correlations for **2**.

Compound **2** was isolated as a pale yellow powder, and its molecular formula C_24_H_33_NO_8_ was established from the HR-ESIMS ion at *m*/*z* 462.2143 ([M − H]^−^, calcd. 462.2133), being 18 mass units more than that of **1**. The NMR data of **2** resembled those of **1** (Table 1 and Table 2), and the obvious differences were the downfield shifted signals of C-20 (*δ*_C_ 78.5 in **2** and 70.6 in **1**) and C-21 (*δ*_C_ 77.3 in **2** and *δ*_C_ 70.0 in **1**), along with the upfield shifted resonance of C-22 (*δ*_C_ 75.3 in **2** and *δ*_C_ 85.2 in **1**). Further examination of COSY data (Figure 4) revealed two structural motifs of the decalin and CH-21–CH_2_-25 part, which were the same as those in **1**. The pivotal HMBC correlations (Figure 4) from H-9 (*δ*_H_ 2.88) to C-13, H-17 (*δ*_H_ 7.31) to C-14, C-15, C-18, C-19 and C-20 and H-21 (*δ*_H_ 3.71) to C-18, C-20 and C-25 were also consistent with those observed for **1**. Therefore, the aforementioned MS and NMR differences, the absence of HMBC correlation from H-22 to C-19 and the more polar nature of **2** all supported the planar structure of **2** without the epoxy bridge between C-19 and C-22. The relative configuration of **2** was assigned on the basis of NOESY data (Figure 4). The configuration of the decalin moiety was identical to that of **1** as indicated by NOESY correlations and similar NMR data. The NOESY correlations from H-25b (*δ*_H_ 1.67) to both H-21 (*δ*_H_ 3.71) and H-23 (*δ*_H_ 3.69) suggested their axial and co-planar nature, while those from H-22 (*δ*_H_ 3.62) to both H-17 (*δ*_H_ 7.31) and H-24b (*δ*_H_ 1.54) supported that H-22, H-24b and the pyridone unit were axially located on the other side of the hexane ring. The structure of **2** was hence elucidated as shown in Figure 4.

**Figure 5:**
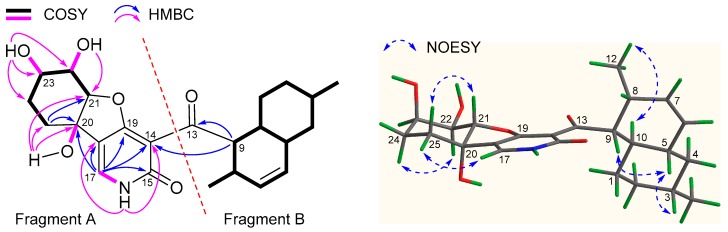
Key 2D NMR correlations for **3** (pink correlations observed in DMSO-*d*_6_).

Compound **3** was assigned a molecular formula of C_24_H_31_NO_6_ by HR-ESIMS *m*/*z* 428.2074 ([M − H]^−^, calcd. 428.2079), suggestive of an isomer of apiosporamide (**9**) [14,15]. Analysis of the NMR data (Table 1 and Table 2) for **3** revealed the presence of a decalin moiety as indicated by a close resemblance between its NMR data and those of **9**, and the main NMR differences between the two co-metabolites observed for the left-side Fragment A. Further inspection of COSY data (Figure 5) confirmed the existence of the decalin motif and also established the same spin coupling system from H-21–H_2_-25 as that in **9**. In addition, the HMBC correlations (Figure 5) from H-9 (*δ*_H_ 4.44) to C-13 and C-14 verified the connection between Fragments A and B, while those from H-17 (*δ*_H_ 7.69) to C-14, C-15, C-18, C-19 and C-20, along with those from H-25b (*δ*_H_ 1.51) to C-20 and C-21, enabled the assembly of the pyridone ring and the cyclohexane moiety, as well as their linkage from C-18–C-20. Moreover, four exchangeable protons were observed in the ^1^H NMR spectrum (Supplementary Information Table S1), measured in DMSO-*d*_6_, which were assigned for NH, 20-OH, 22-OH and 23-OH, respectively. This was supported by HMBC and COSY correlations (Figure 5) from N*H* to H-17, 20-OH to C-20, H-22 to 22-O*H* and H-23 to 23-O*H*. The above-mentioned analyses accounted for all but one oxygen atom, which was ascribed to the formation of an epoxy bridge between C-19 and C-21, as was also supported by the downfield shifted chemical shift of C-19 (*δ*_C_ 177.2) and upfield shifted chemical shift of C-21 (*δ*_C_ 67.7) compared with those in **2** (Table 2). The relative configuration of **3** was established via analyses of NOESY data (Figure 5) and ^1^H–^1^H couplings (Table 1). The similarity between the ^1^H NMR spectra of the decalin moiety of **3** and **9** supported the common relative stereochemistry, as was also confirmed by NOESY correlations. The crucial NOESY correlations from H-21–H-25a and H-22–H-24a indicated the 1,3-diaxial relationship of the two proton pairs, which in combination with the proton coupling constants of *J*_21_,_22_ (10.1 Hz) and *J*_22_,_23_ (2.9 Hz) defined the relative configuration of the polyoxygenated cyclohexane moiety. The structure of **3** was thereby characterized to feature a rare hexahydrobenzofuro[3,2-*c*]pyridin-3(2*H*)-one fragment with very few examples reported from nature [16,17,18].

The molecular formula of **4** was determined as C_24_H_29_NO_5_ by HR-ESIMS *m*/*z* 410.1974 ([M − H]^−^, calcd. 410.1973), indicative of a dehydrated analogue of **3**. Analysis of the NMR data (Table 1 and Table 2) for **4** confirmed this hypothesis, with diagnostic signals for two quaternary sp^2^ carbons (*δ*_C_ 116.6 and 156.2) in **4** instead of the oxyquaternary sp^3^ carbons at *δ*_C_ 76.9 (C-20) and *δ*_C_ 67.7 (C-21) in **3**. This observation led to the suggestion that the furan ring is formed between the cyclohexadiol and 2-piperidone rings and extended the conjugation system of the pyridone chromophore, which was further confirmed by the UV data of **4** with an absorption peak red-shifted to 367 nm. Further examination of COSY and HMBC correlations (Supplementary Information Figures S42 and S43) corroborated the aforementioned structural assignment. The similarity of the NMR data of the decalin moieties of **4** and **3** suggested the common relative configurations as also confirmed by NOESY correlations (Supplementary Information Figure S44). The crucial NOESY correlation from H-23–H-25b and proton coupling constants of *J*_22_,_23_ (3.8 Hz) and *J*_23_,_24a_ (10.8 Hz) defined the relative configurations at C-22 and C-23 as shown in Figure 1.

Compounds **5** and **6** were assigned the same molecular formula of C_25_H_35_NO_7_ by their HR-ESIMS *m*/*z* 460.2349 and 460.2337 ([M − H]^−^, calcd. 460.2341), respectively, suggestive of a pair of isomers. The NMR data (Table 2 and Table 3) for **5** revealed high similarity to those of apiosporamide (**9**) [14,15], and the major differences were attributable to the shielded signals for the oxirane ring in **9** instead of two oxymethines (*δ*_H_ 3.87 and 3.35; *δ*_C_ 78.7 and 86.6) and a methoxy group (*δ*_H_ 3.63; *δ*_C_ 60.5) in **5**. Subsequent analysis of COSY and HMBC correlations (Supplementary Information Figures S50 and S51) corroborated the aforementioned observations with a key HMBC correlation from the methoxy protons to C-22 (*δ*_C_ 86.6) and established the planar structure of **5**. The relative stereochemistry of **5** was determined to be identical to that of **2** based on the comparison of their NMR spectra, including crucial ^1^H–^1^H couplings between the corresponding chiral centers, which were further confirmed by examination of the NOESY spectrum (Supplementary Information Figure S52). Analysis of the NMR data (Table 2 and Table 3) for **6** suggested that it was a methoxy regio-isomer of **5**, and the methoxy group was determined to locate at C-20 (*δ*_C_ 82.4) via the HMBC correlation from the methoxy protons (*δ*_H_ 3.18) to C-20. Detailed inspection of COSY and HMBC spectra (Supplementary Information Figures S57 and S58) verified the planar structure of **6**, and the relative configuration was also determined to be the same as that of **5** based on analyses of key ^1^H–^1^H couplings and NOESY correlations (Supplementary Information Figure S59).

Compounds **7** and **8** were determined to possess the same molecular formula C_24_H_31_NO_7_ by their HR-ESIMS *m*/*z* 444.2025 and 444.2030 ([M − H]^−^, calcd. 444.2028), respectively, indicative of oxygenated analogues of apiosporamide (**9**) [14,15]. Analysis of the NMR data (Table 2 and Table 3) for **7** confirmed this hypothesis with diagnostic signals for an oxymethylene (*δ*_H_ 3.39, 2H; *δ*_C_ 68.6) in **7** instead of the CH_3_-11 group in **9**. Good resemblance between the remaining NMR data for **7** and **9** suggested the assignment of common structural features between the two co-metabolites and indicated their same relative stereochemistry. The structure with the relative configuration of **7** was finally confirmed by further examination of 2D NMR data, especially COSY, HMBC and NOESY spectra (Supplementary Information Figures S64–S66). Similarly, the oxidation of **8** occurred at C-4 as supported by the COSY correlations from H-4 (*δ*_H_ 2.73) to H-3 (*δ*_H_ 1.39) and H-5 (*δ*_H_ 1.77), as well as the HMBC correlation from H_3_-11 (*δ*_H_ 1.03) to C-4 (*δ*_C_ 79.6). The coupling constants of H-4 (dd, *J* = 9.9 Hz) indicated that it was axially located, and thus, the hydroxyl group was equatorially oriented. The low chemical shift of H-4 (<3.0 ppm) was due to its axial position, which located it in the shielding zone of C2-C3 and C5-C6 bonds. The similarity between the remaining NMR data for **8** and **7**, especially key ^1^H–^1^H coupling constants, supported common structural features, including configurations of the stereocenters, between the two co-metabolites. The structure of **8** with the relative configuration was further confirmed by analysis of 2D NMR data (Supplementary Information Figures S71–S73).

**Figure 6:**
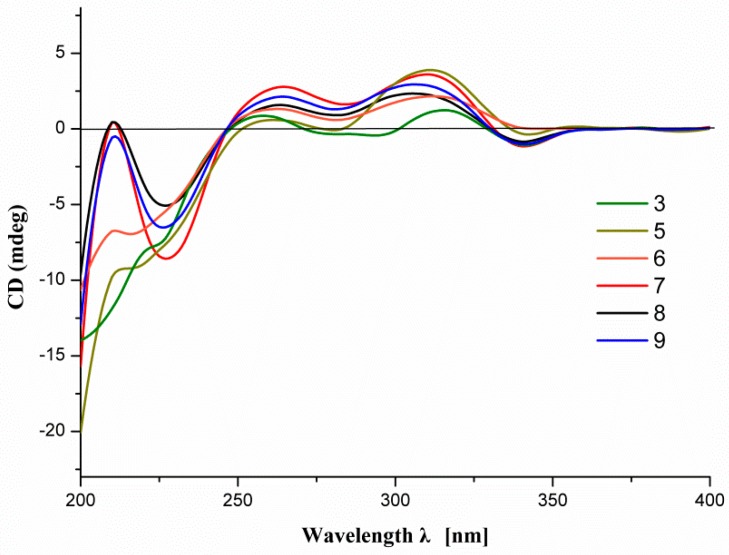
The ECD spectra of **3** and **5**–**9**.

**Scheme 1:**
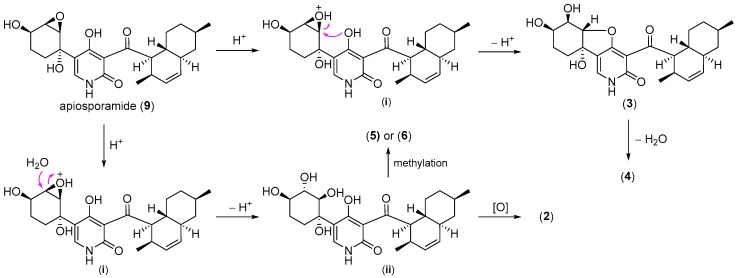
Proposed biosynthetic pathway for **2**–**6**.

Compounds **9** and **10** were identified to be the previously reported apiosporamide [14,15] and arthpyrone B [4], respectively, based on comprehensive spectroscopic analyses including MS, NMR, [α]_D_ and ECD. While the absolute configuration of **1** was established by X-ray analysis and chemical transformation, those of the other analogues were assigned mainly by analysis of their ECD data. The absolute configurations of **3** and **5**–**8**, which have the same 3-acyl pyridone chromophore, were characterized by their ECD spectra, which are similar to that of **9** (Figure 6), whose absolute structure had been confirmed by total synthesis of its enantiomer [14,15]. The absolute configurations of the stereocenters of **2** and **4** were proposed on biogenetic grounds (Scheme 1).

With all these metabolites in hand, we were able to propose a plausible biosynthetic pathway for the new analogues. Compound **1** could be obtained from **10** via one-step Baeyer–Villiger oxidation, while selective oxidation of **9** at C-11 and C-4 would afford **7** and **8**, respectively. The likely biosynthetic origin of **2**–**6** is outlined in Scheme 1. Protonation of the epoxy group of **9** would give the intermediate (**i**) that could undergo intramolecular nucleophilic substitution by 19-OH, followed by deprotonation to yield **3**. Subsequent dehydration of **3** would furnish **4**. The intermediate (**i**) could also experience ring opening by hydrolysis followed by deprotonation to afford the intermediate (**ii**), which was not obtained in the current study. The intermediate (**ii**) could produce **2** via Baeyer–Villiger oxidation or undergo selective methylation to yield **5** or **6**.

### 2.2. Biological Activity

Compounds **1**–**10** were tested against two Gram-positive strains *Mycobacterium smegmatis* ATCC 607 and *Staphylococcus aureus* ATCC 25923, two Gram-negative strains *Escherichia coli* ATCC 8739 and *Pseudomonas aeruginosa* ATCC 9027 and a yeast *Candida albicans* ATCC10231. Compounds **3**–**6** and **9** exhibited antibacterial activity against *M. smegmatis* and *S. aureus* with IC_50_ values ranging from 1.66–42.8 μM (Table 4), while none of the tested compounds showed significant antimicrobial activity against *E. coli*, *P. aeruginosa* and *C. albicans* at 50 μM. Expanding our biological activity evaluations, these isolates were also screened for AChE inhibitory and cytotoxic activities against two human osteosarcoma cell lines U2OS and MG63. Only **9** displayed mild cytotoxicity against the two cell lines with IC_50_ values of 19.3 and 11.7 μM.

## 3. Experimental Section

### 3.1. General Experimental Procedures

NMR spectra were recorded on a Bruker Avance DRX600 NMR spectrometer with residual solvent peaks as references (CD_3_OD: δ_H_ 3.31, δ_C_ 49.00; DMSO-*d*_6_: δ_H_ 2.50, δ_C_ 39.52). Optical rotations were measured on a Rudolph VI polarimeter. UV spectra were obtained using a Shimadzu UV-2600 spectrophotometer. The X-ray diffraction analysis was performed on an Oxford Gemini E Eos CCD detector with graphite monochromated Cu K*α* radiation (*λ* = 1.54178 Å). ECD spectra were acquired on a Chirascan circular dichroism spectrometer (Applied Photophysics). ESIMS analyses were conducted on an Agilent 1260–6460 Triple Quad LC-MS spectrometer. HR-ESIMS spectra were obtained on an Agilent 6545 Q-TOF mass spectrometer. Semipreparative HPLC separations were carried out on an Agilent 1260 series using a YMC-Pack ODS-A column (250 × 10 mm, 5 μm). Column chromatography (CC) was performed on silica gel (200–300 mesh, Yantai Jiangyou Silica Gel Development Co., Yantai, China), reversed phase C_18_ silica gel (Merck KGaA, Darmstadt, Germany) and Sephadex LH-20 gel (GE Healthcare Bio-Sciences AB, Uppsala, Sweden).

### 3.2. Fungal Material

The fungus strain UJNMF0008 was isolated from a marine sediment sample collected in the South China Sea (17°55′00″ N, 115°55′31″ E; 3858 m depth). This strain was identified as an *Arthrinium* sp. based on morphological traits and a molecular biological protocol by DNA amplification and comparison of its ITS region sequence with the GenBank database (100% similarity with *Arthrinium* sp. zzz1842 (HQ696050.1)). The BLAST sequenced data were deposited at GenBank (No. MG010382). The strain was deposited at China General Microbiological Culture Collection Center (CGMCCC), Institute of Microbiology, Chinese Academy of Sciences.

### 3.3. Fermentation and Extraction

*Arthrinium* sp. UJNMF0008 from a PDA culture plate was inoculated in 500-mL Erlenmeyer flasks containing 150 mL soluble starch medium (1% glucose, 0.1% soluble starch, 1% MgSO_4_, 0.1% KH_2_PO_4_, 0.1% peptone and 3% sea salt) at 28 °C on a rotary shaker at 180 rpm for 3 days as seed cultures. Then, each of the seed cultures (20 mL) was transferred into autoclaved 1-L Erlenmeyer flasks with solid rice medium (each flasks contained 80 g commercially available rice, 0.4 g yeast extract, 0.4 g glucose, and 120 mL water with 3% sea salt). After that, the strain was incubated statically for 30 days at 28 °C.

After fermentation, the total 4.8 kg rice culture was crushed and extracted with 15.0 L 95% EtOH three times. The EtOH extract was evaporated under reduced pressure to afford an aqueous solution and then extracted with 2.0 L ethyl acetate three times to give 80 g crude gum.

### 3.4. Isolation and Purification

The extract was fractionated by a silica gel column eluting with step gradient CH_2_Cl_2_-MeOH (*v*/*v* 100:0, 98:2, 95:5, 90:10, 80:20, 70:30, 50:50 and 0:100, each 8.0 L) to give ten fractions (Fr.1–Fr.10) based on TLC and HPLC analysis. Fr.7 (12.2 g) was repeatedly applied to the silica gel column with step gradient CH_2_Cl_2_-(CH_3_)_2_CO (*v*/*v* 100:0–0:100) and divided into three subfractions (Fr.7-1–Fr.7-3). Fr.7-2 (5.8 g) was fractionated by the MCI gel column eluted with gradient MeOH-H_2_O to give four subfractions (Fr.7-2-1–Fr.7-2-4). Fr.7-2-4-1 (68.5 mg) obtained from Fr.7-2-4 (0.9 g) via Sephadex LH-20 CC (in MeOH) was further purified by HPLC eluting with MeOH-H_2_O (*v*/*v* 73:27, 2.5 mL min^−1^) to give **9** (t_R_ = 34.6 min, 45.2 mg), while Fr.7-2-4-2 (40.2 mg) was further separated by HPLC eluting with MeOH-H_2_O (*v*/*v* 71:29, 2.5 mL min^−1^) to afford **4** (t_R_ = 22.2 min, 2.8 mg), **3** (t_R_ = 32.3 min, 1.6 mg) and **5** (t_R_ = 48.3 min, 2.5 mg). Fr.8 (15.9 g) was subject to silica gel CC with step gradient CH_2_Cl_2_-(CH_3_)_2_CO (*v*/*v* 100:0–0:100) and divided into eight subfractions (Fr.8-1–Fr.8-8). Fr.8-2 (3.2 g) was applied to the MCI column eluted with gradient MeOH-H_2_O to give four subfractions (Fr.8-2-1–Fr.8-2-4). Fr.8-2-3 (0.9 g) was further divided by Sephadex LH-20 CC eluting with MeOH-CH_2_Cl_2_ (*v*/*v* 1:1) and then purified by HPLC eluting with MeOH-H_2_O-CH_3_CO_2_H (*v*/*v*/*v* 50:50:10^−4^, 2.5 mL min^−1^) to afford **7** (t_R_ = 21.1 min, 48.2 mg) and **8** (t_R_ = 23.9 min, 54.6 mg). Fr.8-2-4 (432.4 mg) was also fractionated by Sephadex LH-20 CC eluting with MeOH-CH_2_Cl_2_ (*v*/*v* 1:1) and then purified by HPLC eluting with MeOH-H_2_O-CH_3_CO_2_H (*v*/*v*/*v* 75:25:10^−4^, 2.5 mL min^−1^) to yield **6** (t_R_ = 33.1 min, 1.6 mg). Fr.8-3 (6.4 g) was applied to MPLC with an ODS column eluting with gradient MeOH-H_2_O (*v*/*v* 50:50–70:30) to obtain **1** (3.3 g). Fr.8-8 (190.0 mg) was separated by Sephadex LH-20 CC eluting with MeOH-CH_2_Cl_2_ (*v*/*v* 1:1) to obtain two subfractions (Fr.8-8-1–Fr.8-8-2). Then, Fr.8-8-1 (122.6 mg) was purified by HPLC eluting with MeOH-H_2_O-CH_3_CO_2_H (*v*/*v*/*v* 64:36:10^−4^, 2.5 mL min^−1^) to yield **10** (t_R_ = 33.2 min, 52.6 mg), and Fr.8-8-2 (35.6 mg) was isolated by HPLC eluting with MeOH-H_2_O-CH_3_CO_2_H (*v*/*v*/*v* 68:32:10^−4^, 2.5 mL min^−1^) to yield **2** (t_R_ = 13.5 min, 4.6 mg).

#### 3.4.1. Arthpyrone D (**1**)

Colorless crystals; mp 204–207 °C; [α]^26^_D_ −23.8 (*c* 1.0, MeOH); UV (MeOH) *λ*_max_ (log ε) 213 (3.96), 289 (3.04) nm; ECD (0.05 mg mL^−1^, MeOH) *λ* (Δε) 214 (46.2), 235 (3.4), 283 (1.6) nm; ^1^H and ^13^C NMR data, Table 1 and Table 3; (+)-ESIMS *m*/*z* 446.1 [M + H]^+^; (−)-HR-ESIMS *m*/*z* 444.2032 [M − H]^−^ (calcd. for C_24_H_30_NO_7_, 444.2028).

#### 3.4.2. Arthpyrone E (**2**)

Pale yellow powder; [α]^26^_D_ −28.9 (*c* 0.5, MeOH); UV (MeOH) *λ*_max_ (log ε) 211 (3.96), 279 (3.02) nm; ECD (0.05 mg mL^−1^, MeOH) *λ* (Δε) 212 (27.0), 285 (−1.1) nm; ^1^H and ^13^C NMR data, Table 1 and Table 3; (−)-ESIMS *m*/*z* 462.0 [M − H]^−^; (−)-HR-ESIMS *m*/*z* 462.2143 [M − H]^−^ (calcd. for C_24_H_32_NO_8_, 462.2133). 

#### 3.4.3. Arthpyrone F (**3**)

Pale yellow powder; [α]^26^_D_ −108.7 (*c* 0.2, MeOH); UV (MeOH) *λ*_max_ (log ε) 230 (2.87), 279 (2.35), 332 (2.56) nm; ECD (0.20 mg mL^−1^, MeOH) *λ* (Δε) 258 (0.6), 316 (0.8), 341 (−0.7) nm; ^1^H and ^13^C NMR data, Table 1 and Table 3; (−)-ESIMS *m*/*z* 428.0 [M − H]^−^; (−)-HR-ESIMS *m*/*z* 428.2074 [M − H]^−^ (calcd. for C_24_H_30_NO_6_, 428.2079). 

#### 3.4.4. Arthpyrone G (**4**)

Pale yellow powder; [α]^26^_D_ −76.2 (*c* 0.2, MeOH); UV (MeOH) *λ*_max_ (log ε) 228 (3.65), 262 (3.01), 367 (2.67) nm; ECD (0.10 mg mL^−1^, MeOH) *λ* (Δε) 238 (2.5); 319 (0.7) nm; ^1^H and ^13^C NMR data, Table 1 and Table 3; (−)-ESIMS *m*/*z* 410.0 [M − H]^−^; (−)-HR-ESIMS *m*/*z* 410.1974 [M − H]^−^ (calcd. for C_24_H_28_NO_5_, 410.1973).

#### 3.4.5. Arthpyrone H (**5**)

Pale yellow powder; [α]^26^_D_ −90.2 (*c* 0.2, MeOH); UV (MeOH) *λ*_max_ (log ε) 230 (3.07), 277 (2.56), 328 (2.73) nm; ECD (0.20 mg mL^−1^, MeOH) *λ* (Δε) 262 (0.4), 311 (2.7) nm;^1^H and ^13^C NMR data, Table 2 and Table 3; (−)-ESIMS *m*/*z* 460.1 [M − H]^−^; (−)-HR-ESIMS *m*/*z* 460.2349 [M − H]^−^ (calcd. for C_25_H_34_NO_7_, 460.2341).

#### 3.4.6. Arthpyrone I (**6**)

Pale yellow powder; [α]^26^_D_ −76.2 (*c* 0.1, MeOH); UV (MeOH) *λ*_max_ (log ε) 230 (3.14), 278 (2.51), 329 (2.78) nm; ECD (0.10 mg mL^−1^, MeOH) *λ* (Δε) 262 (1.8), 312 (3.0) nm; ^1^H and ^13^C NMR data, Table 2 and Table 3; (−)-ESIMS *m*/*z* 460.1 [M − H]^−^; (−)-HR-ESIMS *m*/*z* 460.2337 [M − H]^−^ (calcd. for C_25_H_34_NO_7_, 460.2341).

#### 3.4.7. Arthpyrone J (**7**)

Pale yellow powder; [α]^26^_D_ −61.2 (*c* 1.0, MeOH); UV (MeOH) *λ*_max_ (log ε) 230 (3.72), 276 (3.17), 330 (3.43) nm; ECD (0.05 mg mL^−1^, MeOH) *λ* (Δε) 210 (1.2), 227 (−23.1), 265 (7.5), 310 (9.7), 341 (−3.1) nm; ^1^H and ^13^C NMR data, Table 2 and Table 3; (−)-ESIMS *m*/*z* 444.0 [M − H]^−^; (−)-HR-ESIMS *m*/*z* 444.2025 [M − H]^−^ (calcd. for C_24_H_30_NO_7_, 444.2028).

#### 3.4.8. Arthpyrone K (**8**)

Pale yellow powder; [α]^26^_D_ −68.7 (*c* 0.5, MeOH); UV (MeOH) *λ*_max_ (log ε) 229 (3.50), 277 (2.98), 330 (3.18) nm; ECD (0.05 mg mL^−1^, MeOH) *λ* (Δε) 210 (1.2), 227 (−13.7), 263 (4.3), 306 (6.3), 342(−2.3) nm; ^1^H and ^13^C NMR data, Table 2 and Table 3; (−)-ESIMS *m*/*z* 444.0 [M − H]^−^; (−)-HR-ESIMS *m*/*z* 444.2030 [M − H]^−^ (calcd. for C_24_H_30_NO_7_, 444.2028). 

### 3.5. Chemical Transformation of **10** to **1**

M-chloroperoxybenzoic acid (MCPBA) (31.1 mg, 0.18 mmol) was added to a solution of **10** (25.3 mg, 0.06 mmol) in methanol (2 mL), and the mixture was stirred at 25 °C overnight. Then, the reaction solution was concentrated under reduced pressure, and the residue was purified by HPLC eluting with MeOH-H_2_O (*v*/*v* 60:40, 2.5 mL min^−1^) to yield **10r** (t_R_ = 49.7 min, 0.8 mg), which was consistent with **1** in all aspects.

### 3.6. Antimicrobial Assays

The antimicrobial activity was assayed against the Gram-positive bacterial strains *Mycobacterium smegmatis* ATCC 607 and *Staphylococcus aureus* ATCC 25923, Gram-negative *Escherichia coli* ATCC 8739 and *Pseudomonas aeruginosa* ATCC 9027 and yeast *Candida albicans* ATCC10231 by liquid growth inhibition in 96-well microplates. Briefly, precultures of the tested microorganisms were made by inoculating 10 mL of medium (LB medium for bacteria and YM medium for fungus) and incubated for 24 h at 37 °C for bacteria or 48 h at 28 °C for fungus. Then, the cell density was adjusted to 10^4^−10^5^ cfu mL^−1^ by the corresponding broth. An aliquot of 200 μL of the microbial suspension was distributed in each well containing 2-fold serial dilution of the tested compounds. The plate was incubated at 37 °C for 12 h for the bacteria or at 28 °C for 48 h for the fungus, and the optical density of each well was measured at 600 nm spectrophotometrically. IC_50_ values were defined as the concentration of a compound resulting in a 50% decrease in the number of microbial cultures compared to the blank control. The experiment was run in three replicates.

### 3.7. AChE Inhibitory Assay

The AChE inhibitory activities of compounds were tested by using the modified Ellman’s method as described previously [19].

### 3.8. Cytotoxic Assay

The cytotoxicity of compounds was evaluated using human osteosarcoma U2OS and MG63 cell lines by the MTT method as described previously [20]. Adriamycin was used as a positive control (IC_50_ 21.1 and 3.56 nM for U2OS and MG63, respectively).

### 3.9. X-ray Diffraction Analysis of Arthpyrone D (**1**)

Compound **1** was crystallized in MeOH-H_2_O at room temperature. The X-ray crystallographic data were obtained from an Oxford Gemini E Eos CCD detector equipped with a graphite monochromated Cu K*α* radiation (λ = 1.54178 Å) at 293(2) K. The structure was solved with the XT structure solution program using intrinsic phasing and refined with the ShelXL refinement package using least squares minimization [21,22]. All non-hydrogen atoms were refined anisotropically. The hydrogen atom positions were geometrically idealized and allowed to ride on their parent atoms. Crystallographic data for **1** were deposited at the Cambridge Crystallographic Data Centre (Deposition No.: CCDC 1813453).

## 4. Conclusions

Until now, about twenty 4-hydroxy-2-pyridone alkaloids with a decalin ring have been reported and showed significant cytotoxicity, antimicrobial activity and AChE inhibitory activity, but only arthpyrones A–C were isolated from marine-derived fungus [6,14,15,23,24,25,26,27,28,29,30]. Our current study contributed eight new members (**1**–**8**) to this structural class from a deep-sea-derived fungus *Arthrinium* sp. Among this series of fungal metabolites, the ester bridge that connected the pyridone and decalin fragments in **1** and **2** was reported for the first time, while **3** and **4** incorporating the rare hexa- or tetrahydrobenzofuro[3,2-*c*]pyridin-3(2*H*)-one ring system were also the first examples of this class of compounds. Our biological activity evaluations established selective compounds as antibacterial agents against Gram-positive *M. smegmatis* and *S. aureus* and/or cytotoxic agents toward human osteosarcoma cell lines U2OS and MG63. Knowledge of these rare pyridone alkaloids will be of great interest to the scientific community.

## Figures and Tables

**Table 1:** ^1^H NMR (600 MHz) data for **1**–**4** (CD_3_OD).

Positon	1	2	3	4
	*δ*_H_, mult. (*J* in Hz)	*δ*_H_, mult. (*J* in Hz)	*δ*_H_, mult. (*J* in Hz)	*δ*_H_, mult. (*J* in Hz)
1a	2.11, m	2.07, m	1.93, m	1.98, m
1b	1.08, m	1.07, m	0.90, m	0.96, m
2a	1.78, m	1.79, m	1.76, m	1.75, m
2b	1.01, m	1.01, m	1.05, m	1.03, m
3	1.52, m	1.54, m	1.52, m	1.49, m
4a	1.74, m	1.77, m	1.76, m	1.73, m
4b	0.78, q (12.2)	0.78, q (12.4)	0.81, q (12.3)	0.79, q (12.4)
5	1.80, m	1.82, m	1.83, m	1.84, m
6	5.41, brd (9.9)	5.41, brd (9.9)	5.41, brd (9.8)	5.38, brd (9.8)
7	5.61, ddd (9.9, 4.5, 2.8)	5.62, ddd (9.9, 4.4, 2.8)	5.61,m	5.57, ddd (9.8, 4.5, 2.7)
8	2.76, m	2.79, m	2.84, m	2.73, m
9	2.90, dd (11.7,5.9)	2.88, dd (11.6, 5.9)	4.44, dd (11.3, 5.8)	4.07, dd (11.3,5.7)
10	1.39, qd (11.3, 2.5)	1.38, qd (11.5, 2.3)	1.57, qd (11.2, 2.0)	1.54, qd (11.3, 2.6)
11	0.93, d (6.6)	0.93, d (6.6)	0.94, d (6.5)	0.92, d (6.5)
12	1.11, d (7.0)	1.08, d (7.1)	0.83, d (7.2)	0.76, d (7.1)
17	7.38, s	7.31, s	7.69, s	7.96, s
21	3.74, d (2.4)	3.71, d (7.0)	5.15, d (10.1)	
22	4.60, brs	3.62, dd (7.0, 6.2)	3.91, dd (10.1, 2.9)	4.68, d (3.8)
23	3.84, ddd (11.6, 5.1, 2.4)	3.69, m	4.09, m	3.95, ddd (10.8, 3.8, 3.2)
24a	1.86, m	2.12, m	2.07, m	2.03, m
24b	1.34, m	1.54, m	1.72, m	1.89, m
25a	1.86, m	2.43, ddd (14.1, 7.9, 3.7)	2.88, m	2.69, m
25b	1.75, m	1.67, ddd (14.1, 9.3, 3.8)	1.51, m	2.51, m

**Table 2:** ^13^C NMR (150 MHz) data for **1**–**8** (CD_3_OD).

Position	1	2	3	4	5	6	7	8
	*δ*_c_, Type	*δ*_c_, Type	*δ*_c_, Type	*δ*_c_, Type	*δ*_c_, Type	*δ*_c_, Type	*δ*_c_, Type	*δ*_c_, Type
1	30.7, CH_2_	30.8, CH_2_	31.0, CH_2_	30.8, CH_2_	31.0, CH_2_	31.0, CH_2_	30.4, CH_2_	30.4, CH_2_
2	36.5, CH_2_	36.5, CH_2_	36.6, CH_2_	36.7, CH_2_	36.6, CH_2_	36.6, CH_2_	30.8, CH_2_	34.6, CH_2_
3	34.3, CH	34.3, CH	34.4, CH	34.4, CH	34.4, CH	34.4, CH	42.0, CH	41.7, CH
4	43.0, CH_2_	43.0, CH_2_	43.2, CH_2_	43.2, CH_2_	43.2, CH_2_	43.2, CH_2_	37.4, CH_2_	79.6, CH
5	43.2, CH	43.2, CH	43.2, CH	43.4, CH	43.2, CH	43.2, CH	42.7, CH	49.8, CH
6	131.6, CH	131.6, CH	131.7, CH	131.7, CH	131.7, CH	131.7, CH	131.6, CH	127.1, CH
7	132.2, CH	132.2, CH	132.7, CH	132.7, CH	132.7, CH	132.6, CH	132.8, CH	133.3, CH
8	33.8, CH	33.7, CH	32.4, CH	32.9, CH	32.5, CH	32.5, CH	32.4, CH	32.0, CH
9	50.3, CH	50.3, CH	54.2, CH	55.5, CH	54.3, CH	54.3, CH	54.2, CH	54.1, CH
10	37.7, CH	37.7, CH	37.6, CH	38.0, CH	37.6, CH	37.6, CH	37.9, CH	35.9, CH
11	22.9, CH_3_	22.9, CH_3_	23.0, CH_3_	23.0, CH_3_	23.0, CH_3_	23.0, CH_3_	68.6, CH_2_	19.3, CH_3_
12	18.3, CH_3_	18.2, CH_3_	18.3, CH_3_	18.3, CH_3_	18.4, CH_3_	18.4, CH_3_	18.4, CH_3_	18.3, CH_3_
13	172.9, C	173.2, C	212.1, C	203.8, C	211.9, C	211.9, C	211.8, C	211.6, C
14	123.8, C	126.4, C	108.1, C	110.7, C	108.9, C	108.5, C	108.7, C	108.8, C
15	158.2, C	160.7, C	164.3, C	163.4, C	163.7, C	164.0, C	163.9, C	164.0, C
17	129.8, CH	132.2, CH	141.0, CH	133.3, CH	143.2, CH	144.2, CH	139.9, CH	139.9, CH
18	115.6, C	115.7, C	118.3, C	115.4, C	114.3, C	111.4, C	116.6, C	116.7, C
19	160.8, C	161.9, C	177.2, C	166.4, C	179.8, C	179.5, C	179.3, C	179.4, C
20	70.6, C	78.5, C	76.9, C	116.6, C	76.3, C	82.4, C	70.4, C	70.4, C
21	70.0, CH	77.3, CH	67.7, CH	156.2, C	78.7, CH	75.2, CH	60.5, CH	60.5, CH
22	85.2, CH	75.3, CH	74.1, CH	65.3, CH	86.6, CH	75.2, CH	57.6, CH	57.6, CH
23	71.6, CH	72.6, CH	71.3, CH	70.8, CH	72.4, CH	72.4, CH	67.1, CH	67.1, CH
24	27.7, CH_2_	27.6, CH_2_	27.1, CH_2_	26.7, CH_2_	28.2, CH_2_	26.1, CH_2_	25.8, CH_2_	25.8, CH_2_
25	37.6, CH_2_	31.1, CH_2_	31.2, CH_2_	18.4, CH_2_	30.4, CH_2_	23.7, CH_2_	31.6, CH_2_	31.6, CH_2_
OCH_3_					60.5, CH_3_	51.0, CH_3_		

**Table 3:** ^1^H NMR (600 MHz) data for **5**–**8** (CD_3_OD).

Positon	5	6	7	8
	*δ*_H_, mult. (*J* in Hz)	*δ*_H_, mult. (*J* in Hz)	*δ*_H_, mult. (*J* in Hz)	*δ*_H_, mult. (*J* in Hz)
1a	1.91, m	1.91, m	1.98, m	1.86, m
1b	0.87, m	0.88, m	0.90, m	0.90, m
2a	1.74, m	1.74, m	1.81, m	1.74, m
2b	1.03, m	1.03, m	1.05, m	1.14, dq (13.1, 3.5)
3	1.51, m	1.50, m	1.60, m	1.39, m
4a	1.74, m	1.74, m	1.84, m	2.73, dd (9.9, 9.9)
4b	0.79, q (12.3)	0.79, q (12.4)	0.82, m	
5	1.81, m	1.81, m	1.82, m	1.77, m
6	5.39, brd (9.8)	5.40,brd (9.8)	5.42, brd (9.8)	6.00, brd (10.1)
7	5.59, ddd (9.8, 4.3, 2.7)	5.59, ddd (9.8, 4.4, 2.7)	5.60, ddd (9.8, 4.4, 2.5)	5.68, ddd (10.1, 4.4, 2.6)
8	2.82, m	2.81, m	2.83, m	2.81, m
9	4.41, dd (11.3, 5.8)	4.41, dd (11.1, 5.6)	4.43, dd (11.3, 5.7)	4.46, dd (11.2, 5.8)
10	1.55, m	1.55, m	1.58, m	1.71, m
11	0.92, d (6.5)	0.92, d (6.5)	3.39, dd (6.0, 1.2)	1.03, d (6.4)
12	0.81, d (7.2)	0.81, d (7.2)	0.82, d (7.2)	0.82, d (7.2)
17	8.00, s	7.77, s	7.57, s	7.56, s
21	3.87, d (7.9)	4.20, s	3.63, d (3.6)	3.63, d (3.8)
22	3.35, dd (9.8, 7.9)	3.65. m	3.41, t (3.6)	3.41, dd, (3.8, 2.8)
23	3.69, m	3.73, m	4.12, m	4.12, m
24a	1.91, m	1.98, m	1.81, m	1.81, m
24b	1.31, m	1.55, m	1.34, m	1.33, m
25a	2.82, m	2.59, m	2.20, m	2.20, m
25b	1.48, m	1.76, m	1.69, ddd (14.2, 10.2, 2.8)	1.69, m
OCH_3_	3.63, s	3.18, s		

**Table 4:** Antibacterial activity of **3**–**6** and **9** (IC_50_, μM) ^a^.

Compounds	MS	SA
**3**	11.4	8.97
**4**	>50	42.8
**5**	19.4	8.37
**6**	35.3	14.1
**9**	2.20	1.66

^a^ Ceftriaxone sodium was used as a positive control (IC_50_ < 1.0 μM); MS: *Mycobacterium smegmatis* ATCC607; SA: *Staphylococcus aureus* ATCC25923.

## Supplementary Materials

The following are available online http://www.mdpi.com/1660-3397/16/5/174/s1. Table S1: ^1^H NMR data for Compounds **1**–**3** (DMSO-*d_6_*) and **10r** (CD_3_OD), Table S2: ^13^C NMR data for Compounds **1**–**3** in DMSO-*d_6_*, Figures: The ECD spectra of Compounds **1**–**2** and 1D/2D NMR and ESIMS spectra of Compounds **1**–**8**.
