# Supplementary material for: Bioactive Pyridone Alkaloids from a Deep-Sea-Derived Fungus Arthrinium sp. UJNMF0008
Source: Mar Drugs. 2018 May 22;16(5):174. doi: 10.3390/md16050174 (PMC5983305; doi:10.3390/md16050174)

Supplementary Information for

Bioactive pyridone alkaloids from a deep-sea derived fungus *Arthrinium* sp. UJNMF0008

Jie Bao[a], Huijuan Zhai[a], Kongkai Zhu[a], Jin-Hai Yu[a], Yuying Zhang[a], Yinyin Wang[a], Cheng-Shi Jiang[a], Xiaoyong Zhang[b], Yun Zhang[c], Hua Zhang[a,]*

[a] *School of Biological Science and Technology, University of Jinan, 336 West Road of Nan Xinzhuang, Jinan 250022, China*

[b] *College of Marine Sciences, South China Agricultural University, 483 Wushan Road, Guangzhou 510642, China*

[c] *Key Laboratory of Tropical Marine Bio-resources and Ecology, South China sea Institute of Oceanology, Chinese Academy of Sciences, 164 West Xingang Road, Guangzhou 510301, China*

List of supporting information

Table S1. ^{1}H NMR data for compounds **1-3** (DMSO-d_6) and **10r (**CD_3OD**)** **P4**

Table S2. ^{13}C NMR data for compounds **1-3** in DMSO-d_6 ....**P5**

Figure S0. The ECD spectra of compounds **1-2**...................... .... **P5**

Figure S1. The ^{1}H-NMR spectrum of arthpyrone D **(1)** in CD_3OD..**P6**

Figure S2. The ^{13}C-NMR spectrum of arthpyrone D **(1)** in CD_3OD...**P6**

Figure S3. The HSQC spectrum of arthpyrone D **(1)** in CD_3OD...**P7**

Figure S4. The ^{1}H-^{1}H COSY spectrum of arthpyrone D in **(1)** CD_3OD.......................................**P7**

Figure S5. The HMBC spectrum of arthpyrone D **(1)** in CD_3OD..**P8**

Figure S6. The NOESY spectrum of arthpyrone D **(1)** in CD_3OD..**P8**

Figure S7. The ^{1}H-NMR spectrum of arthpyrone D **(1)** in DMSO-d_6...**P9**

Figure S8. The ^{13}C-NMR spectrum of arthpyrone D **(1)** in DMSO-d_6..**P9**

Figure S9. The HSQC spectrum of arthpyrone D **(1)** in DMSO-d_6...**P10**

Figure S10. The ^{1}H-^{1}H COSY spectrum of arthpyrone D **(1)** in DMSO-d_6................................**P10**

Figure S11. The HMBC spectrum of arthpyrone D **(1)** in DMSO-d_6..**P11**

Figure S12. The NOESY spectrum of arthpyrone D **(1)** in DMSO-d_6..**P11**

Figure S13. The (+)-HRESIMS spectrum of arthpyrone D **(1)** **P12**

Figure S14. The ^{1}H-NMR spectrum of arthpyrone E **(2)** in CD_3OD..**P12**

Figure S15. The ^{13}C-NMR spectrum of arthpyrone E **(2)** in CD_3OD..**P13**

Figure S16. The HSQC spectrum of arthpyrone E **(2)** in CD_3OD..**P13**
Figure S17. The ^1H-^1H COSY spectrum of arthpyrone E **(2)** in CD_3OD....................................**P14**
Figure S18. The HMBC spectrum of arthpyrone E **(2)** in CD_3OD...**P14**
Figure S19. The NOESY spectrum of arthpyrone E **(2)** in CD_3OD..**P15**
Figure S20. The ^1H-NMR spectrum of arthpyrone E **(2)** in DMSO-d_6.......................................**P15**
Figure S21. The ^{13}C-NMR spectrum of arthpyrone E **(2)** in DMSO-d_6......................................**P16**
Figure S22. The HSQC spectrum of arthpyrone E **(2)** in DMSO-d_6..**P16**
Figure S23. The ^1H-^1H COSY spectrum of arthpyrone E **(2)** in DMSO-d_6................................**P17**
Figure S24. The NOESY spectrum of arthpyrone E **(2)** in DMSO-d_6..**P17**
Figure S25. The (-)-HRESIMS spectrum of arthpyrone E **(2)** **P18**
Figure S26. The ^1H-NMR spectrum of arthpyrone F **(3)** in CD_3OD..**P18**
Figure S27. The ^{13}C-NMR spectrum of arthpyrone F **(3)** in CD_3OD...**P19**
Figure S28. The HSQC spectrum of arthpyrone F **(3)** in CD_3OD..**P19**
Figure S29. The ^1H-^1H COSY spectrum of arthpyrone F **(3)** in CD_3OD....................................**P20**
Figure S30. The HMBC spectrum of arthpyrone F **(3)** in CD_3OD...**P20**
Figure S31. The NOESY spectrum of arthpyrone F **(3)** in CD_3OD...**P21**
Figure S32. The ^1H-NMR spectrum of arthpyrone F **(3)** in DMSO-d_6.......................................**P21**
Figure S33. The ^{13}C-NMR spectrum of arthpyrone F **(3)** in DMSO-d_6......................................**P22**
Figure S34. The HSQC spectrum of arthpyrone F **(3)** in DMSO-d_6...**P22**
Figure S35. The ^1H-^1H COSY spectrum of arthpyrone F **(3)** in DMSO-d_6.................................**P23**
Figure S36. The HMBC spectrum of arthpyrone F **(3)** in DMSO-d_6...**P23**
Figure S37. The NOESY spectrum of arthpyrone F **(3)** in DMSO-d_6..**P24**
Figure S38. The (-)-HRESIMS spectrum of arthpyrone F **(3)** **P24**
Figure S39. The ^1H-NMR spectrum of arthpyrone G **(4)** in CD_3OD...**P25**
Figure S40. The ^{13}C-NMR spectrum of arthpyrone G **(4)** in CD_3OD..**P25**
Figure S41. The HSQC spectrum of arthpyrone G **(4)** in CD_3OD...**P26**
Figure S42. The ^1H-^1H COSY spectrum of arthpyrone G **(4)** inCD_3OD....................................**P26**
Figure S43. The HMBC spectrum of arthpyrone G **(4)** in CD_3OD..**P27**
Figure S44. The NOESY spectrum of arthpyrone G **(4)** in CD_3OD..**P27**
Figure S45. The (-)-HRESIMS spectrum of arthpyrone G **(4)** **P28**
Figure S46. The ^1H-NMR spectrum of arthpyrone H **(5)** in CD_3OD..**P28**
Figure S47. The ^{13}C-NMR spectrum of arthpyrone H **(5)** in CD_3OD..**P29**
Figure S48. The DEPT 135 spectrum of arthpyrone H **(5)** in CD_3OD...**P29**
Figure S49. The HSQC spectrum of arthpyrone H **(5)** in CD_3OD...**P30**
Figure S50. The ^1H-^1H COSY spectrum of arthpyrone H **(5)** inCD_3OD....................................**P30**
Figure S51. The HMBC spectrum of arthpyrone H **(5)** in CD_3OD..**P31**
Figure S52. The NOESY spectrum of arthpyrone H **(5)** in CD_3OD..**P31**

Figure S53. The (-)-HRESIMS spectrum of arthpyrone H **(5)** **P32**
Figure S54. The ^{1}H-NMR spectrum of arthpyrone I **(6)** in CD_3OD..**P32**
Figure S55. The ^{13}C-NMR spectrum of arthpyrone I **(6)** in CD_3OD...**P33**
Figure S56. The HSQC spectrum of arthpyrone I **(6)** in CD_3OD..**P33**
Figure S57. The ^{1}H-^{1}H COSY spectrum of arthpyrone I **(6)** inCD_3OD......................................**P34**
Figure S58. The HMBC spectrum of arthpyrone I **(6)** in CD_3OD...**P34**
Figure S59. The NOESY spectrum of arthpyrone I **(6)** in CD_3OD...**P35**
Figure S60. The (-)-HRESIMS spectrum of arthpyrone I **(6)** **P35**
Figure S61. The ^{1}H-NMR spectrum of arthpyrone J **(7)** in CD_3OD...**P36**
Figure S62. The ^{13}C-NMR of arthpyrone J **(7)** in CD_3OD..**P36**
Figure S63. The HSQC spectrum of arthpyrone J **(7)** in CD_3OD..**P37**
Figure S64. The ^{1}H-^{1}H COSY spectrum of arthpyrone J **(7)** in CD_3OD.....................................**P37**
Figure S65. The HMBC spectrum of arthpyrone J **(7)** in CD_3OD...**P38**
Figure S66. The NOESY spectrum of arthpyrone J **(7)** in CD_3OD..**P38**
Figure S67. The (-)-HRESIMS spectrum of arthpyrone J **(7)** **P39**
Figure S68. The ^{1}H-NMR spectrum of arthpyrone K **(8)** in CD_3OD...**P39**
Figure S69. The ^{13}C-NMR spectrum of arthpyrone K **(8)** in CD_3OD..**P40**
Figure S70. The HSQC spectrum of arthpyrone K **(8)** in CD_3OD...**P40**
Figure S71. The ^{1}H-^{1}H COSY spectrum of arthpyrone K **(8)** inCD_3OD....................................**P41**
Figure S72. The HMBC spectrum of arthpyrone K **(8)** in CD_3OD...**P41**
Figure S73. The NOESY spectrum of arthpyrone K **(8)** in CD_3OD..**P42**
Figure S74. The (-)-HRESIMS spectrum of arthpyrone K **(8)** **P42**
Figure S75. The ^{1}H-NMR spectrum of **10r** and arthpyrone D **(1)** in CD_3OD **P43**
Figure S76. The (-)-ESIMS spectrum of **10r**…...**P43**

Table S1. ^{1}H NMR data for compounds **1-3** (DMSO-d_6) and **10r** (CD_3OD)

pos.	**1**	**2**	**3**	**10r**
	δ_H, mult. (J in Hz)	δ_H, mult. (J in Hz)	δ_H, mult. (J in Hz)	δ_H, mult. (J in Hz)
1a	2.06, m	2.08, m	1.77,m	2.11, m
1b	0.95, m	0.94, m	0.82, m	1.08, m
2a	1.71, m	1.70, m	1.66, m	1.71-1.86,[a] m
2b	0.95, m	0.95, m	0.96, m	1.01, m
3	1.48, m	1.47, m	1.46, m	1.52, m
4a	1.71, m	1.71, m	1.72, m	1.71-1.86, m
4b	0.73, q (12.6)	0.73, q (12.4)	0.74, m	0.78, q (12.0)
5	1.78, m	1.77, m	1.72, m	1.71-1.86,[a] m
6	5.38, brd (9.9)	5.38, brd (9.9)	5.38,brd (9.8)	5.41, brd (9.9)
7	5.60, ddd (9.9, 4.3, 2.8)	5.59, ddd, (9.9, 4.2, 2.8)	5.58,ddd (9.8, 4.1, 2.6)	5.61, ddd (9.9, 4.5, 2.8)
8	2.62, m	2.63, m	2.74, m	2.76, m
9	2.69, dd (11.6, 5.9)	2.68, dd, (11.5, 6.0)	4.34, dd (11.3, 5.7)	2.91, dd (11.6,5.9)
10	1.29, m	1.29, m	1.46,m	1.39, m
11	0.88, d (6.5)	0.88 d (6.5)	0.87, d (6.5)	0.93, d (6.6)
12	1.04, d (7.0)	1.03, d (7.0)	0.74, d (7.1)	1.11, d (7.1)
17	7.08, s	7.05, s	7.57, d (6.0)	7.37, s
21	3.57, dd (3.1, 2.7)	3.44, d (8.6)	4.90, d (10.0)	3.73, d (2.0)
22	4.39,br s	3.19, dd (8.6, 7.4)	3.68,ddd (10.0, 7.3, 2.9)	4.60, brs
23	3.71, m	3.40, m	3.87, brs	3.83, ddd (11.6, 4.9, 2.3)
24a	1.61, m	1.82, m	1.86, m	1.71-1.86,[a] m
24b	1.11, m	1.25, m	1.52, m	1.35, m
25a	1.66, m	2.18, m	2.67, m	1.71-1.86,[a] m
25b	1.51, m	1.53, m	1.36, m	1.71-1.86,[a] m
NH	11.36, s	11.15, s	11.54, d (5.1)	
20-OH	5.21, s		5.27, s	
21-OH	5.33, d (3.1)			
22-OH			4.90, d, (7.3)	
23-OH			4.58, s	

[a] Overlapped signals.

Table S2. ^{13}C NMR data for compounds **1-3** in DMSO-d_6

pos.	**1**	**2**	**3**
	δ_c, type	δ_c, type	δ_c, type
1	29.1, CH_2	29.2, CH_2	29.4, CH_2
2	34.9, CH_2	34.9, CH_2	35.0, CH_2
3	32.5, CH	32.5, CH	32.5, CH
4	41.1, CH_2	41.1, CH_2	41.3, CH_2
5	41.2, CH	41.2, CH	41.3, CH
6	130.2, CH	130.3, CH	130.4, CH
7	131.2, CH	131.2, CH	131.6, CH
8	32.1, CH	32.1, CH	30.6, CH
9	48.4, CH	48.5, CH	51.6, CH
10	36.0, CH	36.0, CH	35.8, CH
11	22.4, CH_3	22.4, CH_3	22.4, CH_3
12	17.5, CH_3	17.5, CH_3	17.7, CH_3
13	170.1, C	170.4, C	210.0, C
14	121.6, C	124.5, C	106.4, C
15	155.5, C	157.6, C	161.6, C
17	127.8, CH	130.4, CH	140.8, CH
18	112.8, C	112.4, C	115.7, C
19	157.8, C	158.5, C	176.1, C
20	69.0, C	76.3, C	75.0, C
21	68.3, CH	77.7, CH	67.6, CH
22	83.6, CH	74.5, CH	72.0, CH
23	69.4, CH	71.6, CH	69.4, CH
24	26.5, CH_2	28.2, CH_2	26.2, CH_2
25	36.6, CH_2	32.5, CH_2	30.0, CH_2

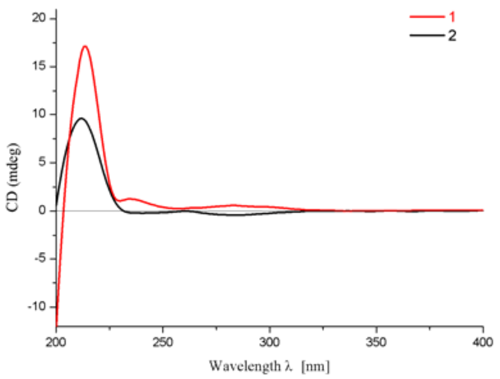

Figure S0. The ECD spectra of compounds **1-2**

Figure S1. The ^{1}H-NMR spectrum of arthpyrone D **(1)** in CD_3OD

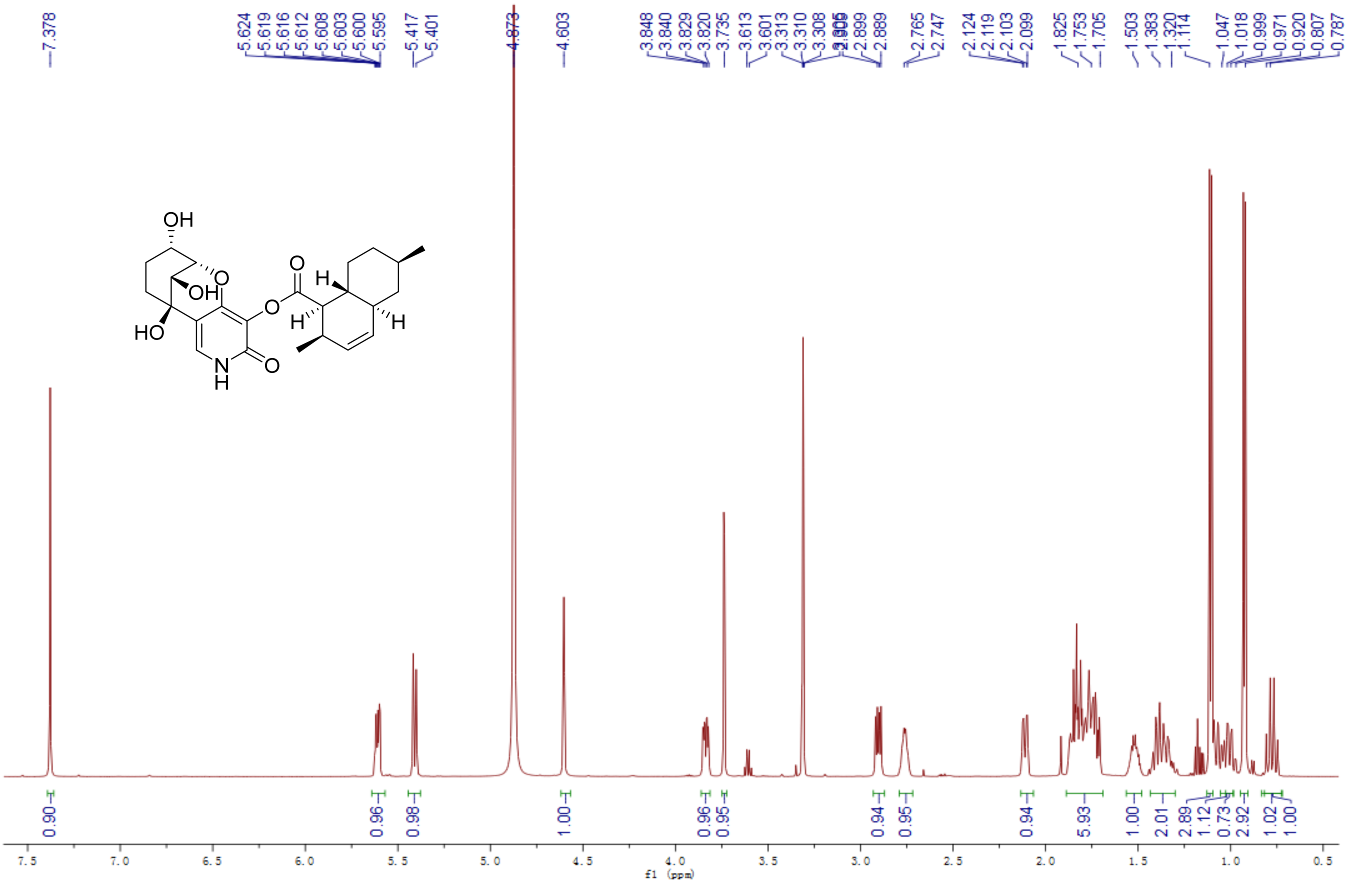

Figure S2. The ^{13}C-NMR spectrum of arthpyrone D **(1)** in CD_3OD

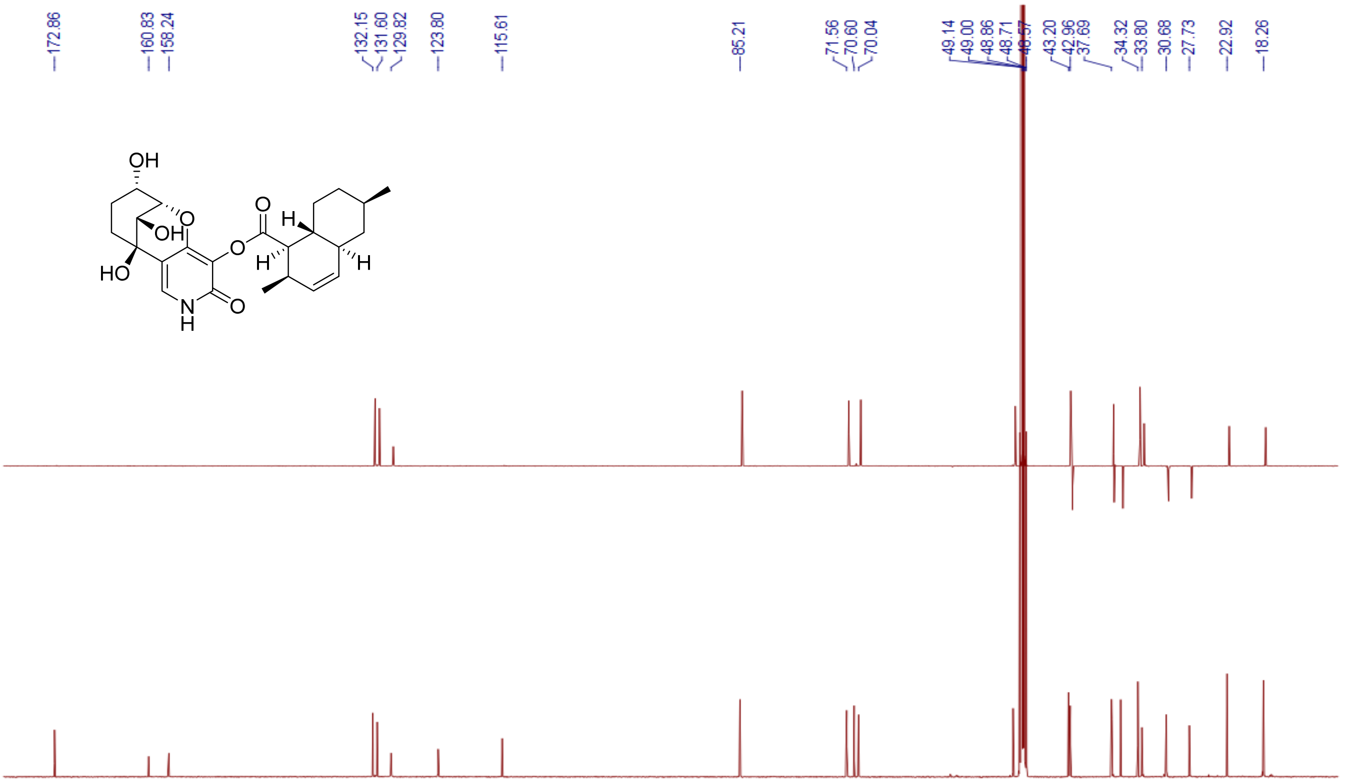

Figure S3. The HSQC spectrum of arthpyrone D **(1)** in CD_3OD

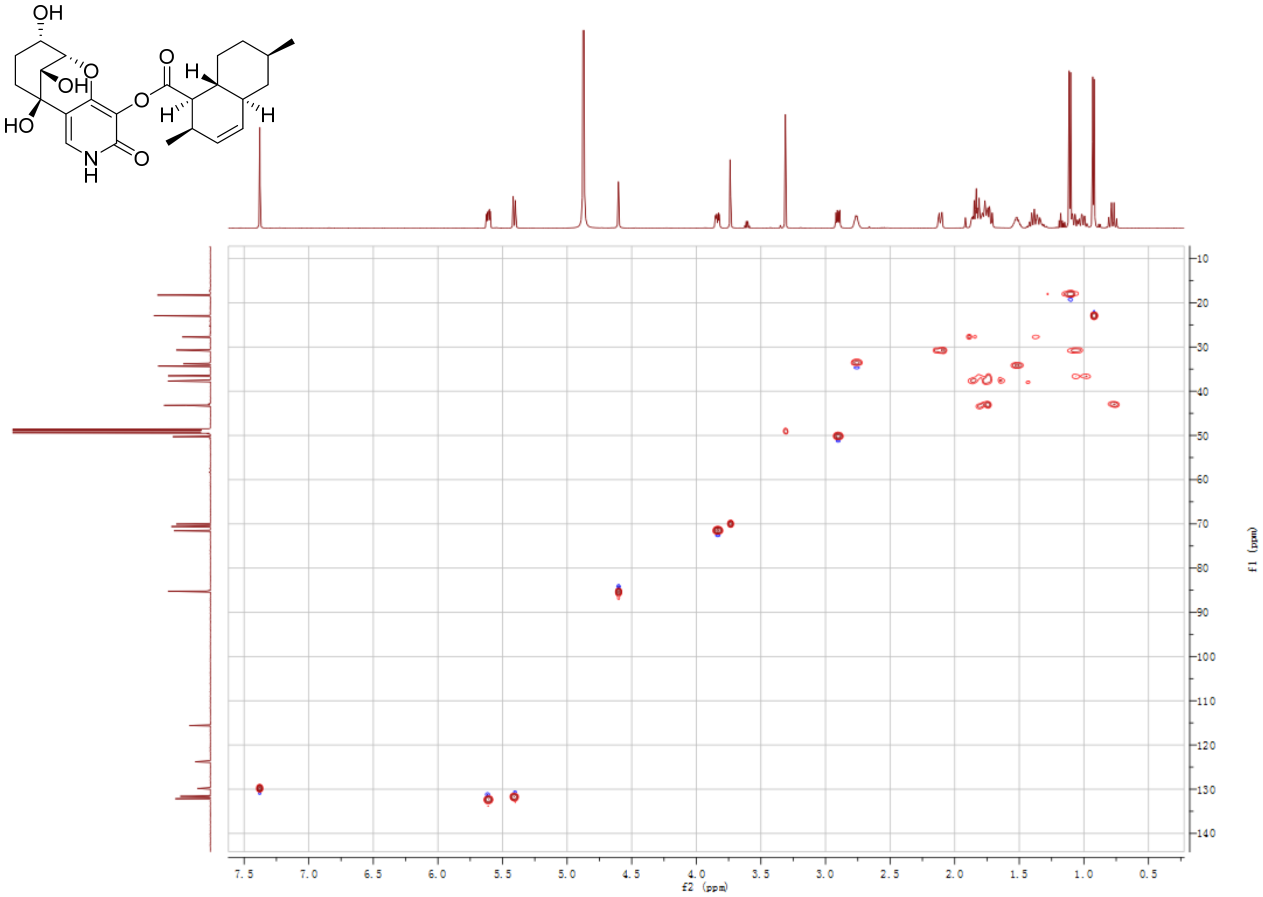

Figure S4. The ^1H-^1H COSY spectrum of arthpyrone D in **(1)** CD_3OD

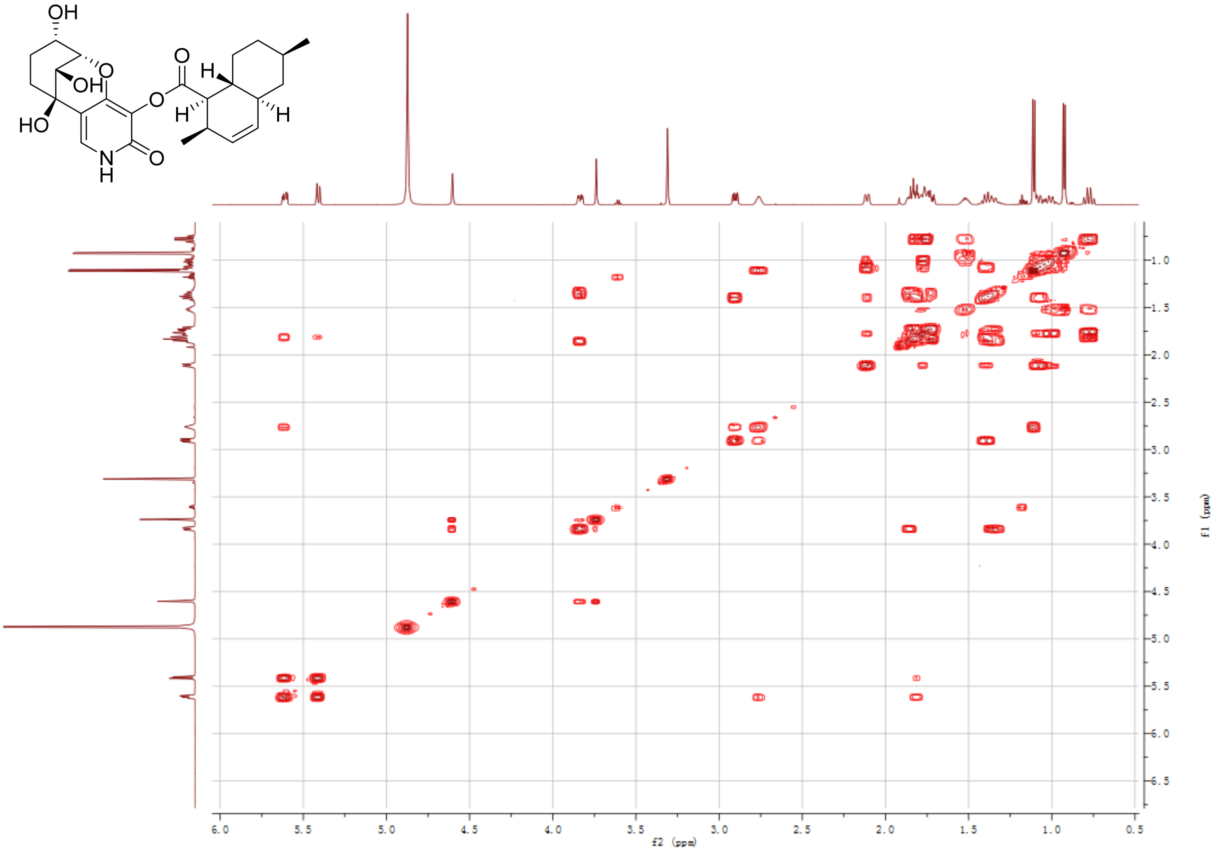

Figure S5. The HMBC spectrum of arthpyrone D **(1)** in CD_3OD

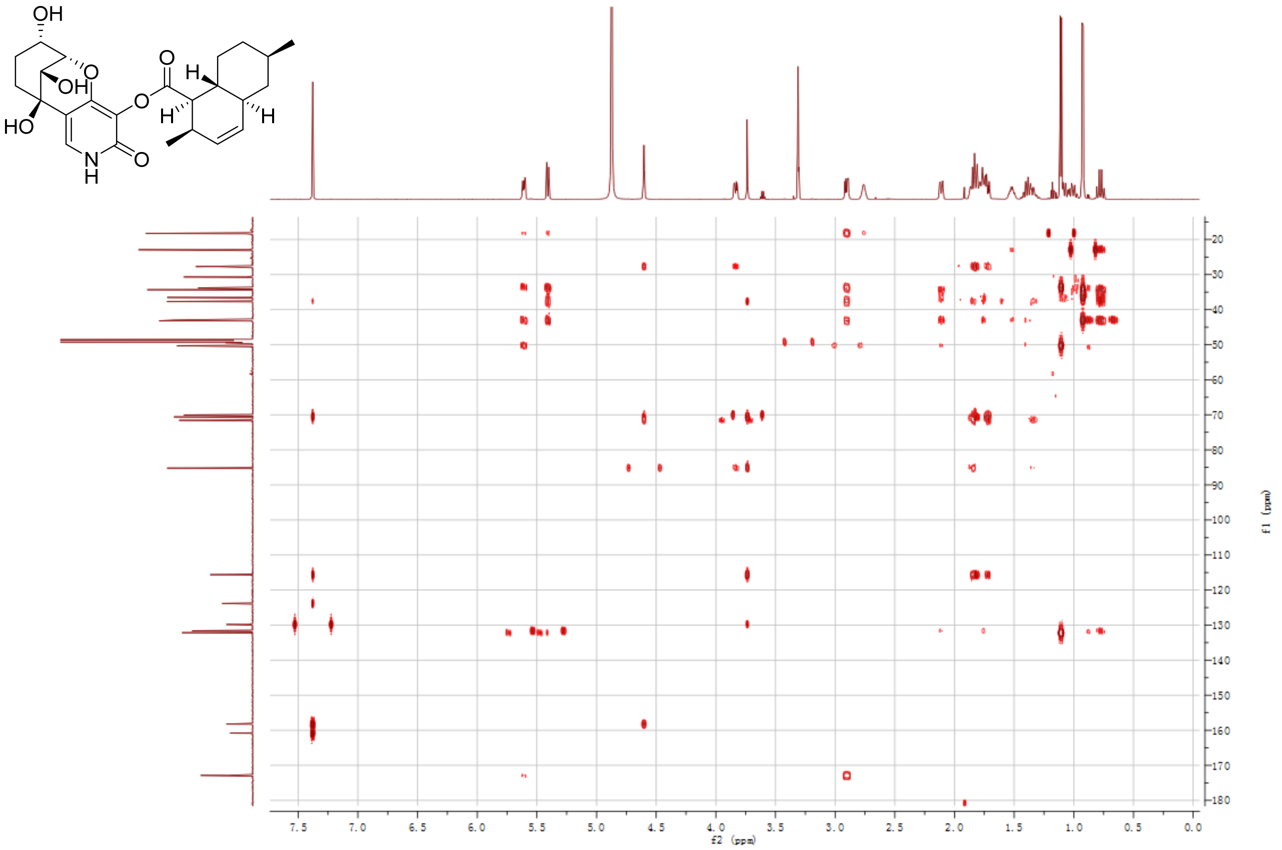

Figure S6. The NOESY spectrum of arthpyrone D **(1)** in CD_3OD

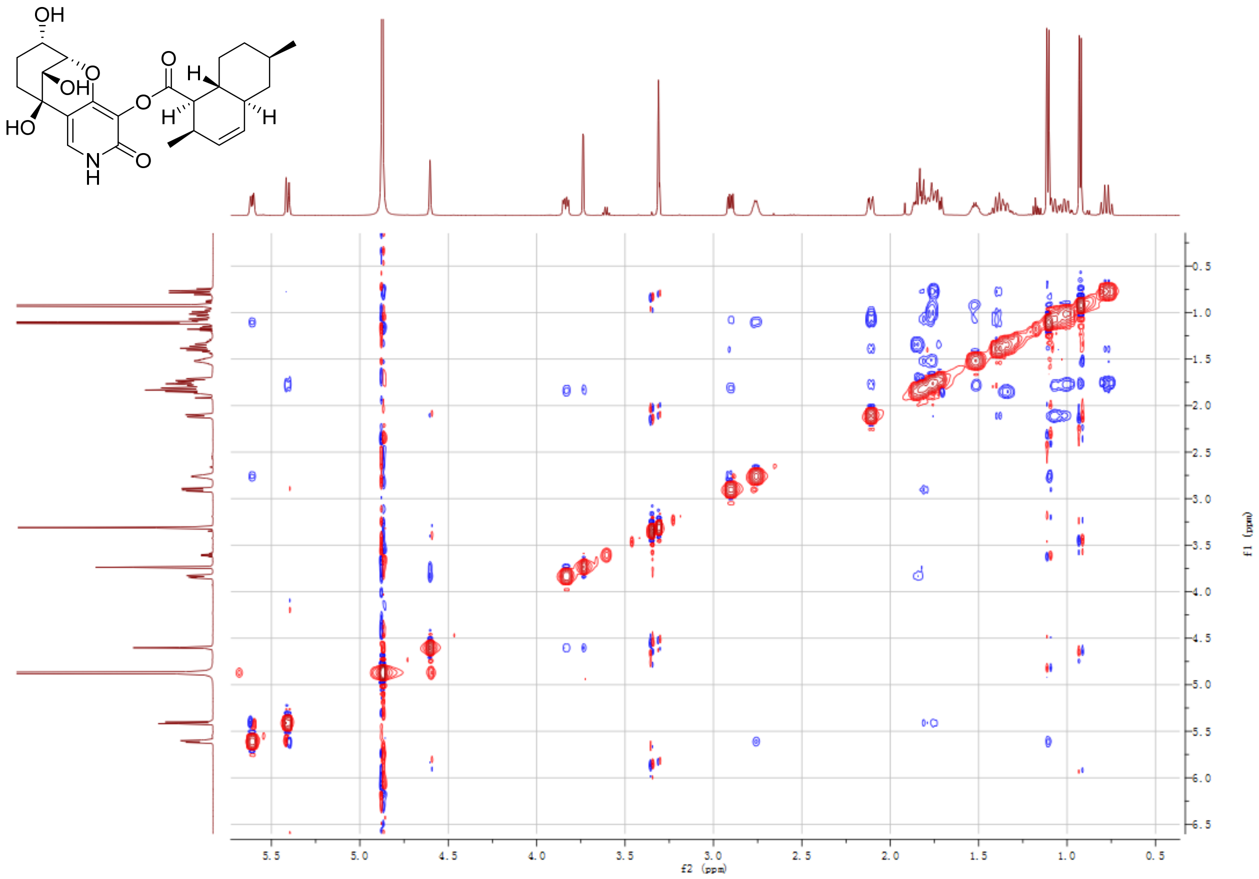

Figure S7. The ^{1}H-NMR spectrum of arthpyrone D **(1)** in DMSO-d_6

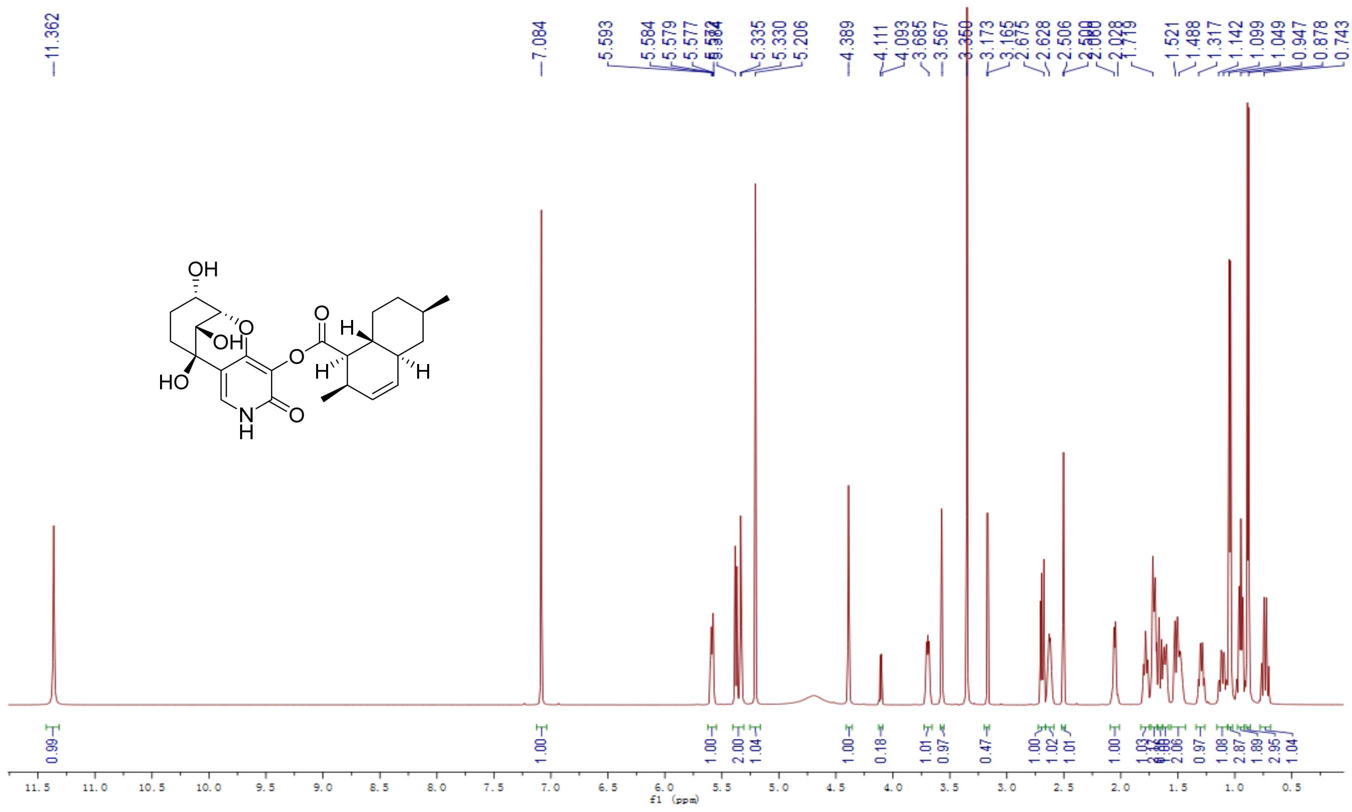

Figure S8. The ^{13}C-NMR spectrum of arthpyrone D **(1)** in DMSO-d_6

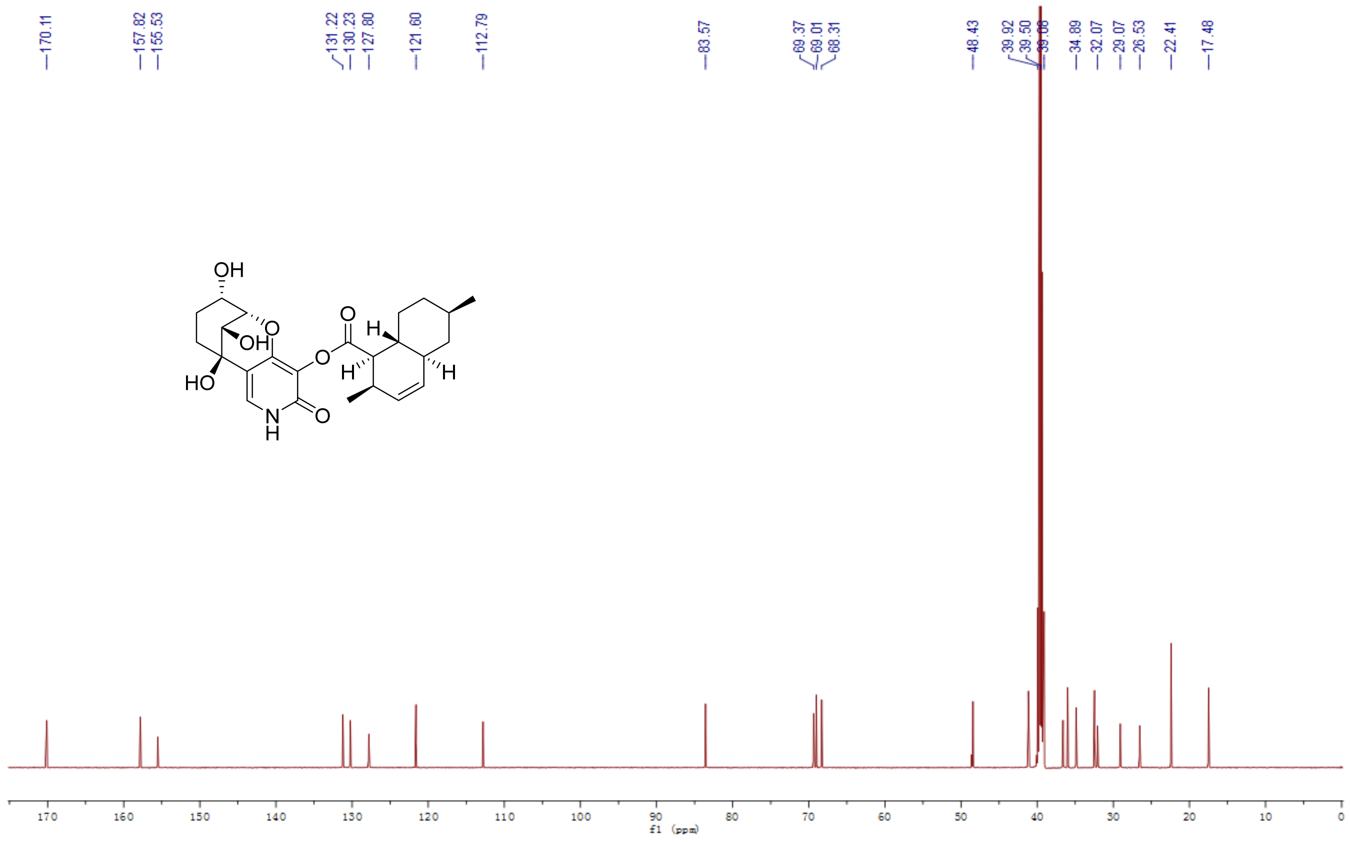

Figure S9. The HSQC spectrum of arthpyrone D **(1)** in DMSO-d_6

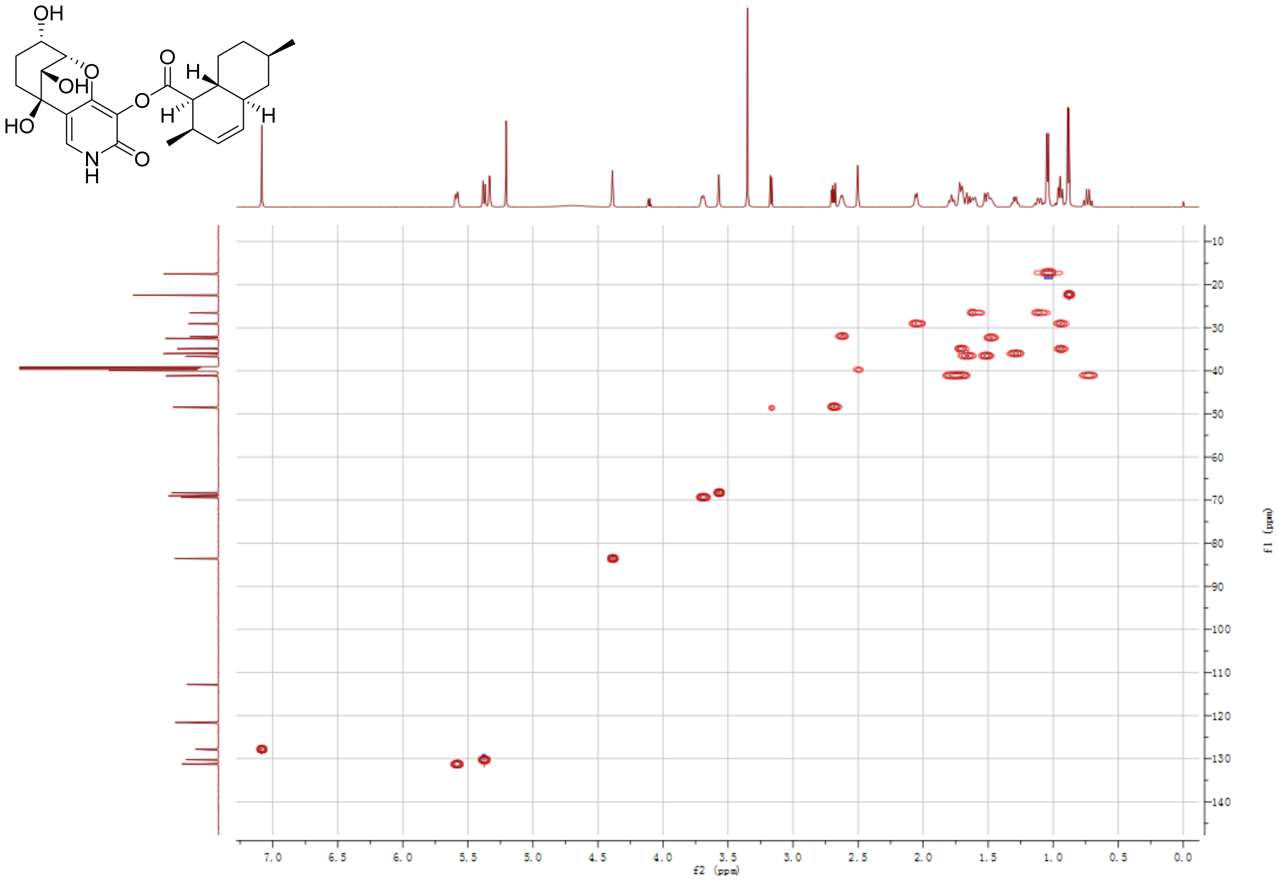

Figure S10. The ^{1}H-^{1}H COSY spectrum of arthpyrone D **(1)** in DMSO-d_6

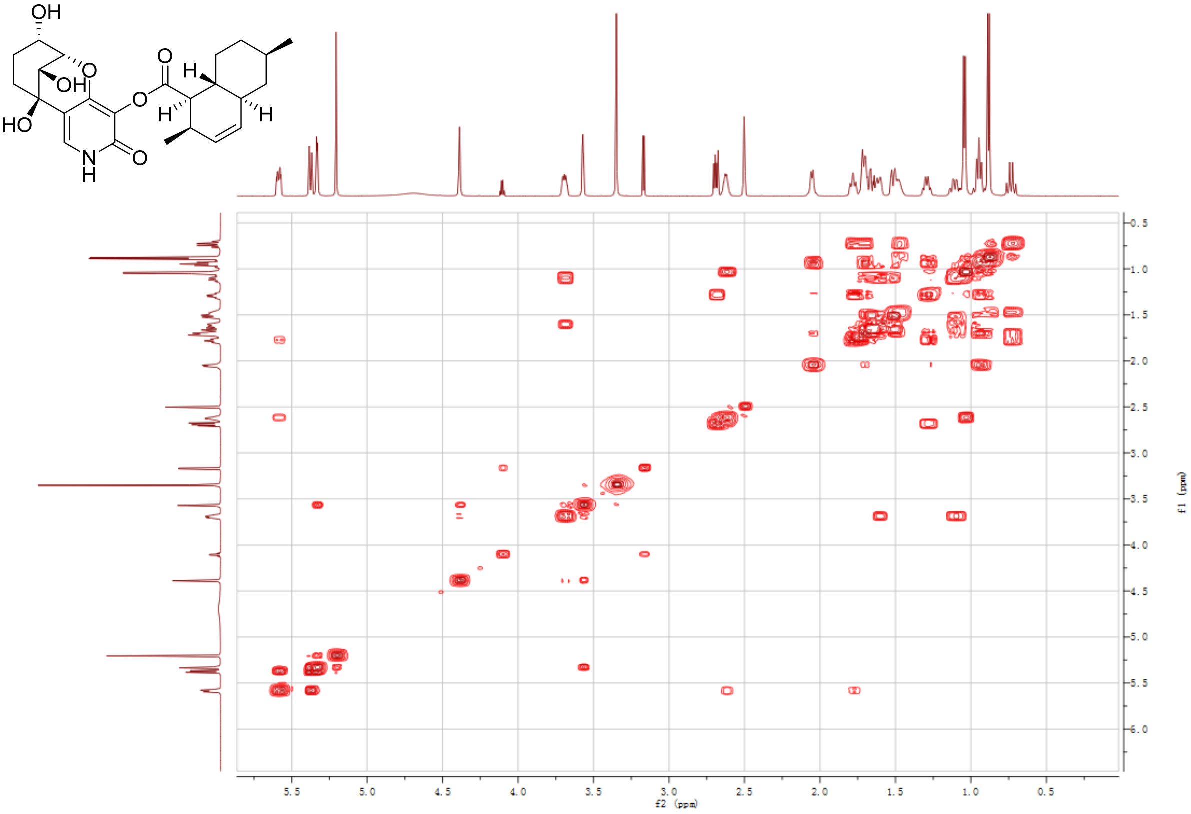

Figure S11. The HMBC spectrum of arthpyrone D **(1)** in DMSO-d_6

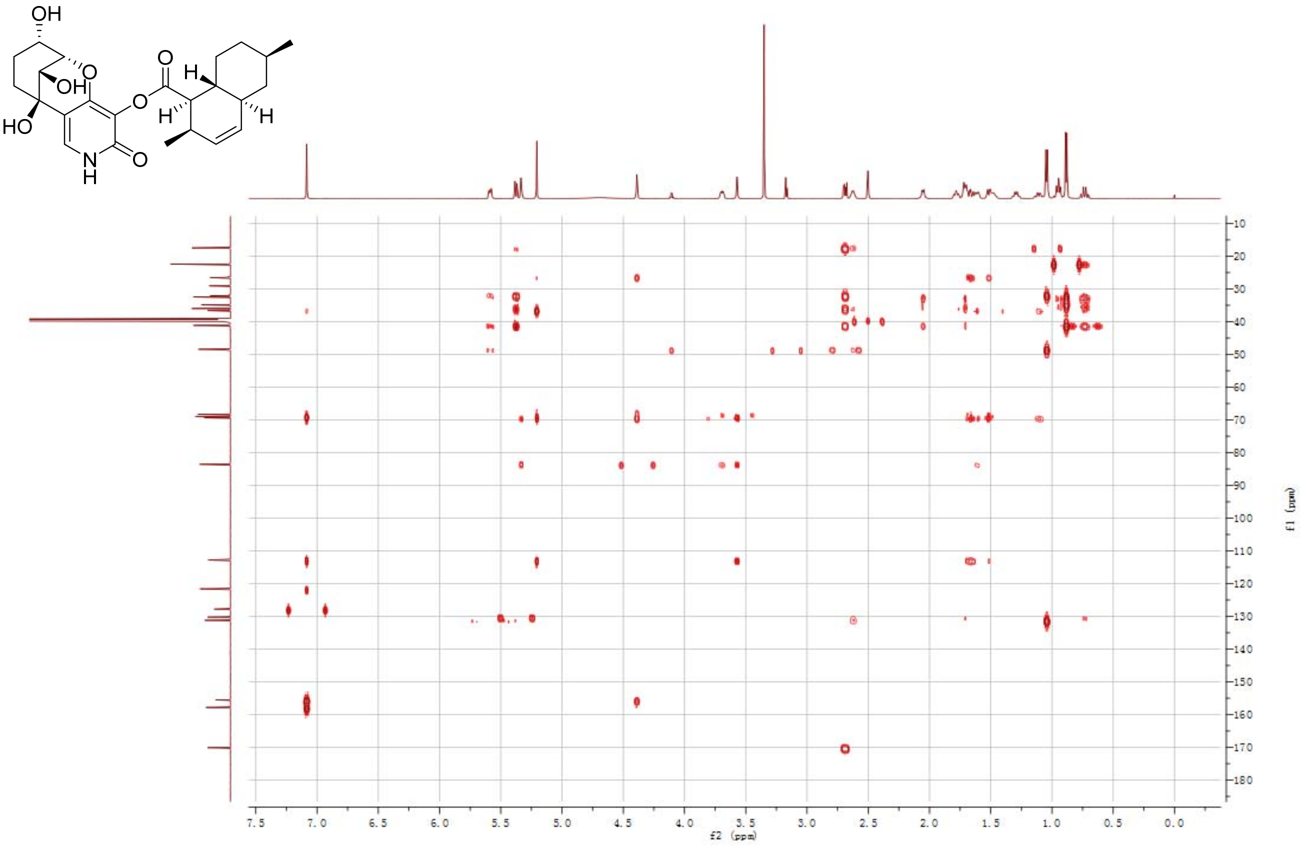

Figure S12. The NOESY spectrum of arthpyrone D **(1)** DMSO-d_6

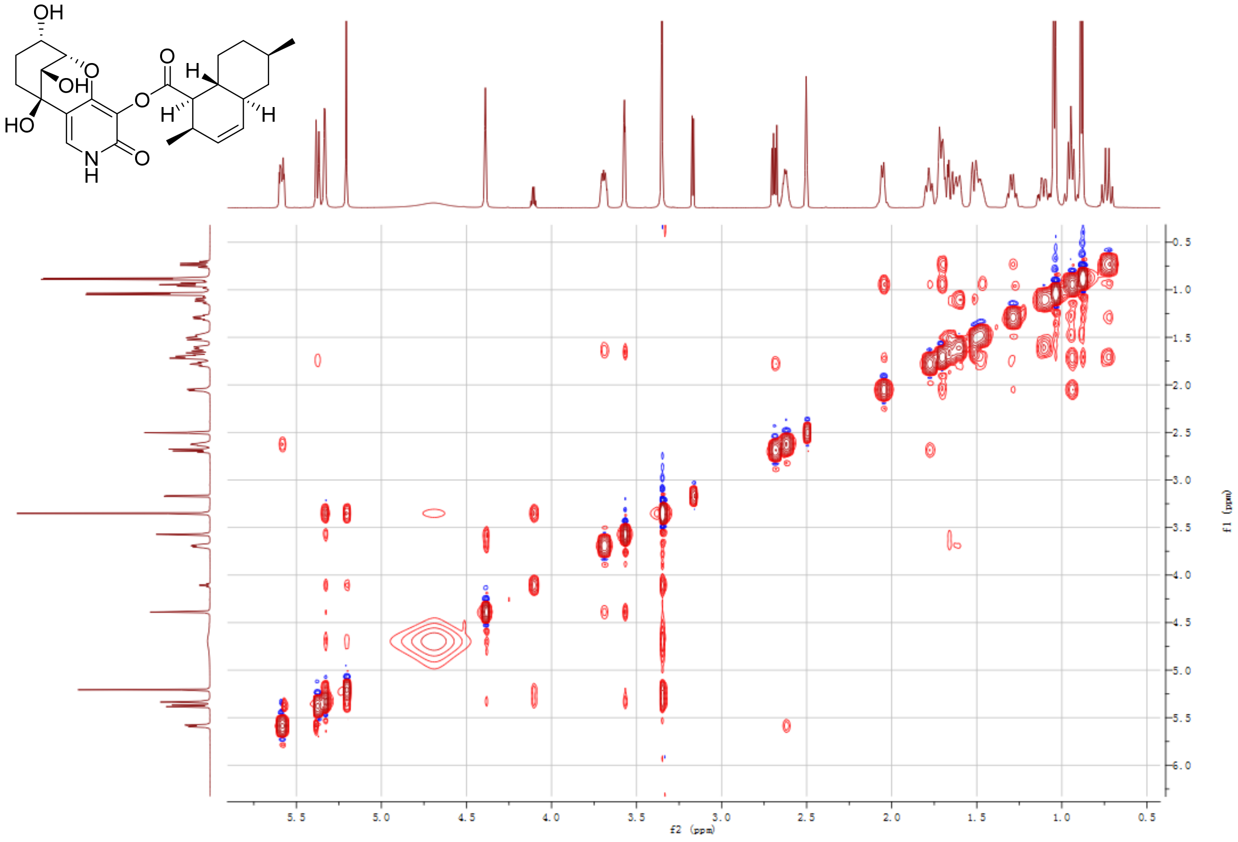

Figure S13. The (+)-HRESIMS spectrum of arthpyrone D **(1)**

**Sample Name**	BJSH8-37A	**Position**	P1-C1	**Instrument Name**	Instrument 1
**User Name**		**Inj Vol**	1	**InjPosition**	
**Sample Type**	Sample	**IRM Calibration Status**	Some Ions Missed	**Data Filename**	BJSH8-37A-.d
**ACQ Method**	wss-isocratic elution-Negative.m	**Comment**		**Acquired Time**	1/11/2018 12:09:49 PM

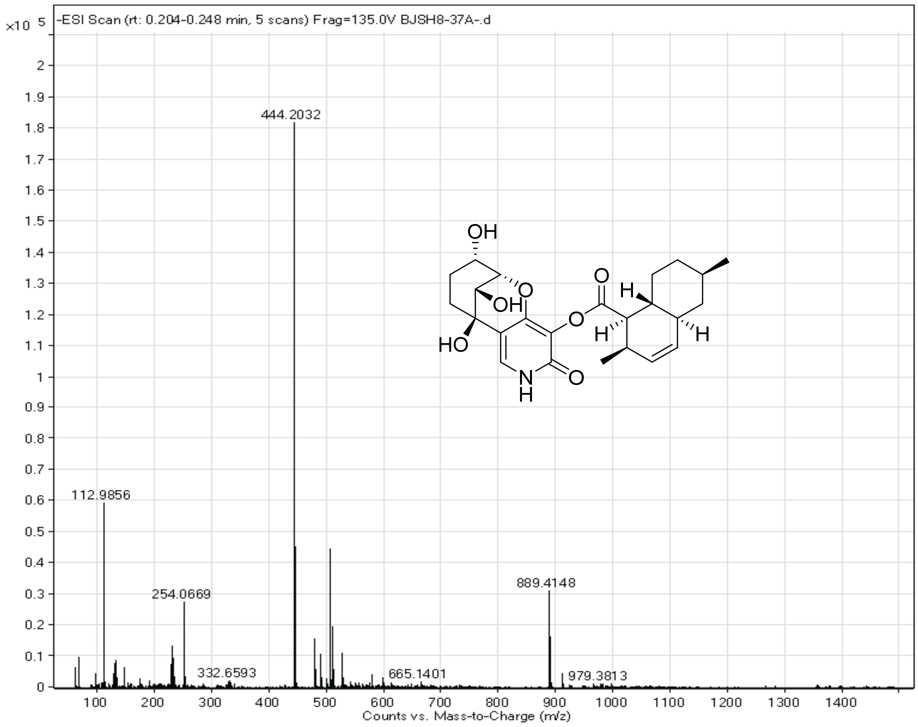

Figure S14. The ^{1}H-NMR spectrum of arthpyrone E **(2)** in CD_3OD

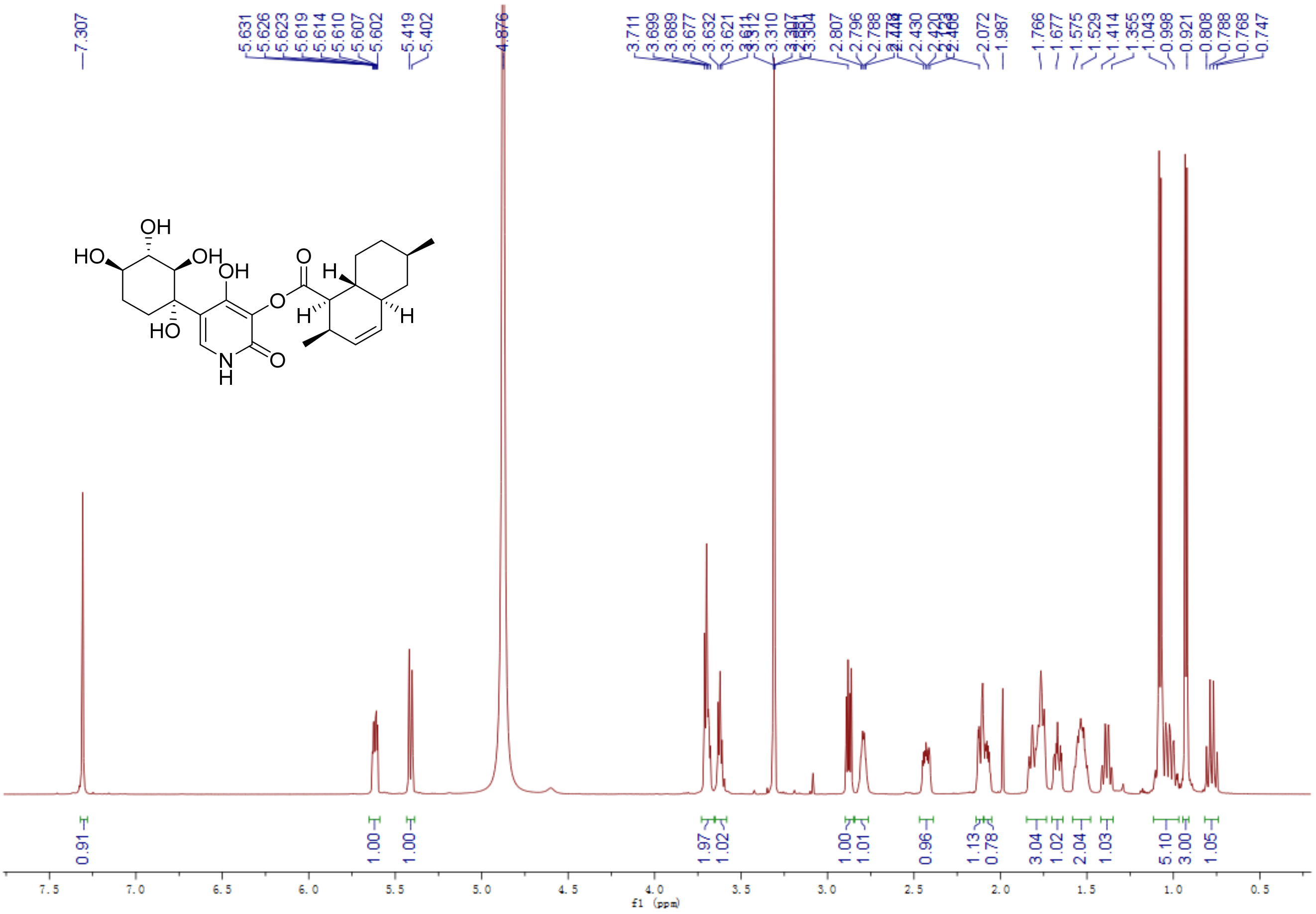

Figure S15. The ^{13}C-NMR and DEPT 135 spectrum of arthpyrone E **(2)** in CD_3OD

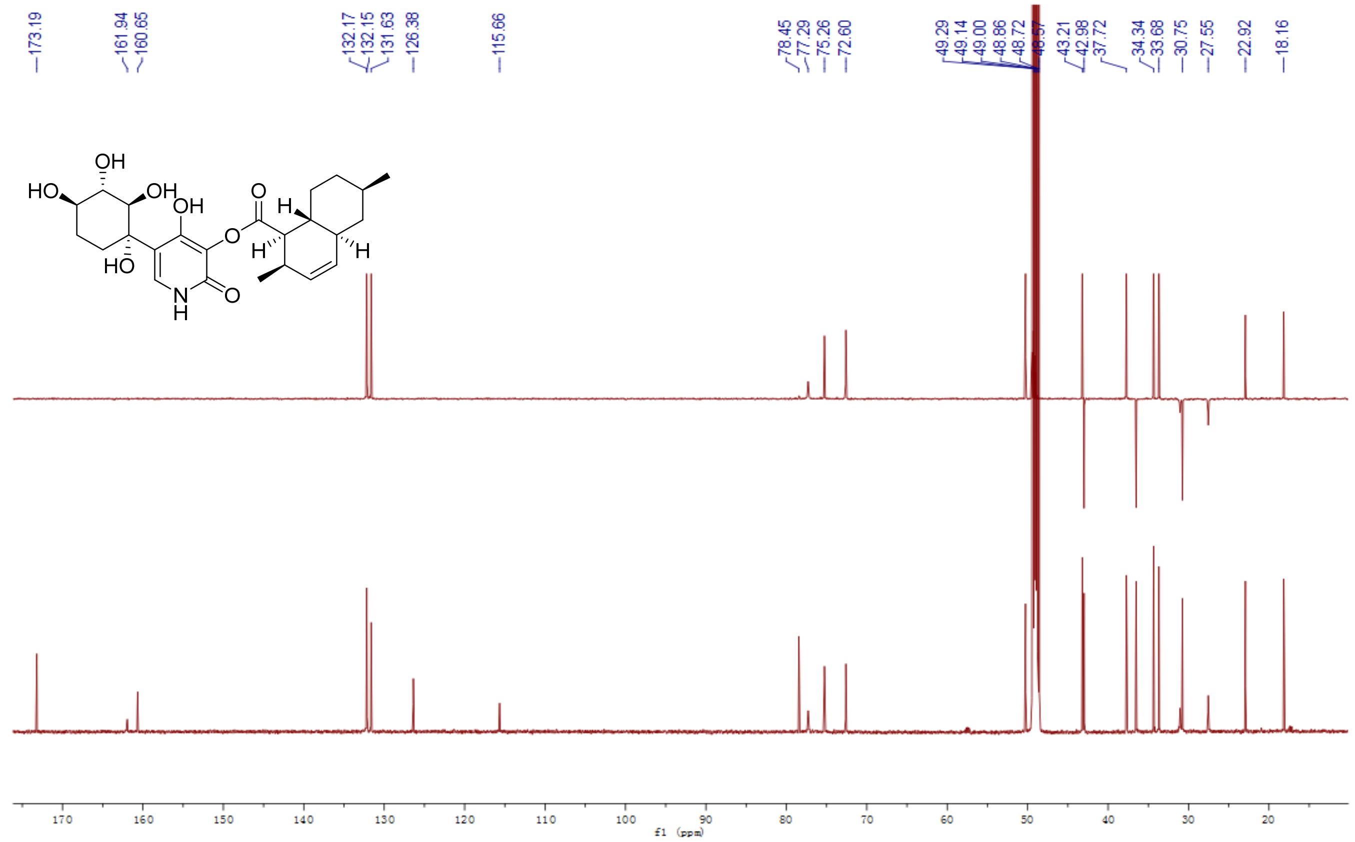

Figure S16. The HSQC spectrum of arthpyrone E **(2)** in CD_3OD

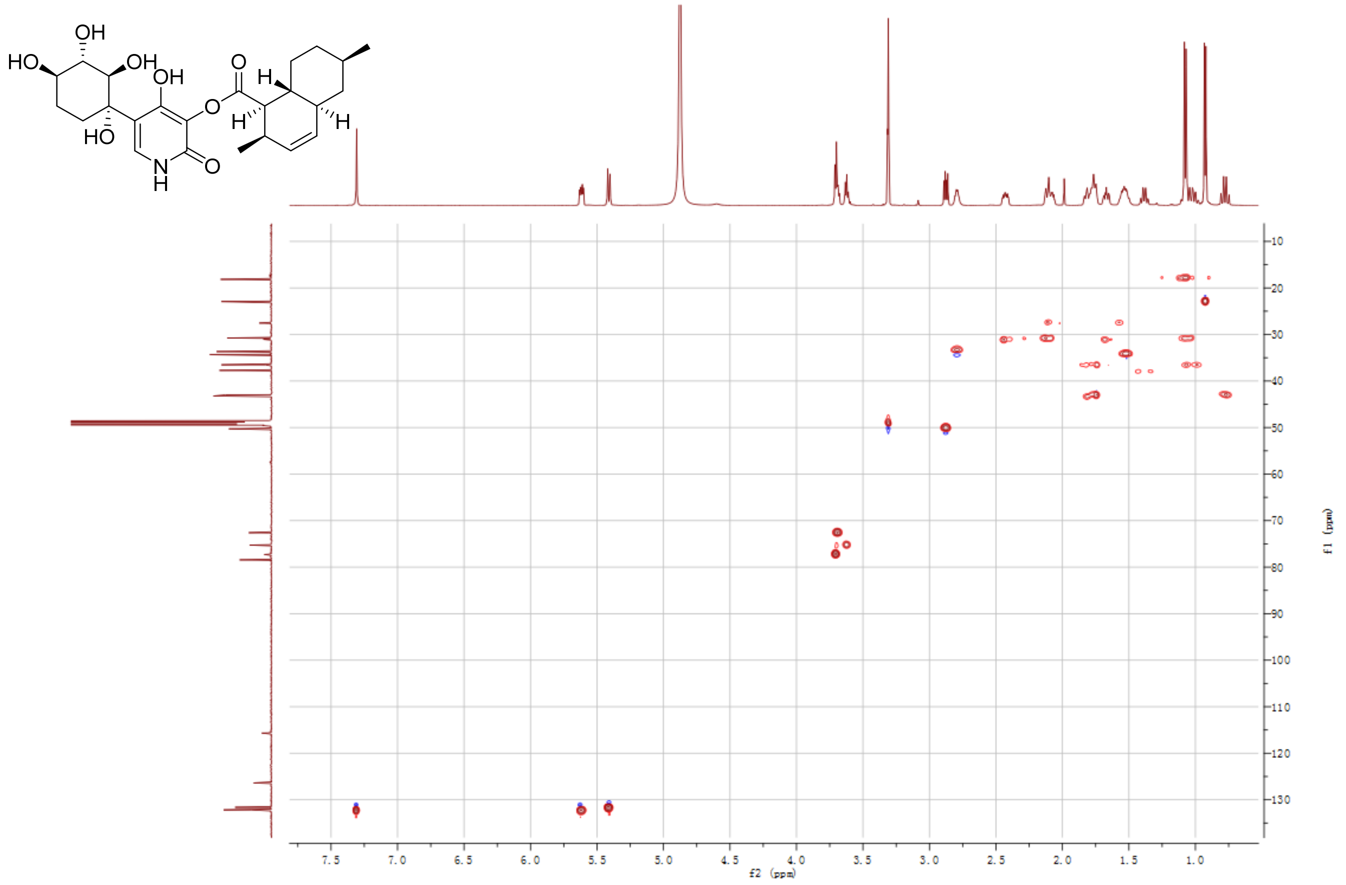

Figure S17. The ^{1}H-^{1}H COSY spectrum of arthpyrone E in **(2)** CD_3OD

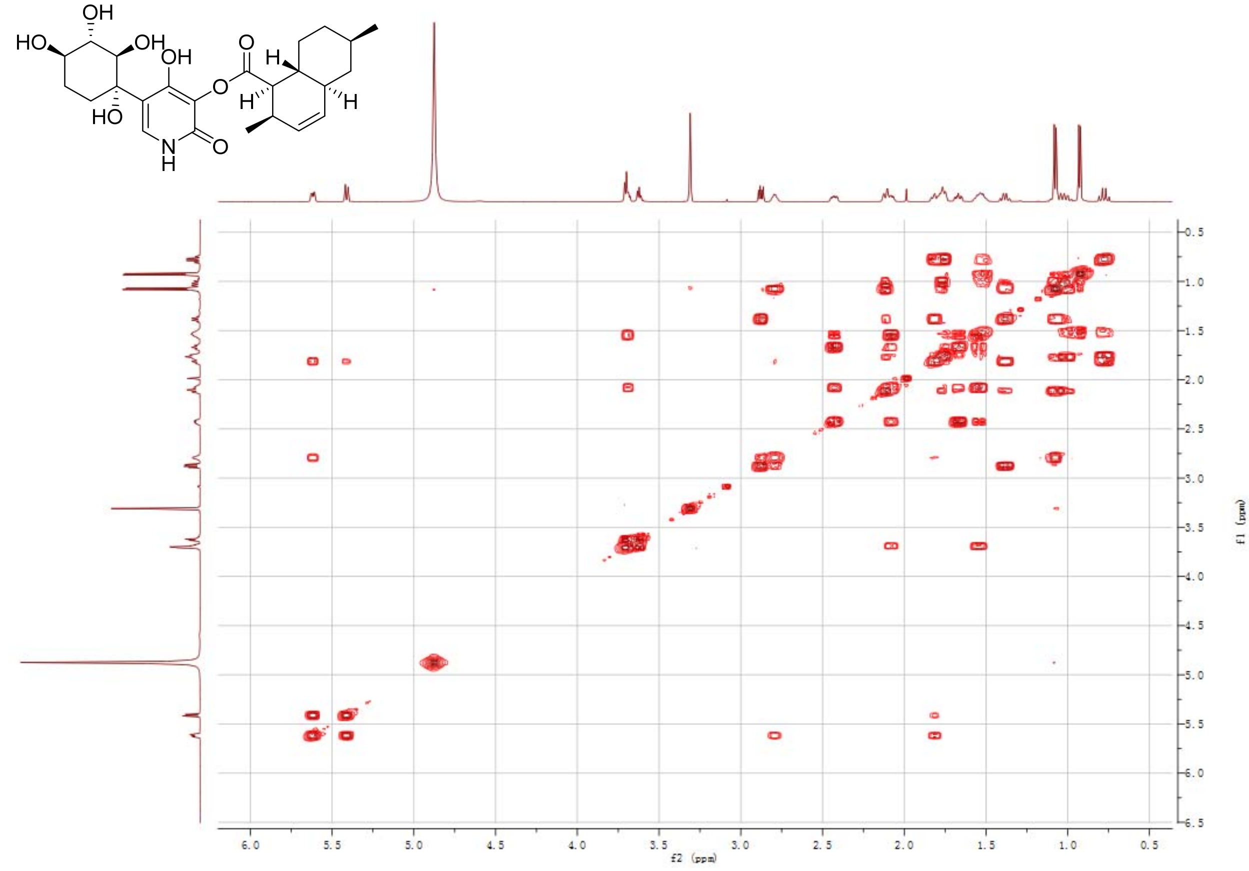

Figure S18. The HMBC spectrum of arthpyrone E **(2)** in CD_3OD

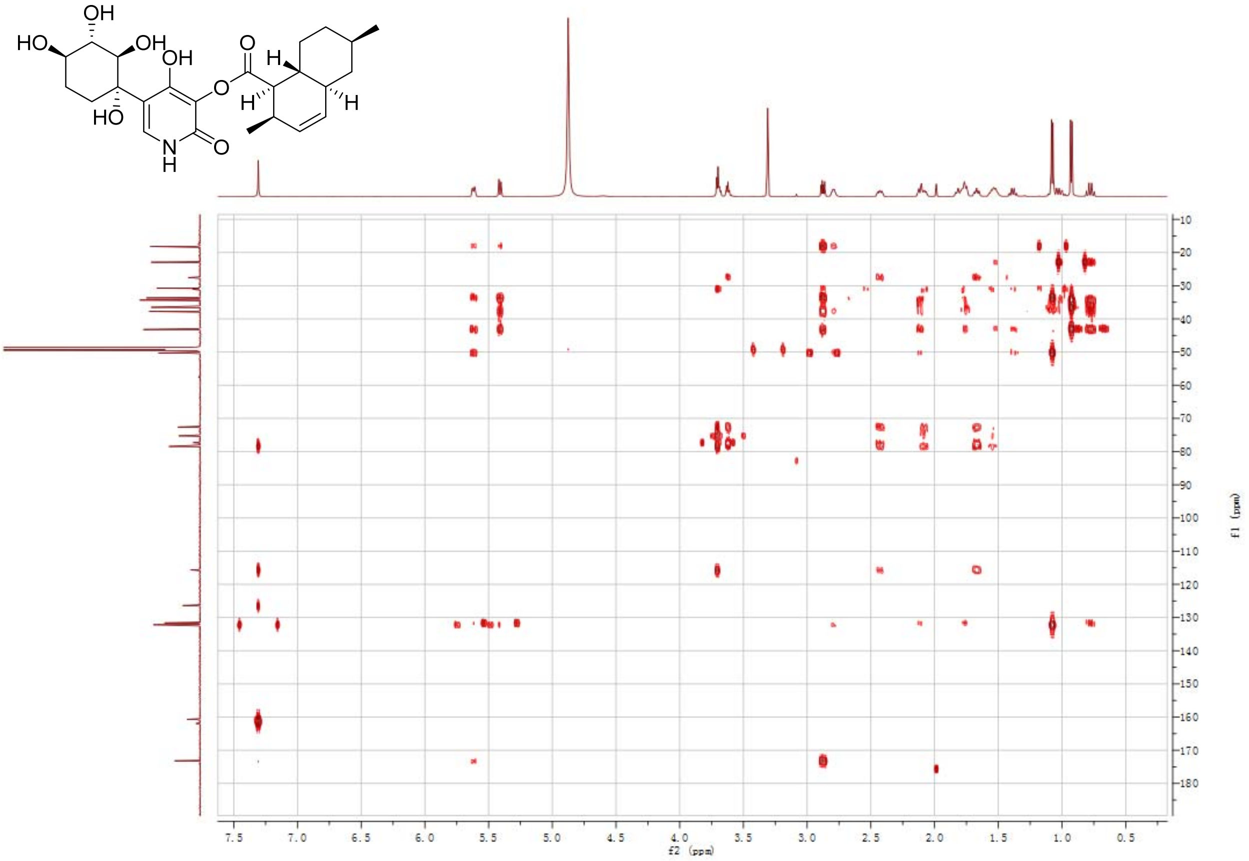

Figure S19. The NOESY spectrum of arthpyrone E **(2)** in CD_3OD

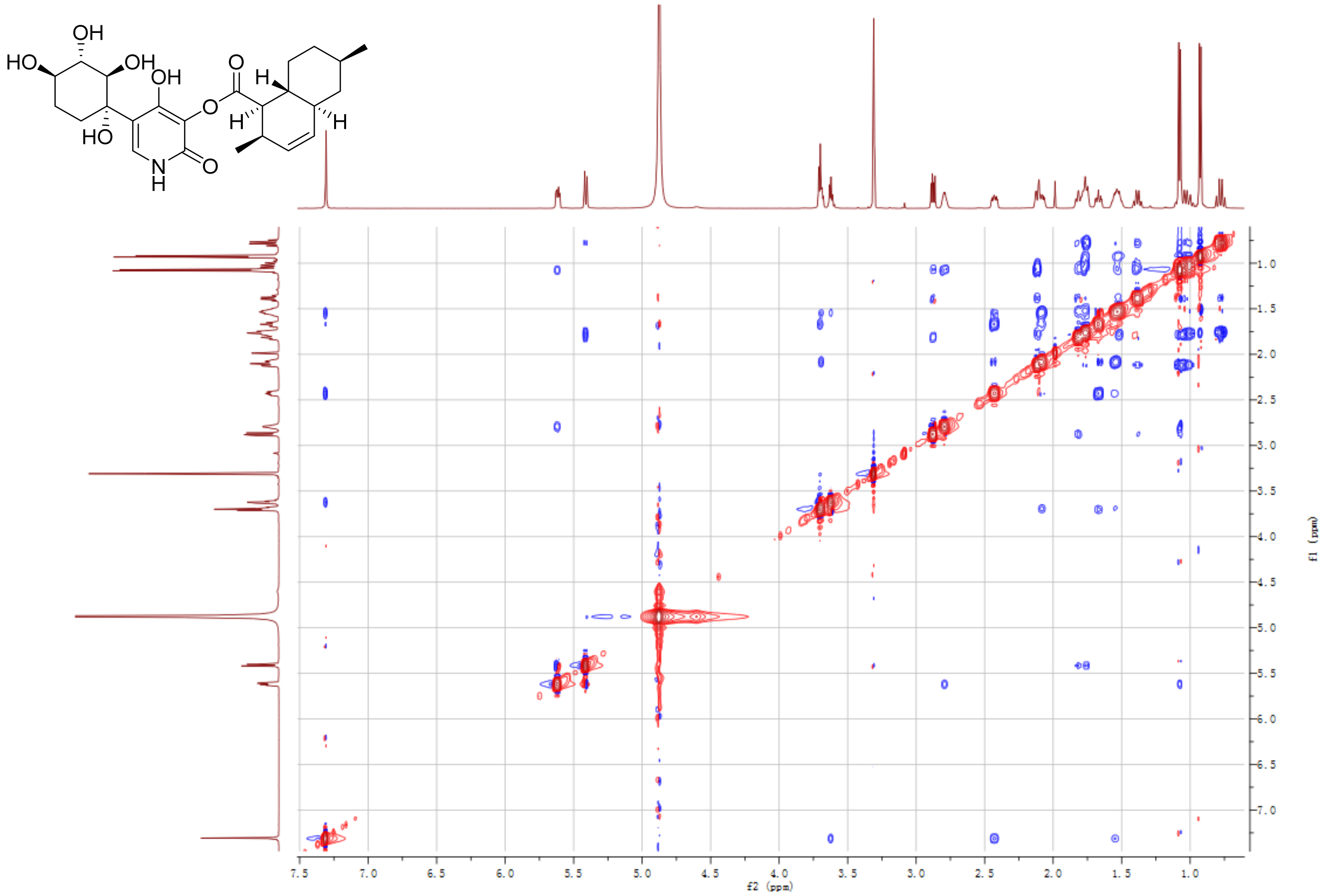

Figure S20. The ^{1}H-NMR spectrum of arthpyrone E **(2)** in DMSO-d_6

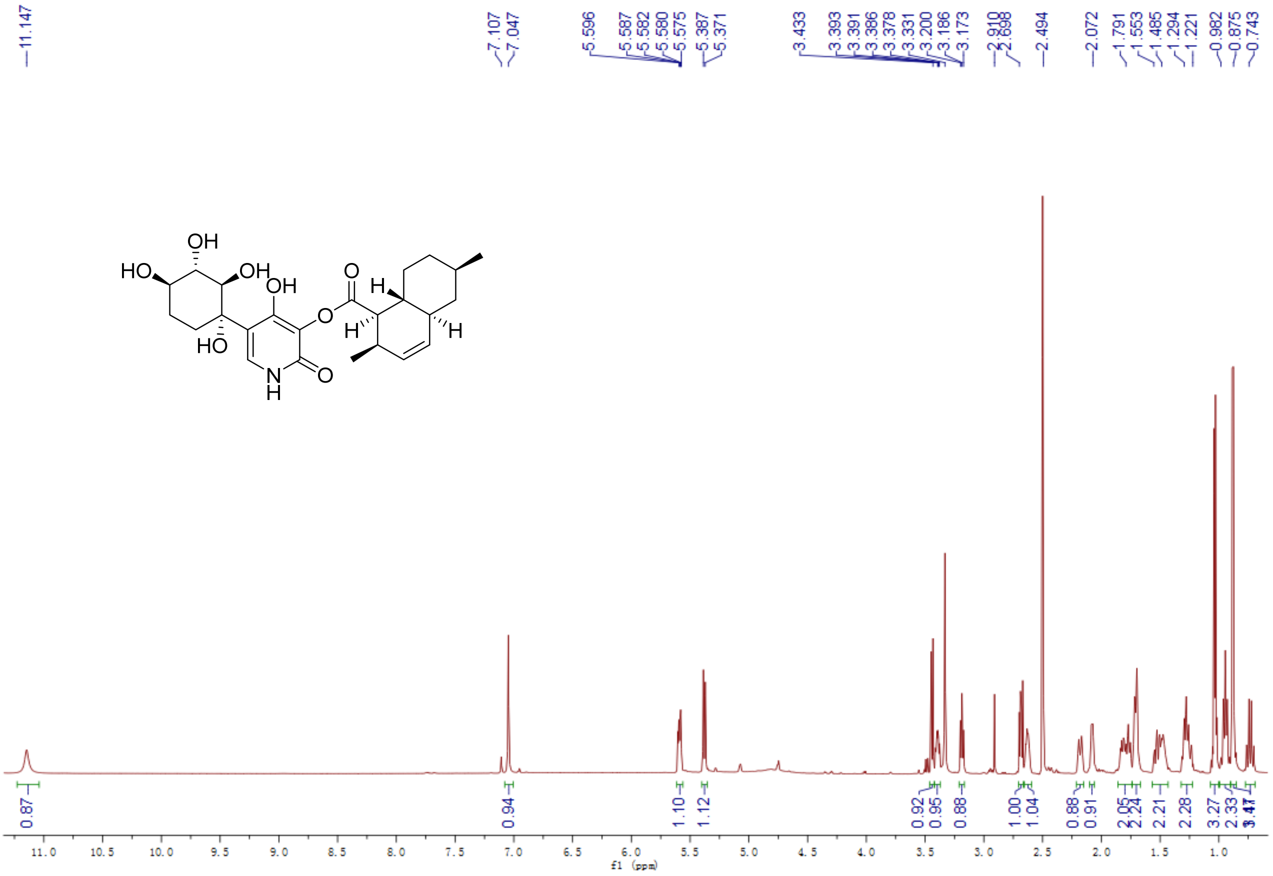

Figure S21. The ^{13}C-NMR spectrum of arthpyrone E **(2)** in DMSO-d_6

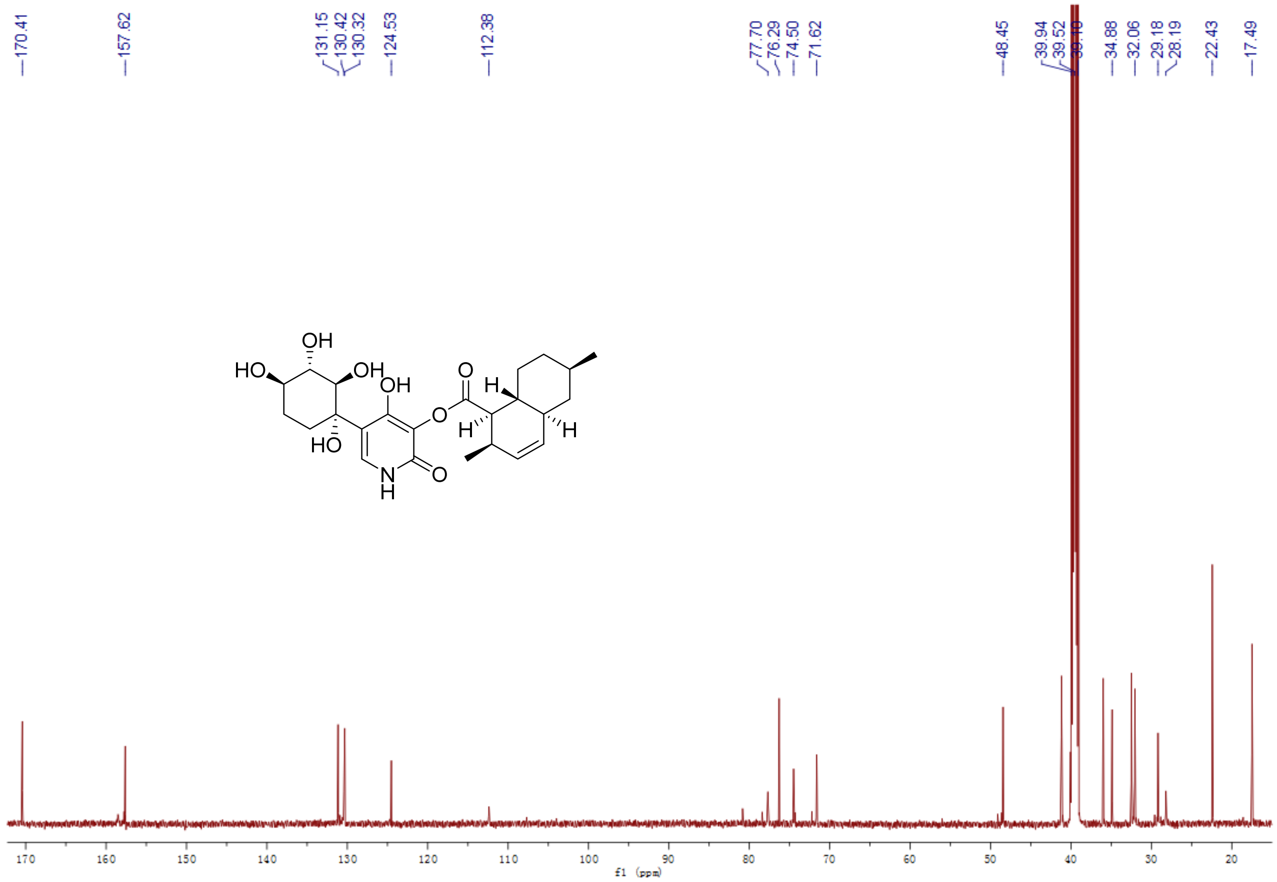

Figure S22. The HSQC spectrum of arthpyrone E **(2)** in DMSO-d_6

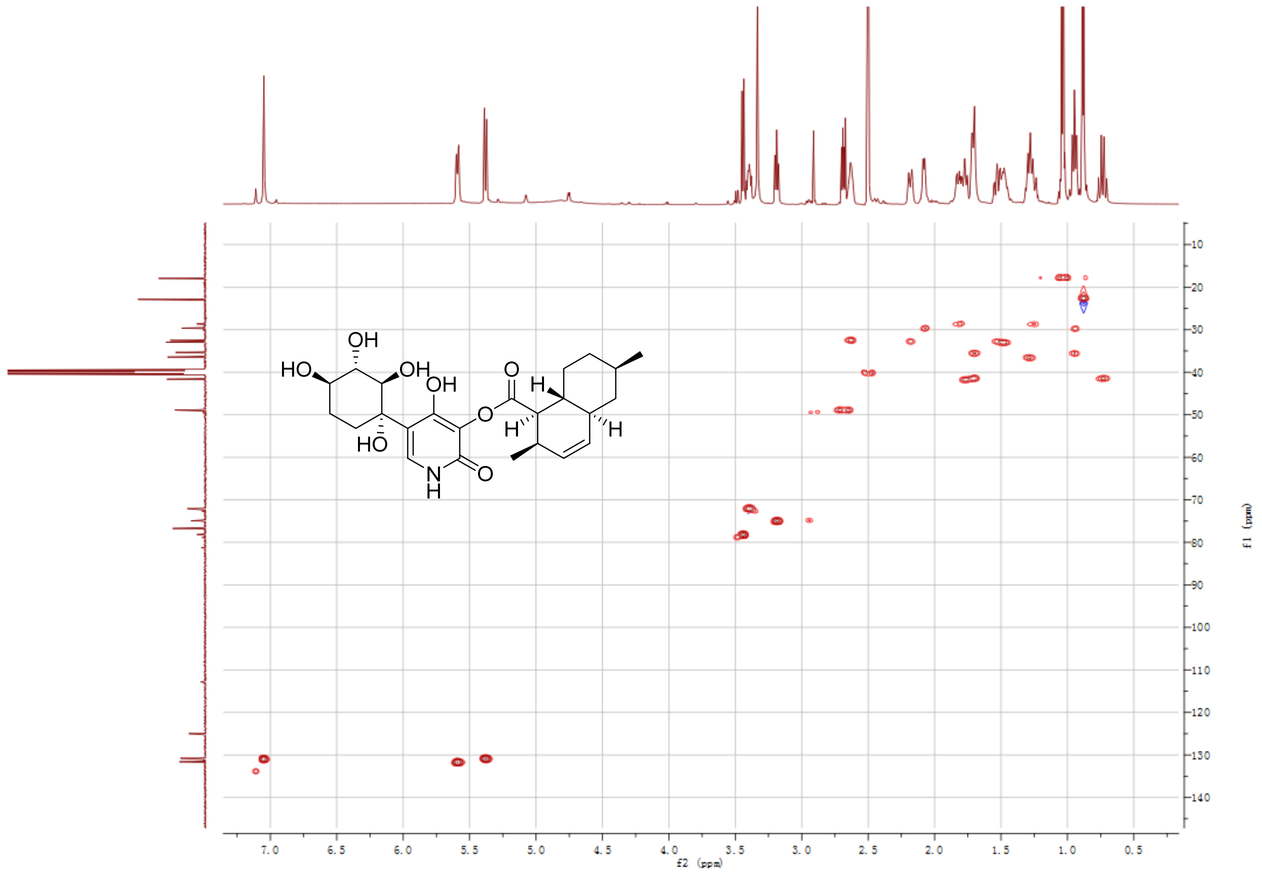

Figure S23. The ^{1}H-^{1}H COSY spectrum of arthpyrone E **(2)** in DMSO-d_6

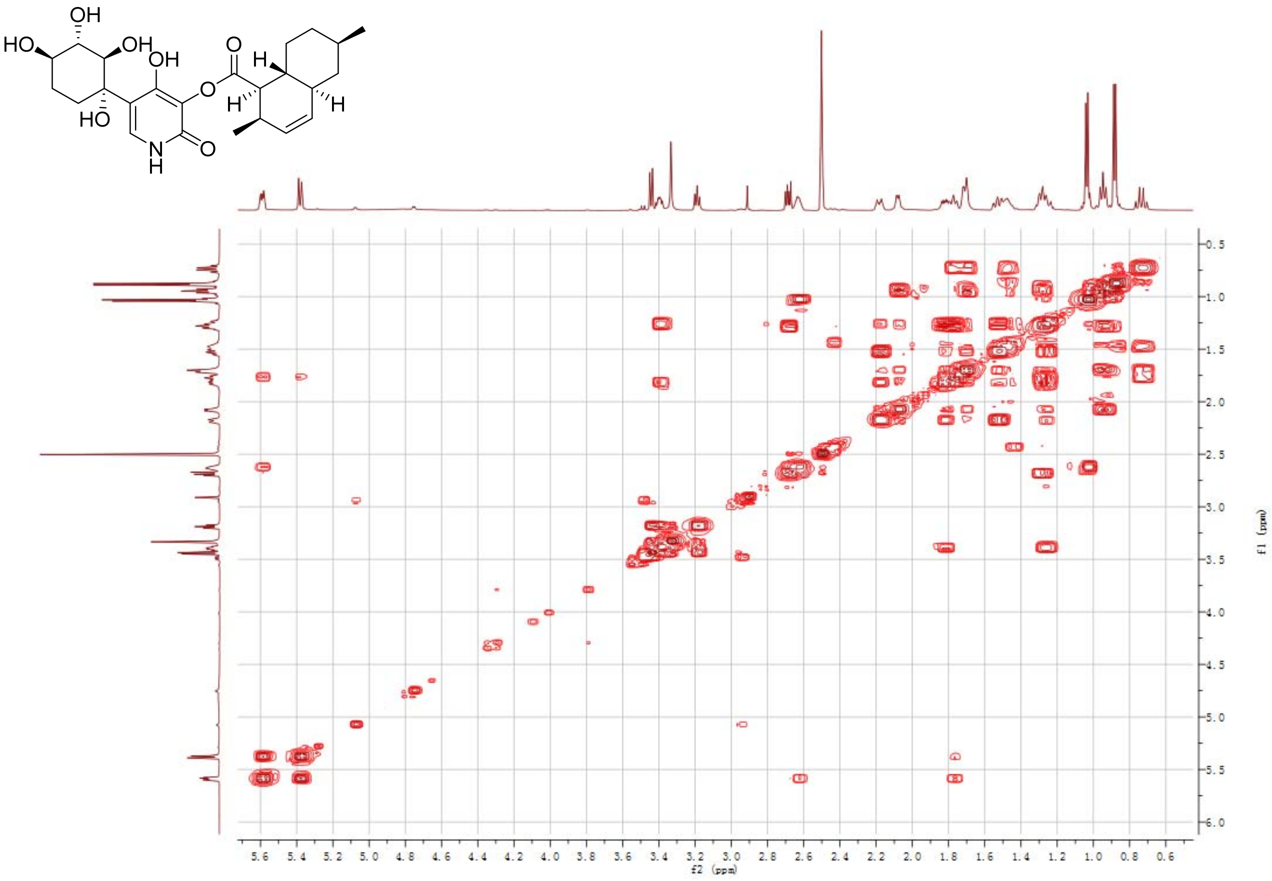

Figure S24. The NOESY spectrum of arthpyrone E **(2)** in DMSO-d_6

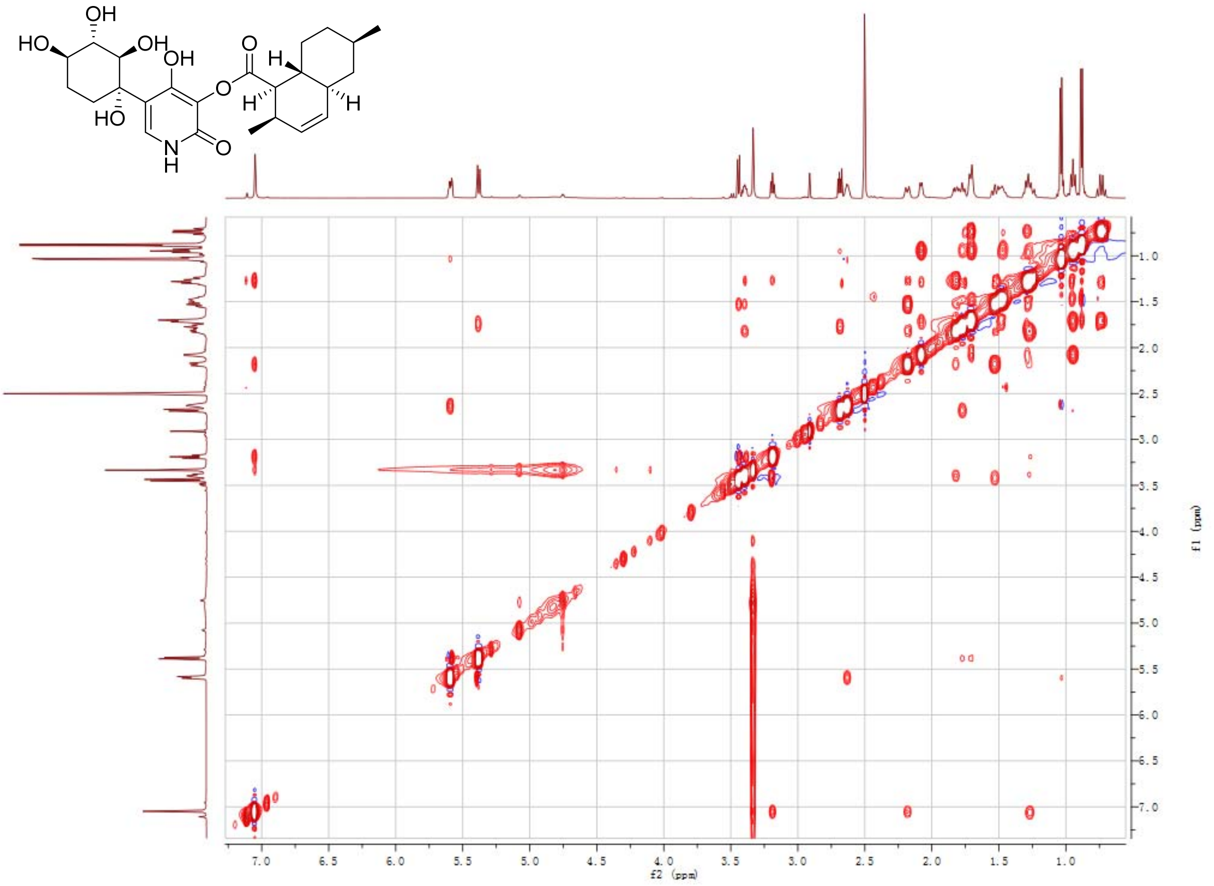

Figure S25. The (-)-HRESIMS spectrum of arthpyrone E **(2)**

**Sample Name**	BJ 58	**Position**	P1-A5	**Instrument Name**	Instrument 1
**User Name**		**Inj Vol**	2	**InjPosition**	
**Sample Type**	Sample	**IRM Calibration Status**	Success	**Data Filename**	BJ 58.d
**ACQ Method**	wss-isocratic elution-Negative.m	**Comment**		**Acquired Time**	11/23/2017 9:18:02 AM

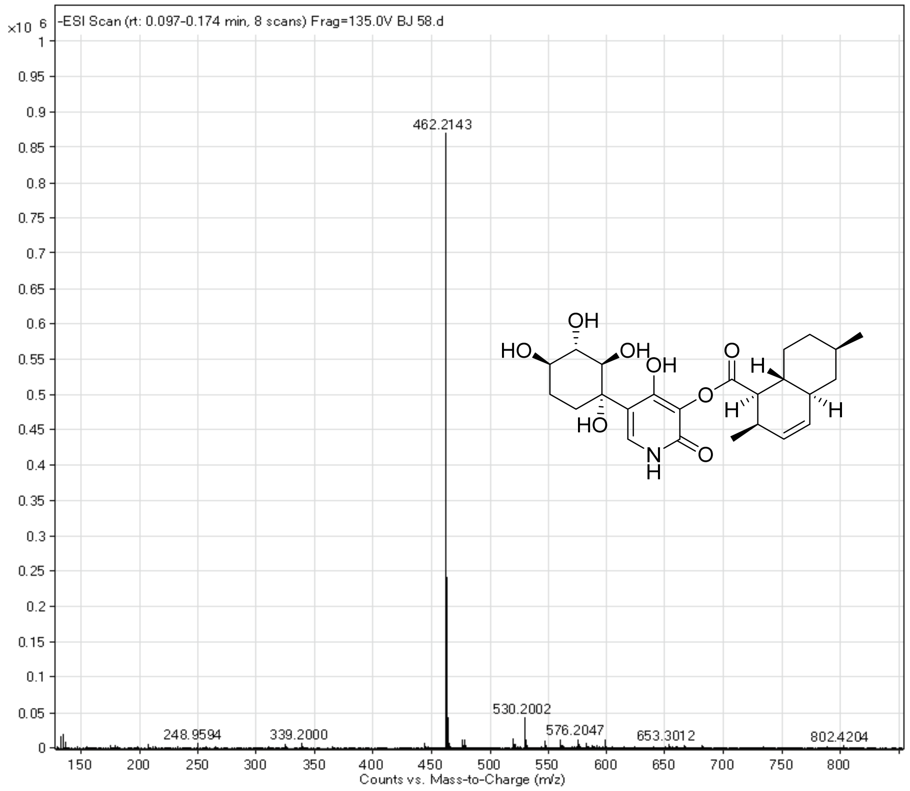

Figure S26. The ^{1}H-NMR spectrum of arthpyrone F **(3)** in CD_3OD

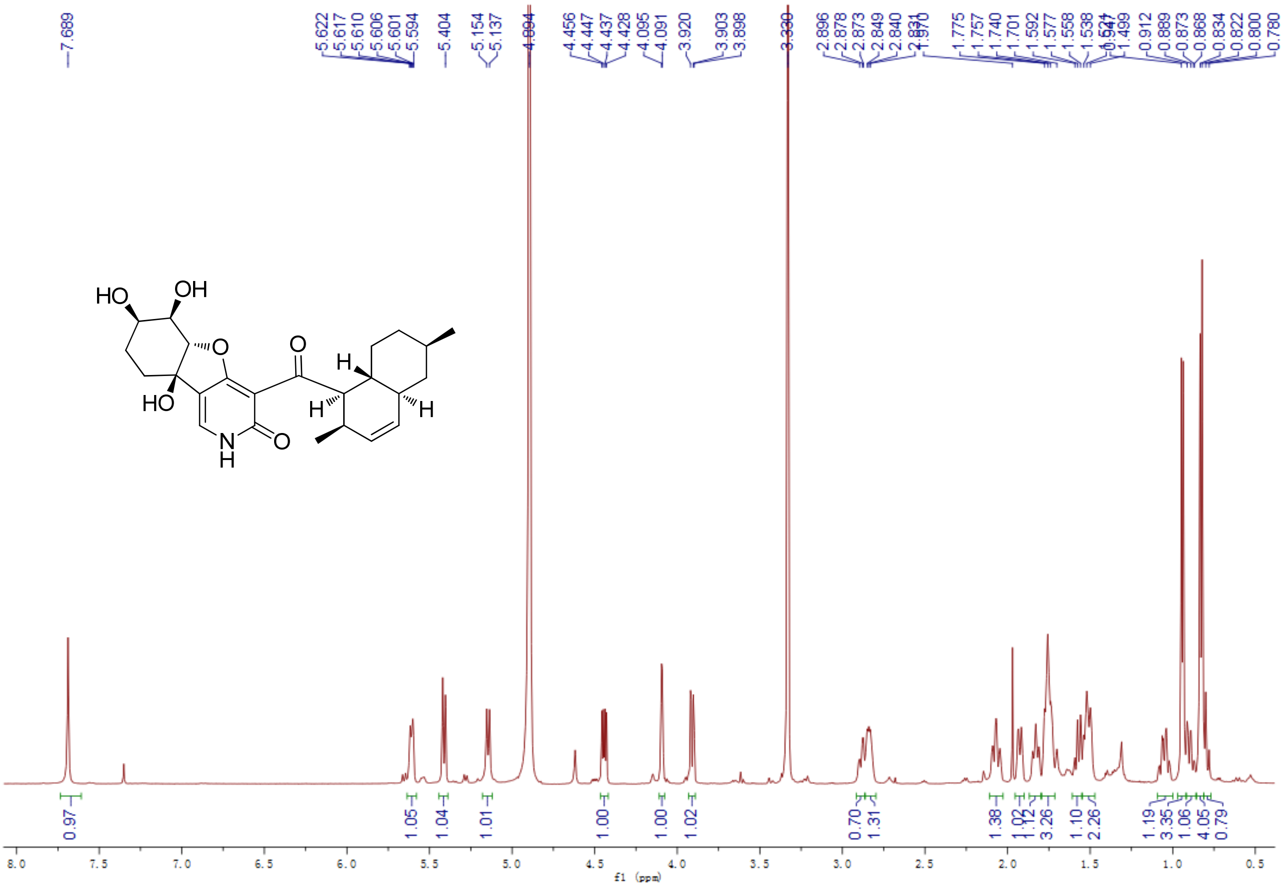

Figure S27. The ^{13}C-NMR spectrum of arthpyrone F **(3)** in CD_3OD

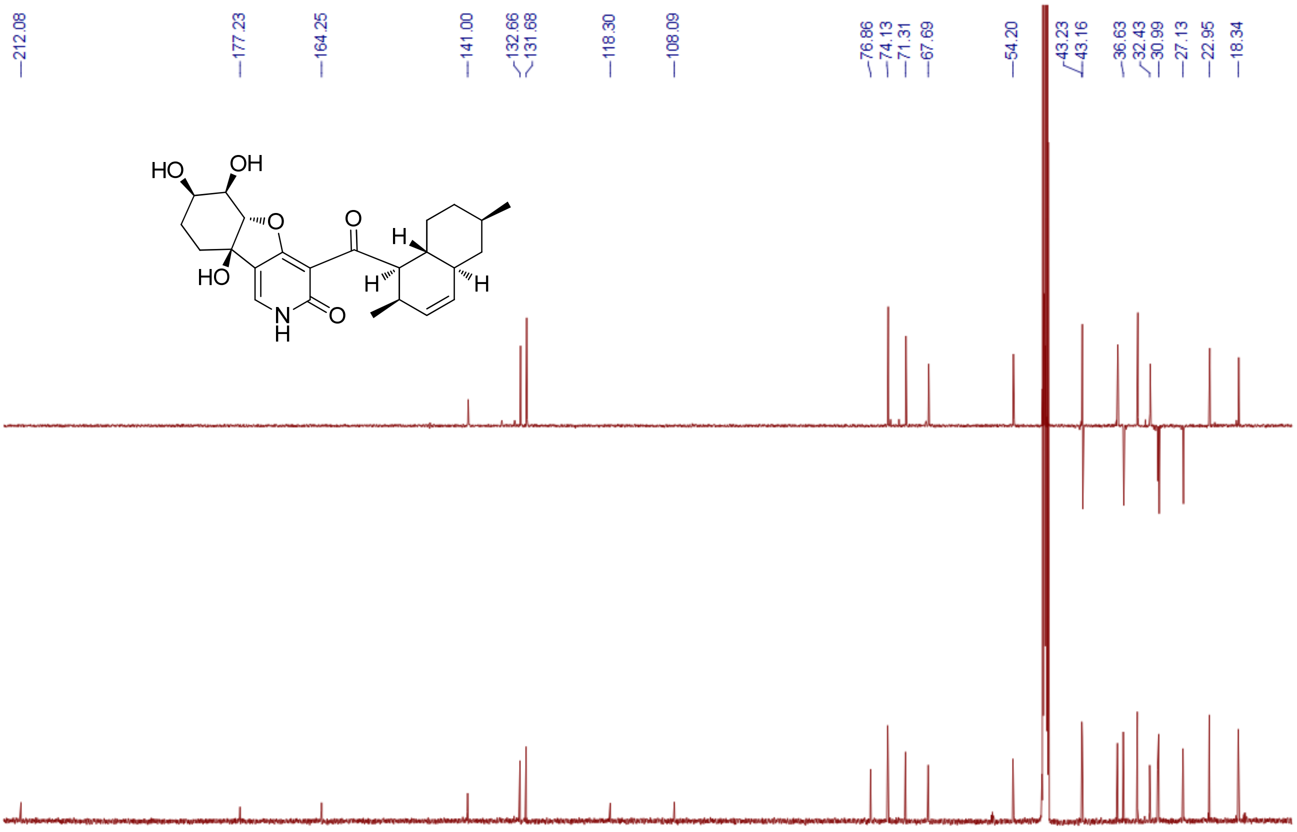

Figure S28. The HSQC spectrum of arthpyrone F **(3)** in CD_3OD

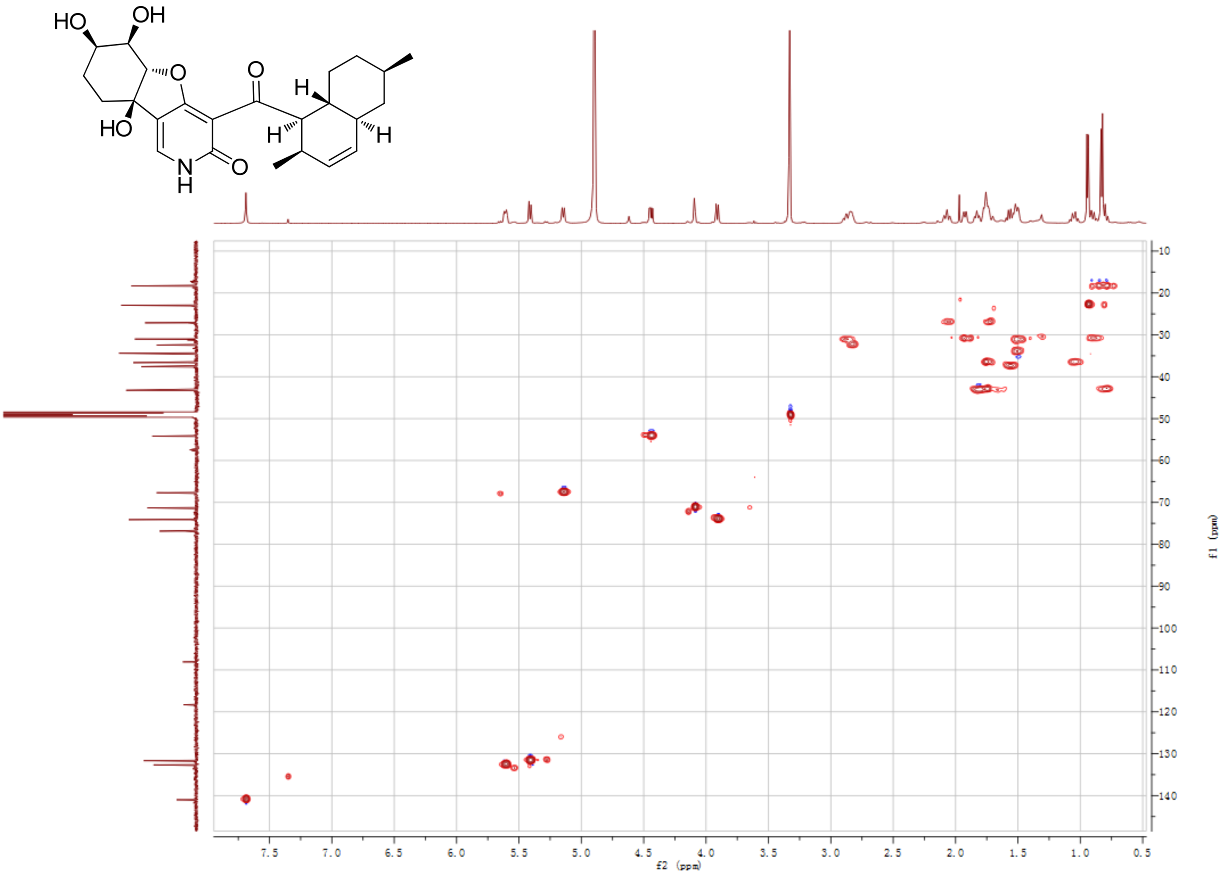

Figure S29. The ^{1}H-^{1}H COSY spectrum of arthpyrone F **(3)** in CD_3OD

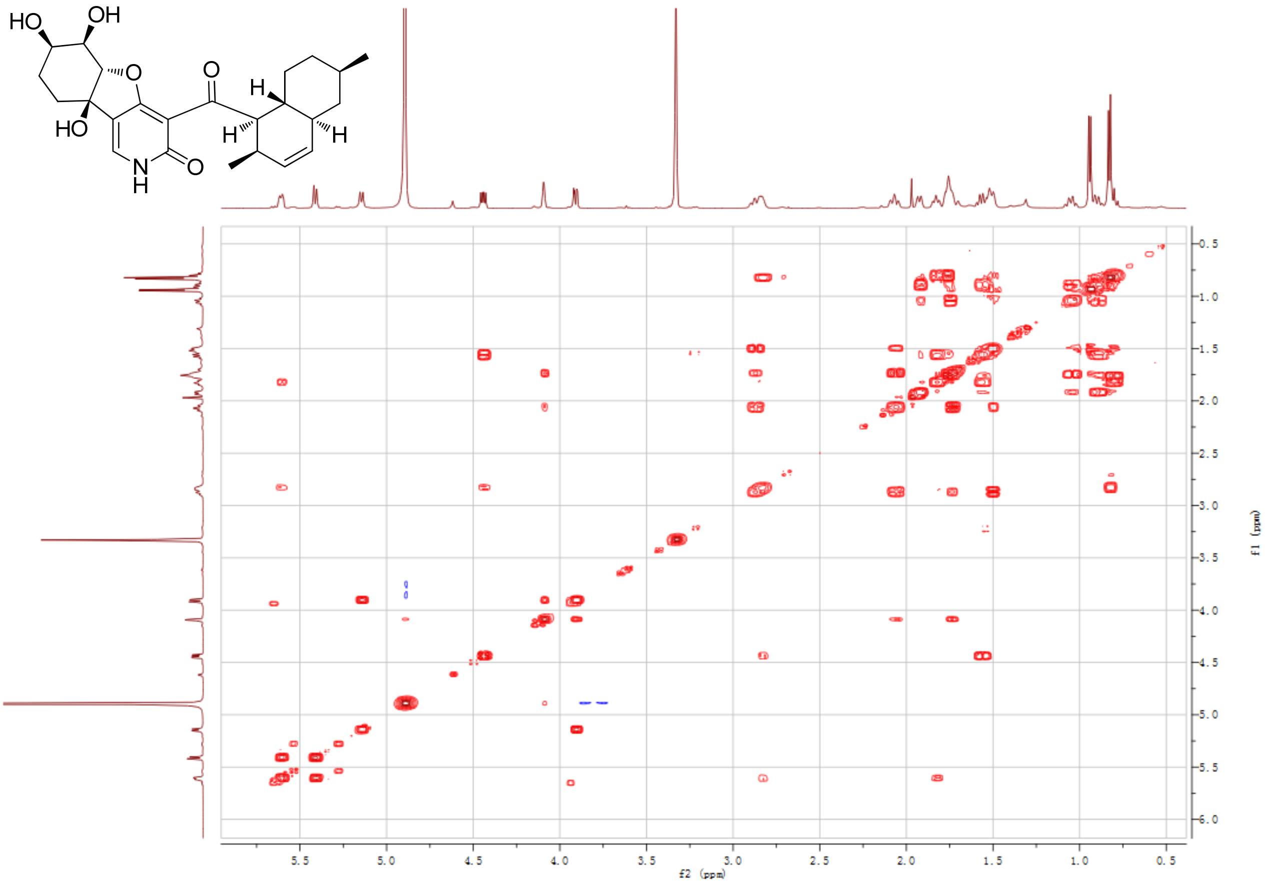

Figure S30. The HMBC spectrum of arthpyrone F **(3)** in CD_3OD

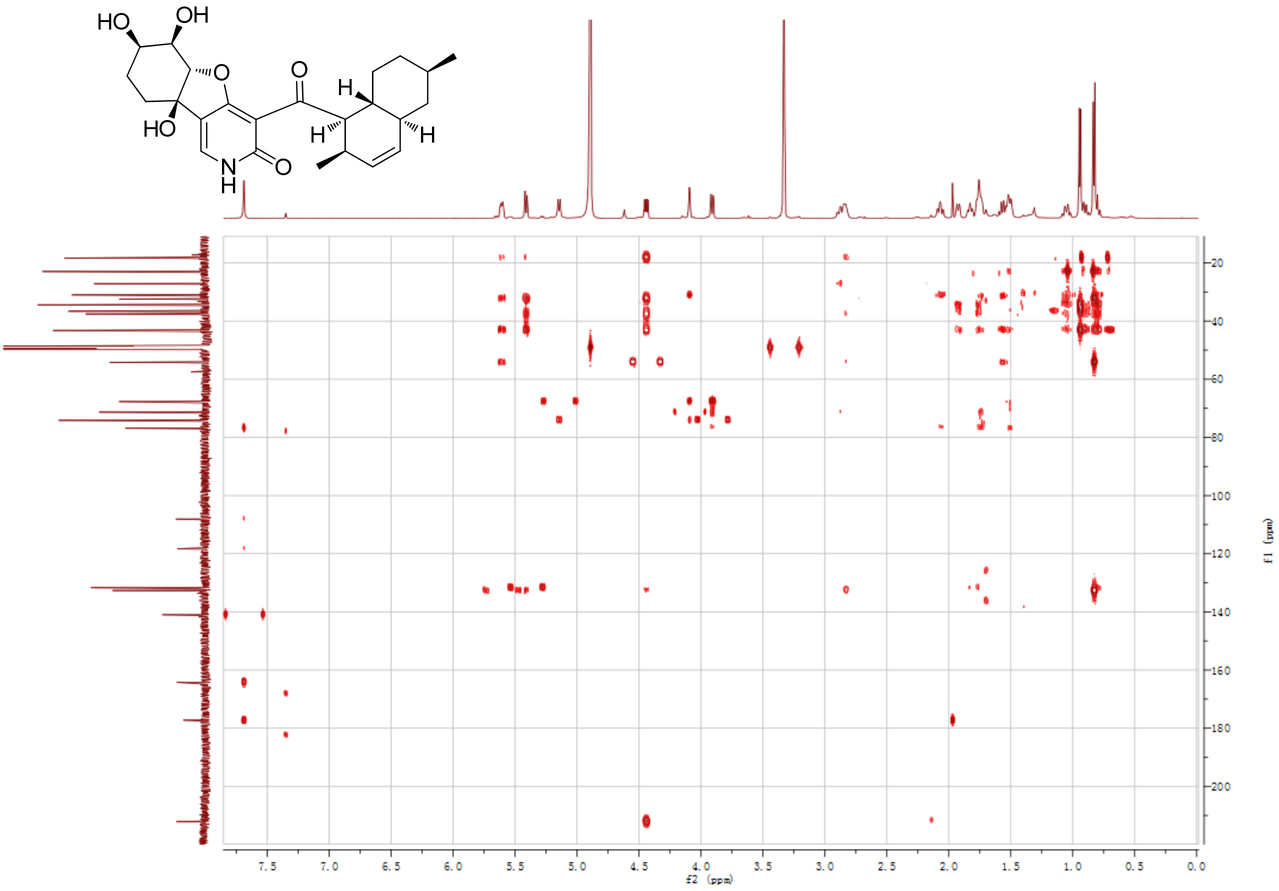

Figure S31. The NOESY spectrum of arthpyrone F **(3)** in CD_3OD

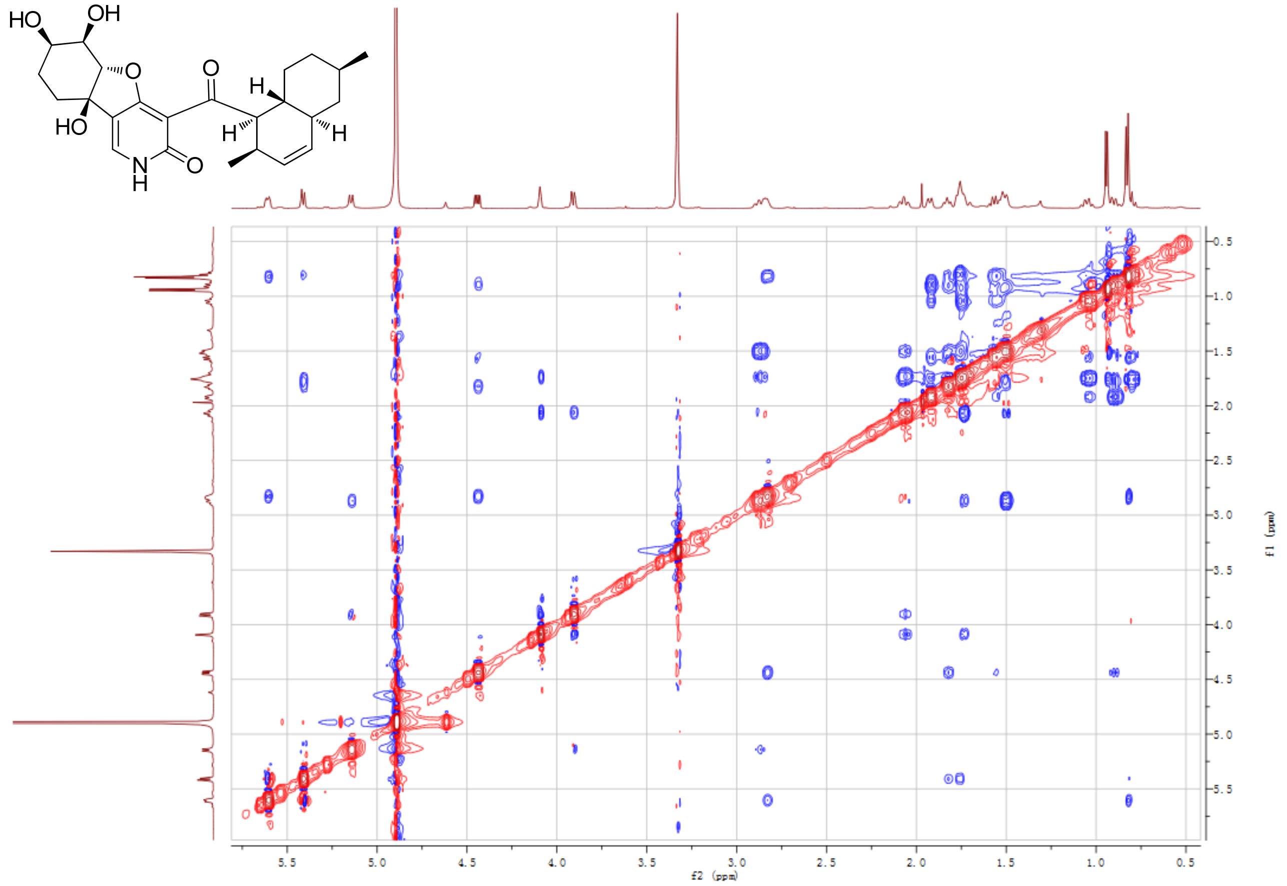

Figure S32. The ^{1}H-NMR spectrum of arthpyrone F **(3)** in DMSO-d_6

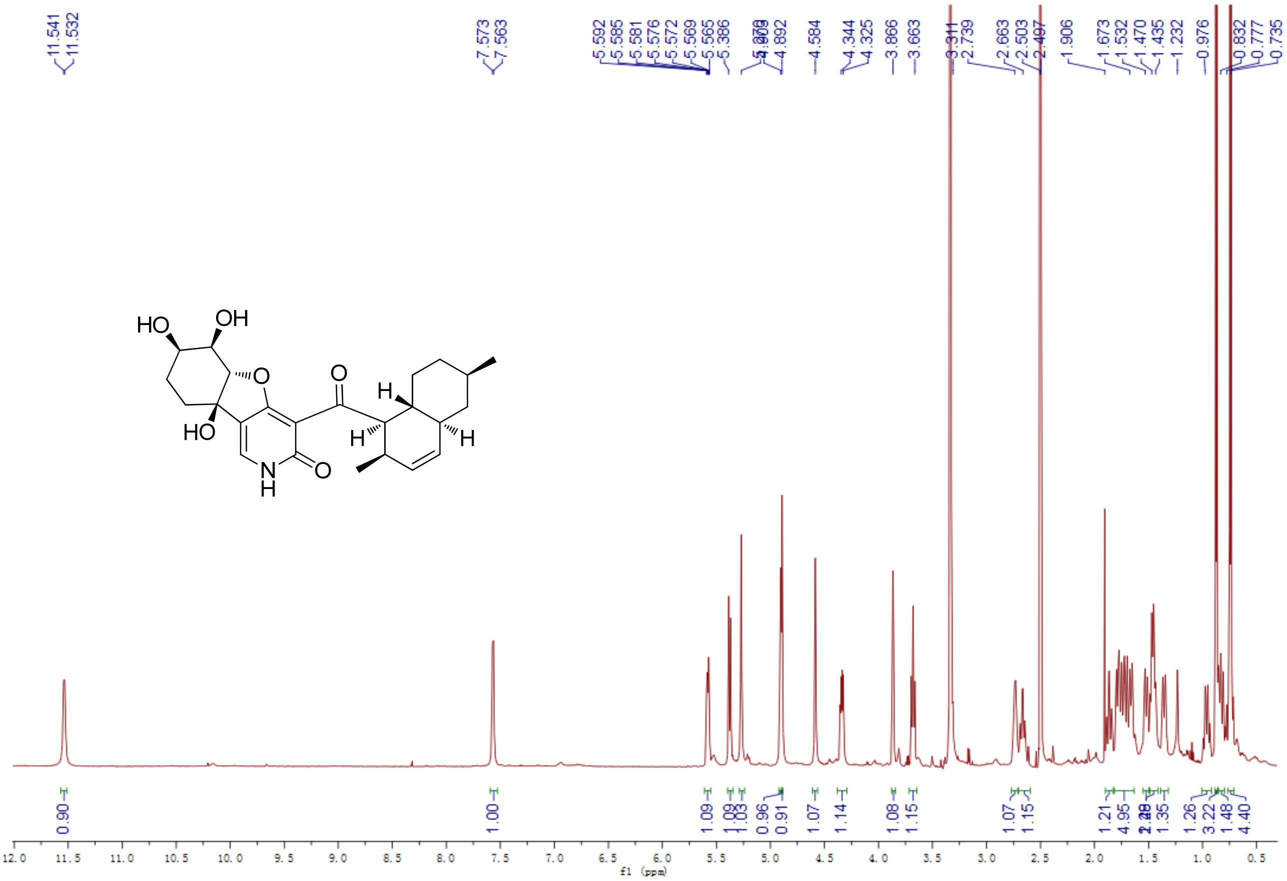

Figure S33. The ^{13}C-NMR spectrum of arthpyrone F **(3)** in DMSO-d_6

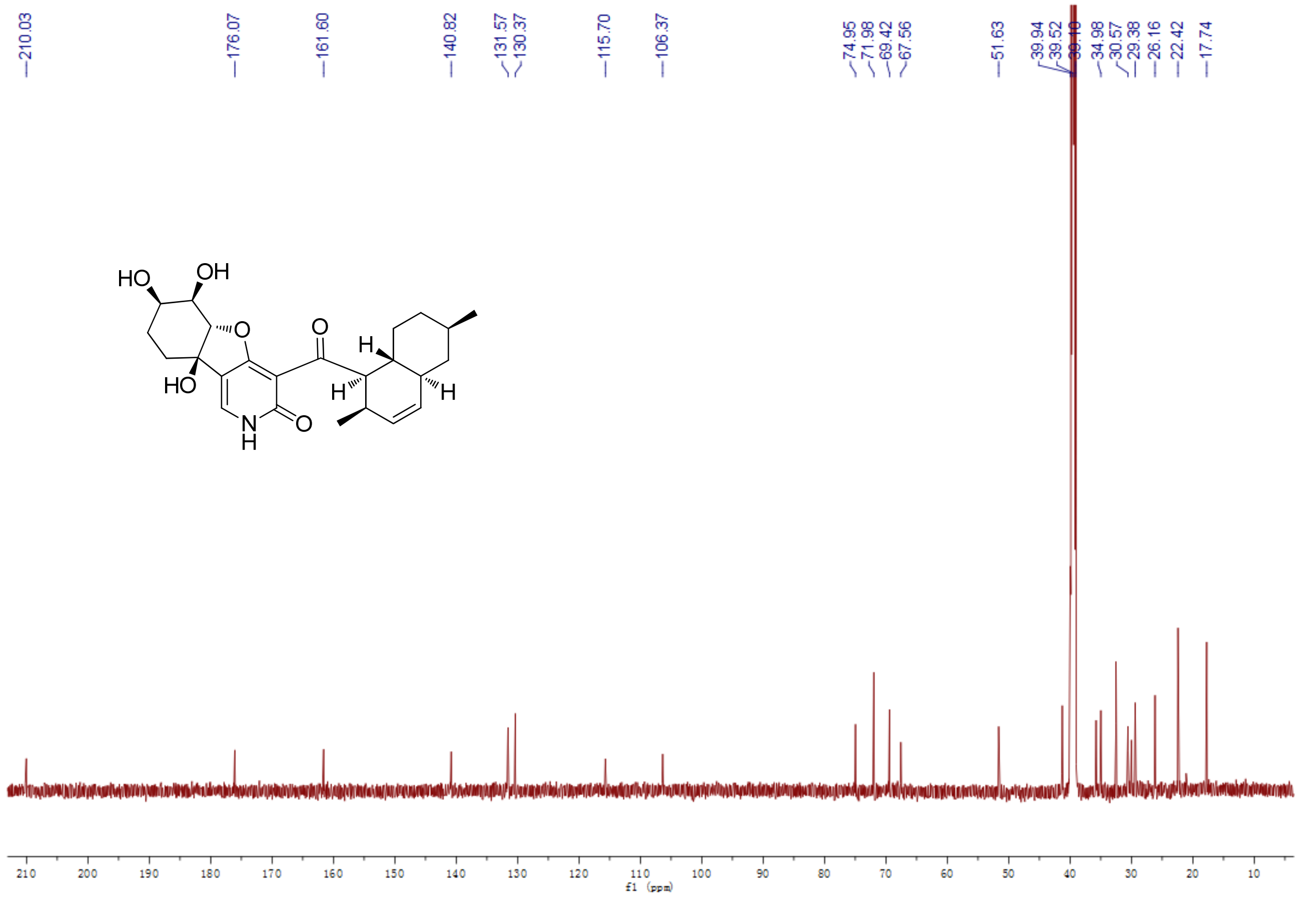

Figure S34. The HSQC spectrum of arthpyrone F **(3)** in DMSO-d_6

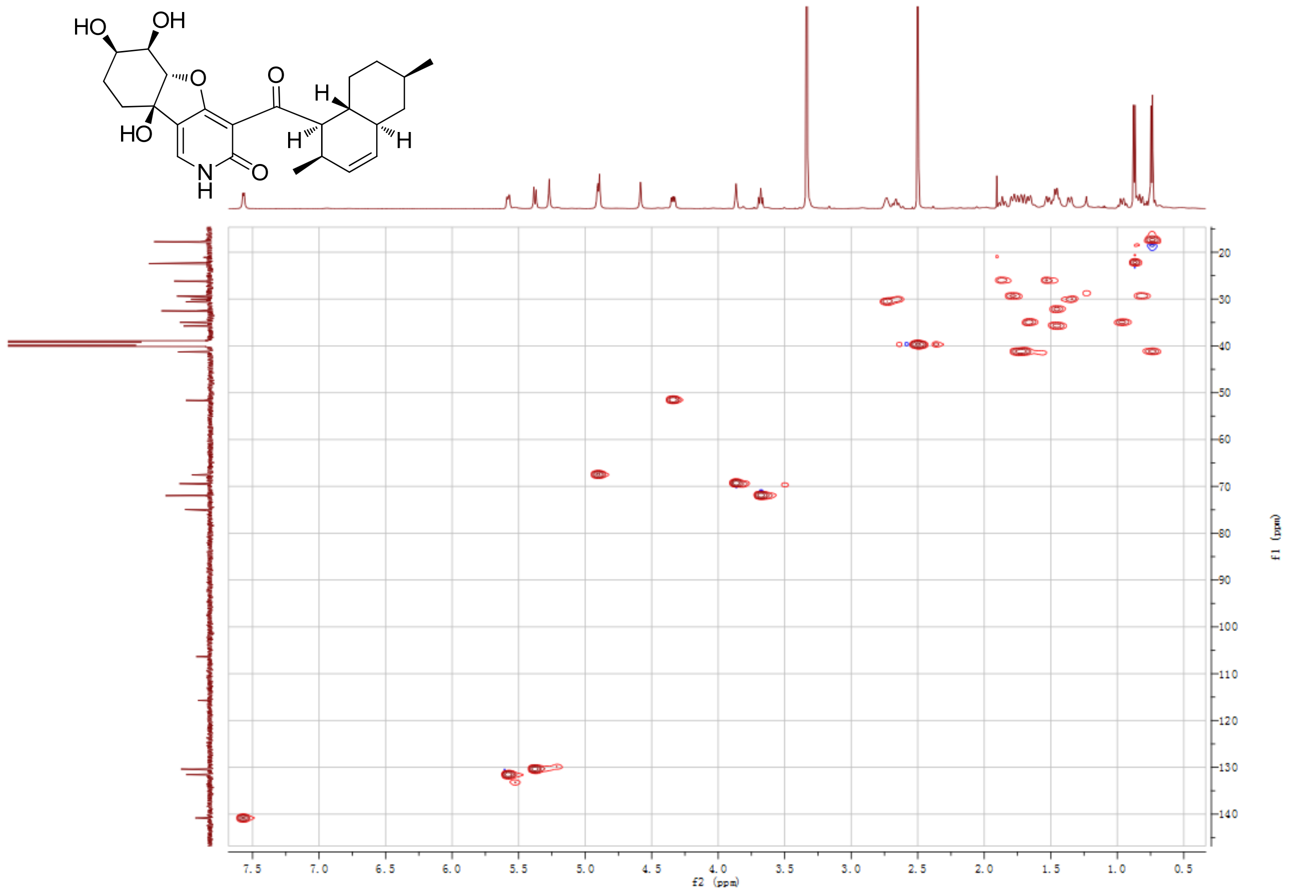

Figure S35. The ^{1}H-^{1}H COSY spectrum of arthpyrone F **(3)** in DMSO-d_6

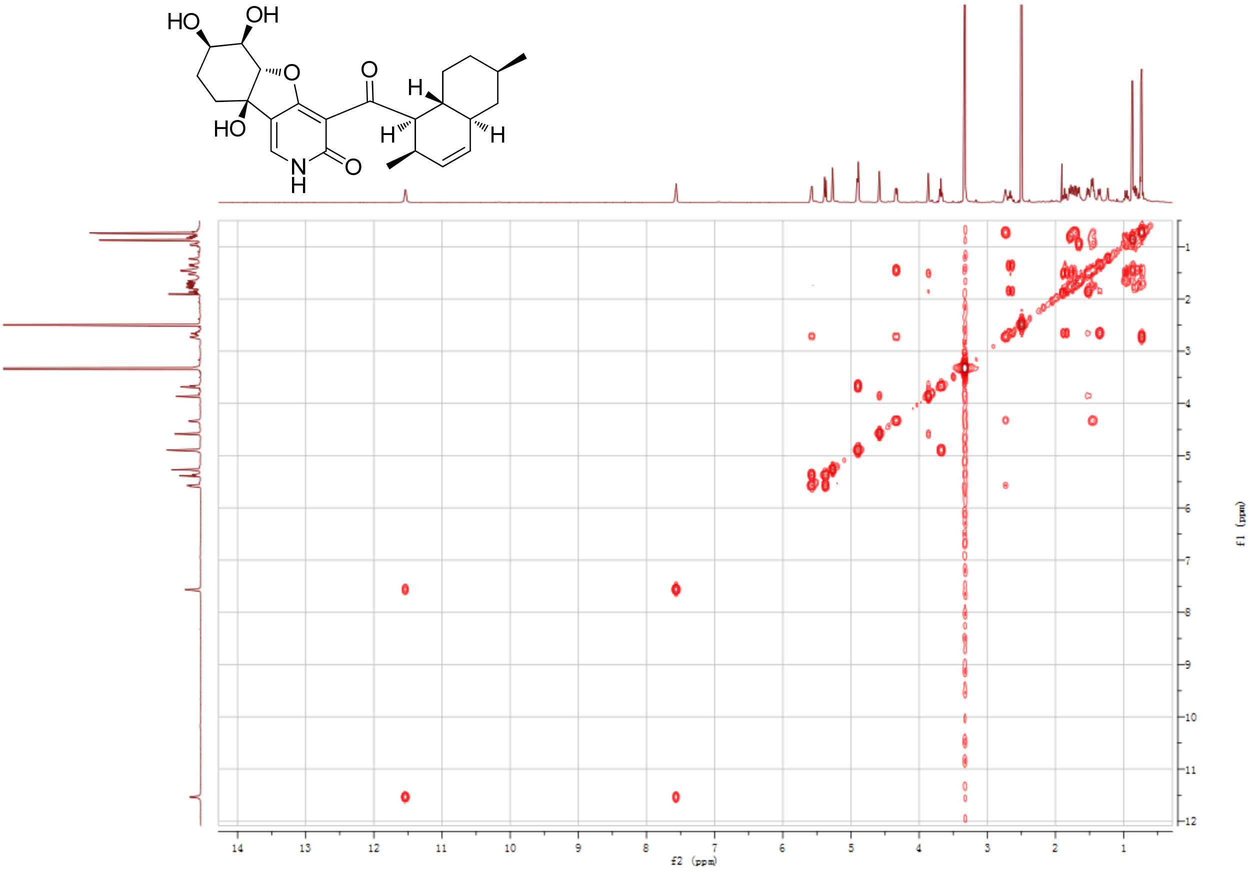

Figure S36. The HMBC spectrum of arthpyrone F **(3)** in DMSO-d_6

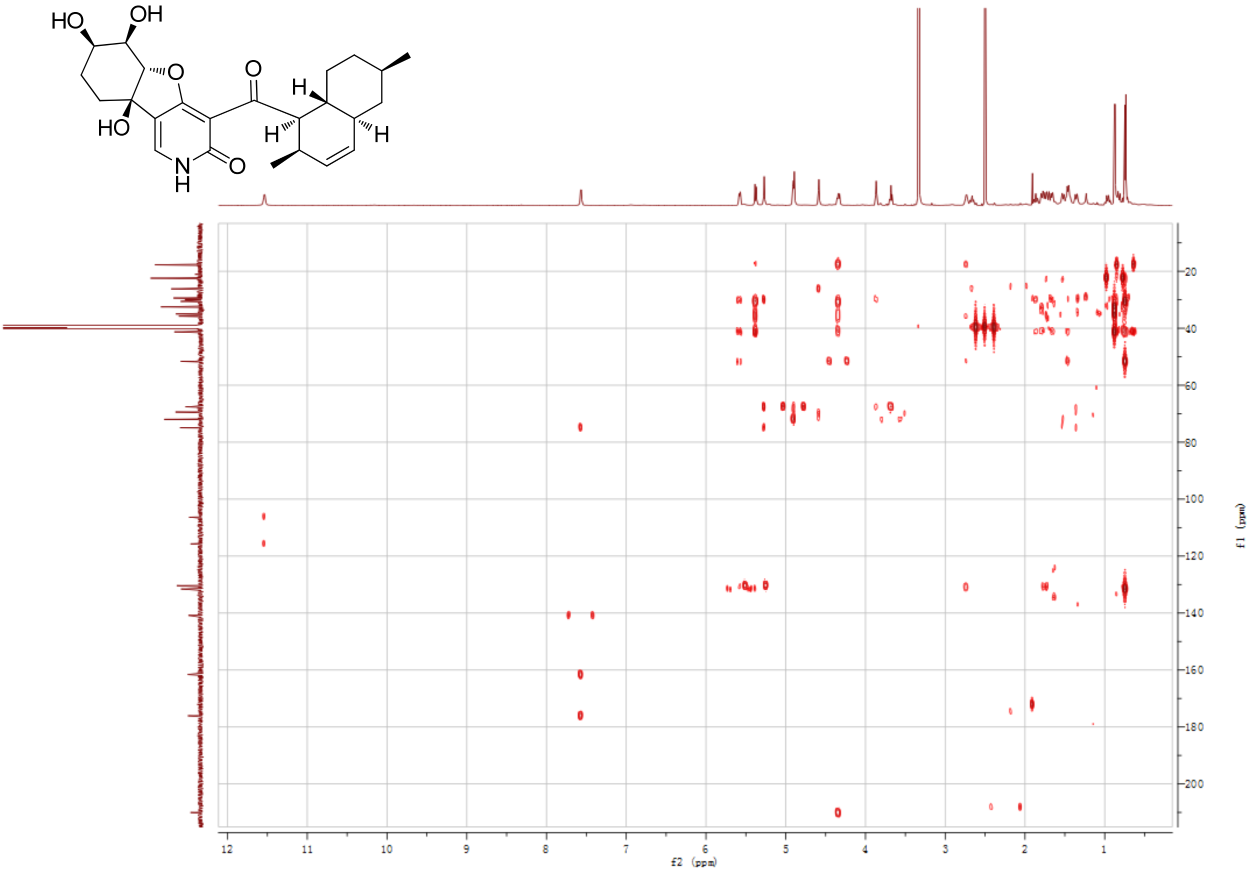

Figure S37. The NOESY spectrum of arthpyrone F **(3)** in DMSO-d_6

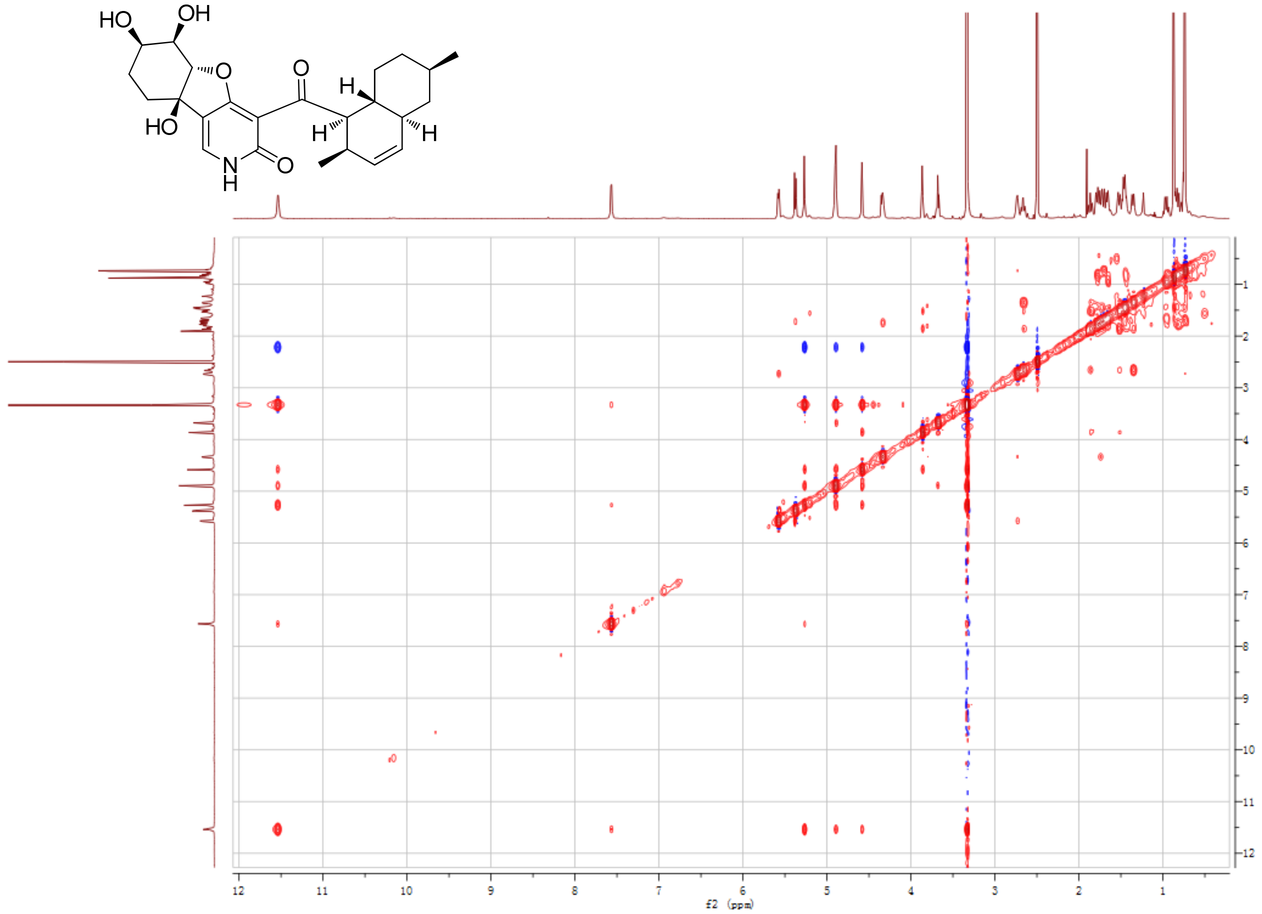

Figure S38. The (-)-HRESIMS spectrum of arthpyrone F **(3)**

**Sample Name**	BJ 67-1	**Position**	P1-A9	**Instrument Name**	Instrument 1
**User Name**		**Inj Vol**	2	**InjPosition**	
**Sample Type**	Sample	**IRM Calibration Status**	Success	**Data Filename**	BJ 67-1.d
**ACQ Method**	wss-isocratic elution-Negative.m	**Comment**		**Acquired Time**	11/23/2017 9:28:50 AM

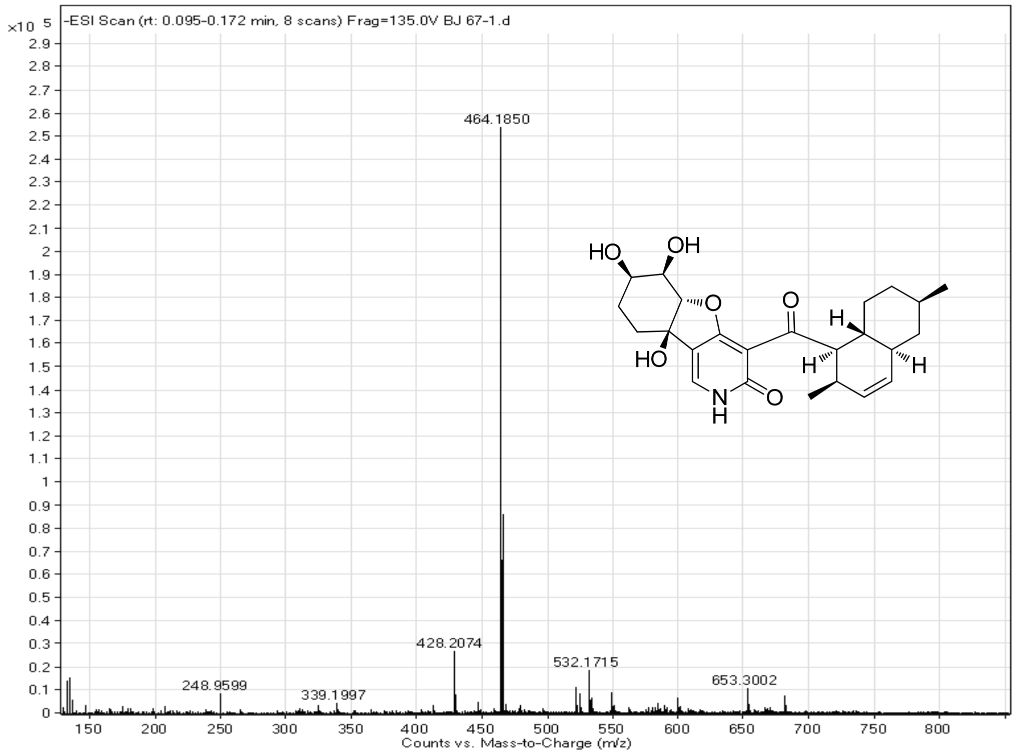

Figure S39. The ^{1}H-NMR spectrum of arthpyrone G **(4)** in CD_3OD

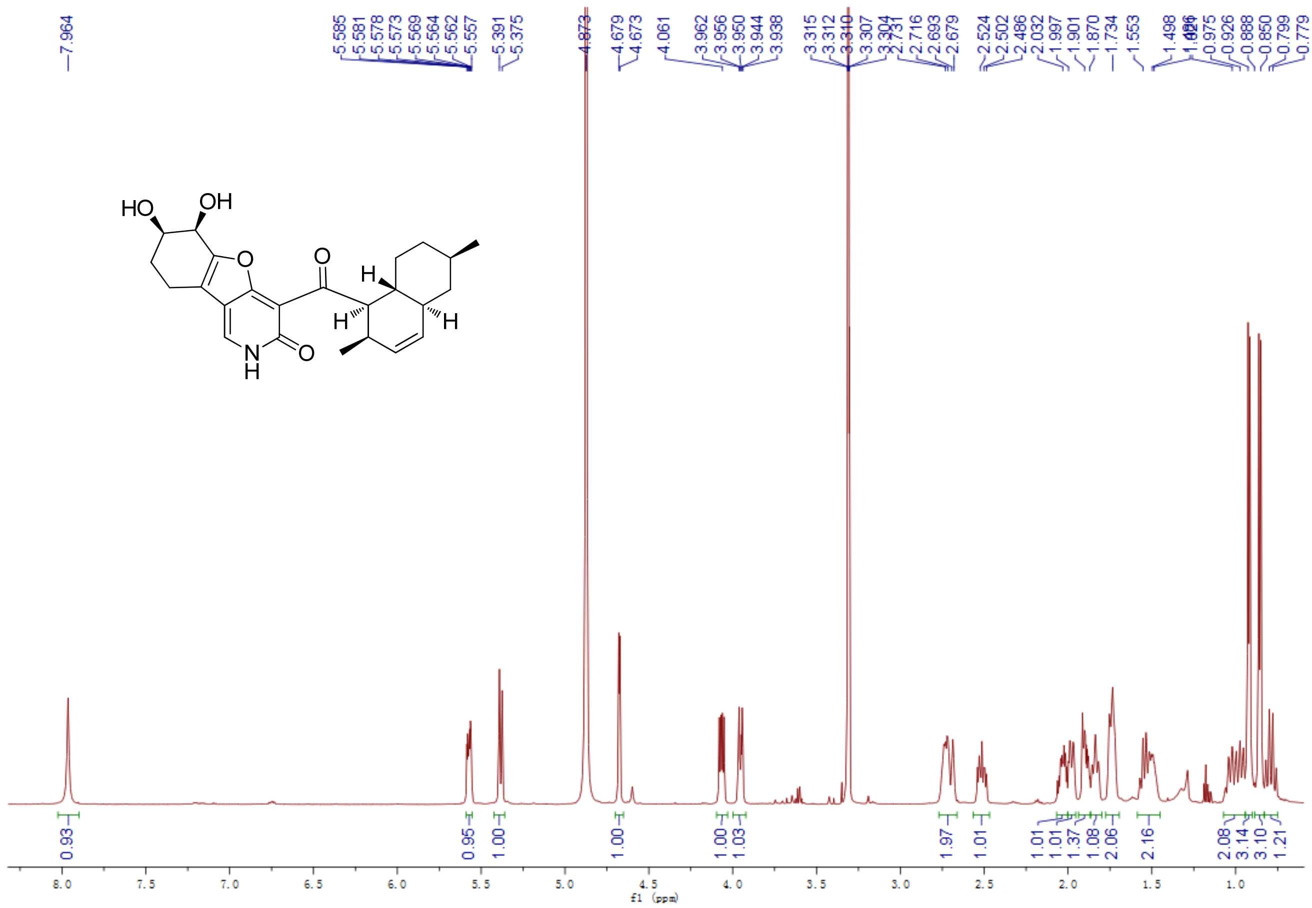

Figure S40. The ^{13}C-NMR spectrum of arthpyrone G **(4)** in CD_3OD

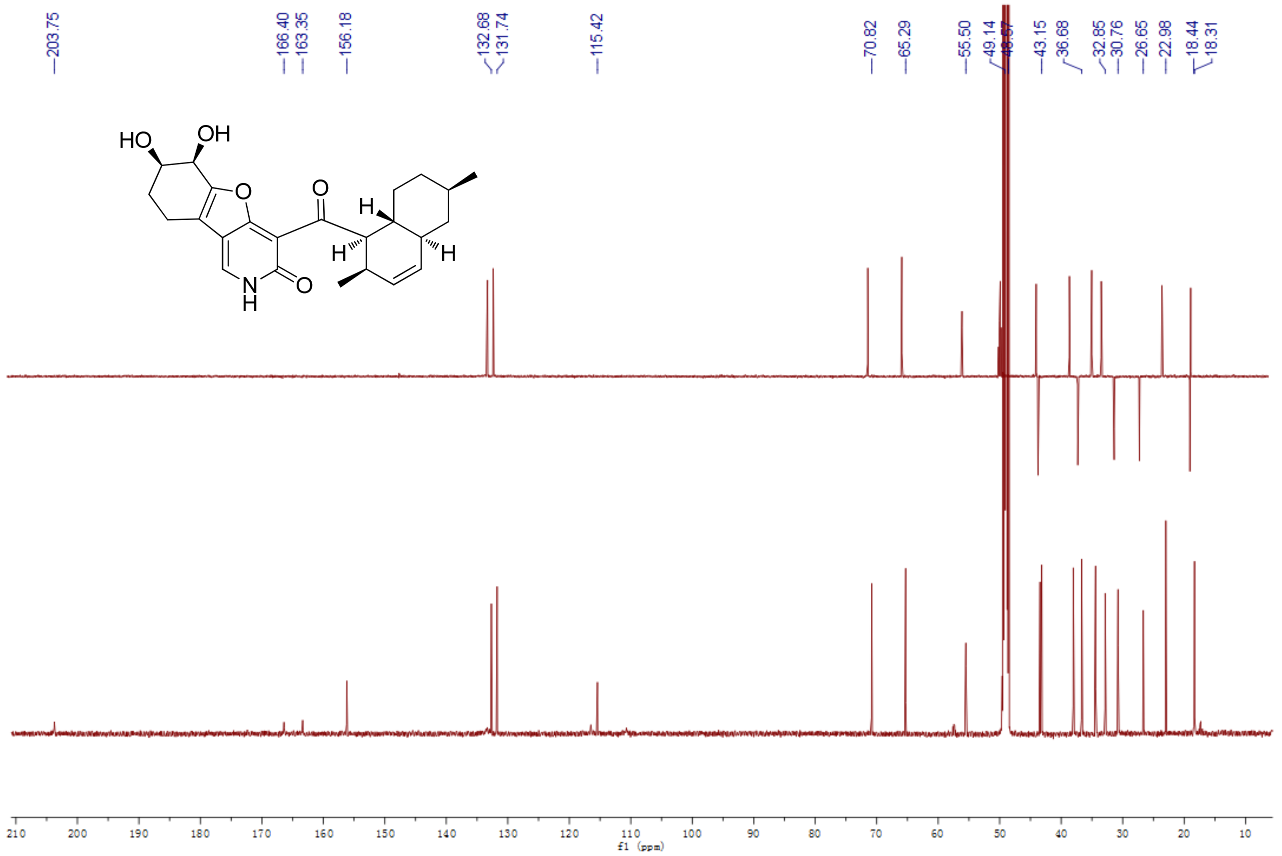

Figure S41. The HSQC spectrum of arthpyrone G **(4)** in CD_3OD

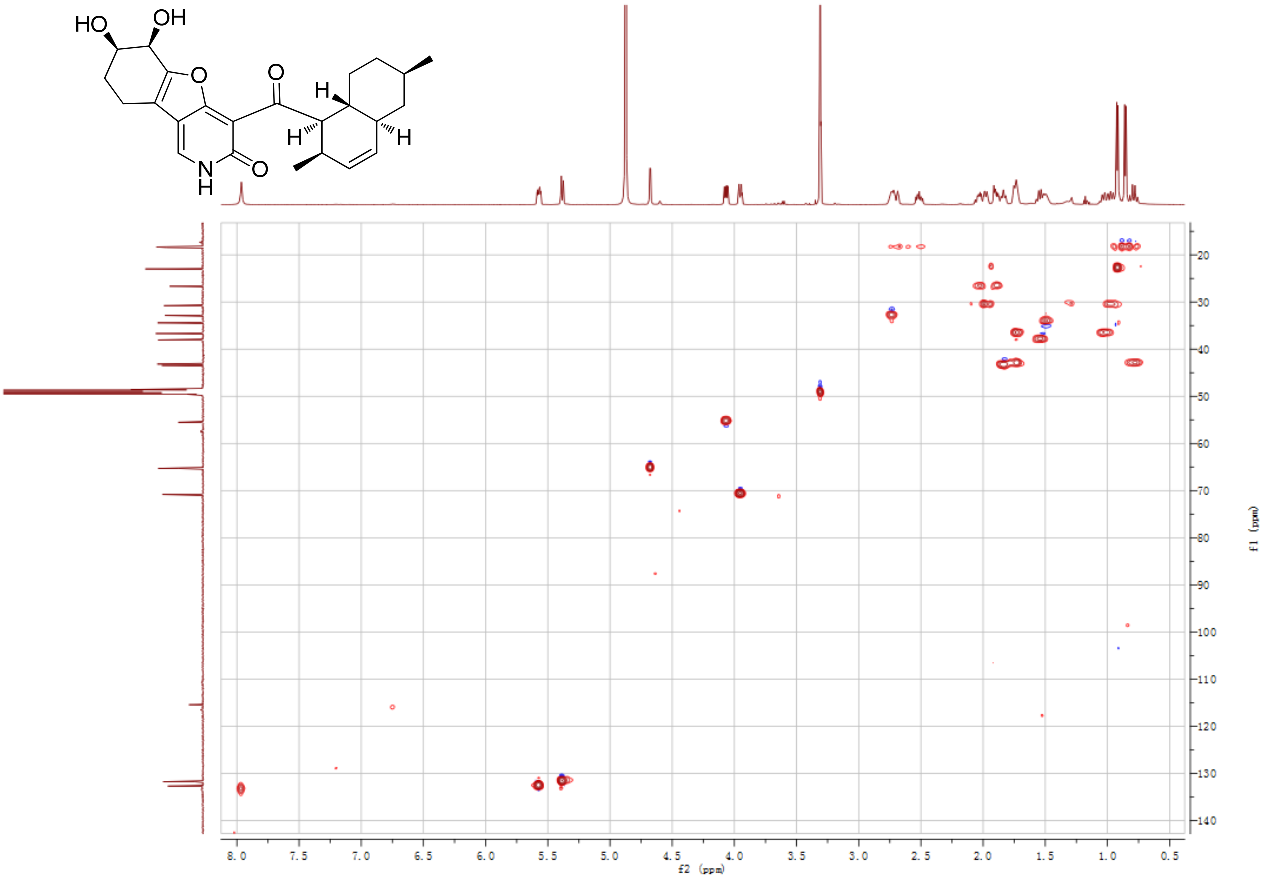

Figure S42. The ^{1}H-^{1}H COSY spectrum of arthpyrone G **(4)** inCD_3OD

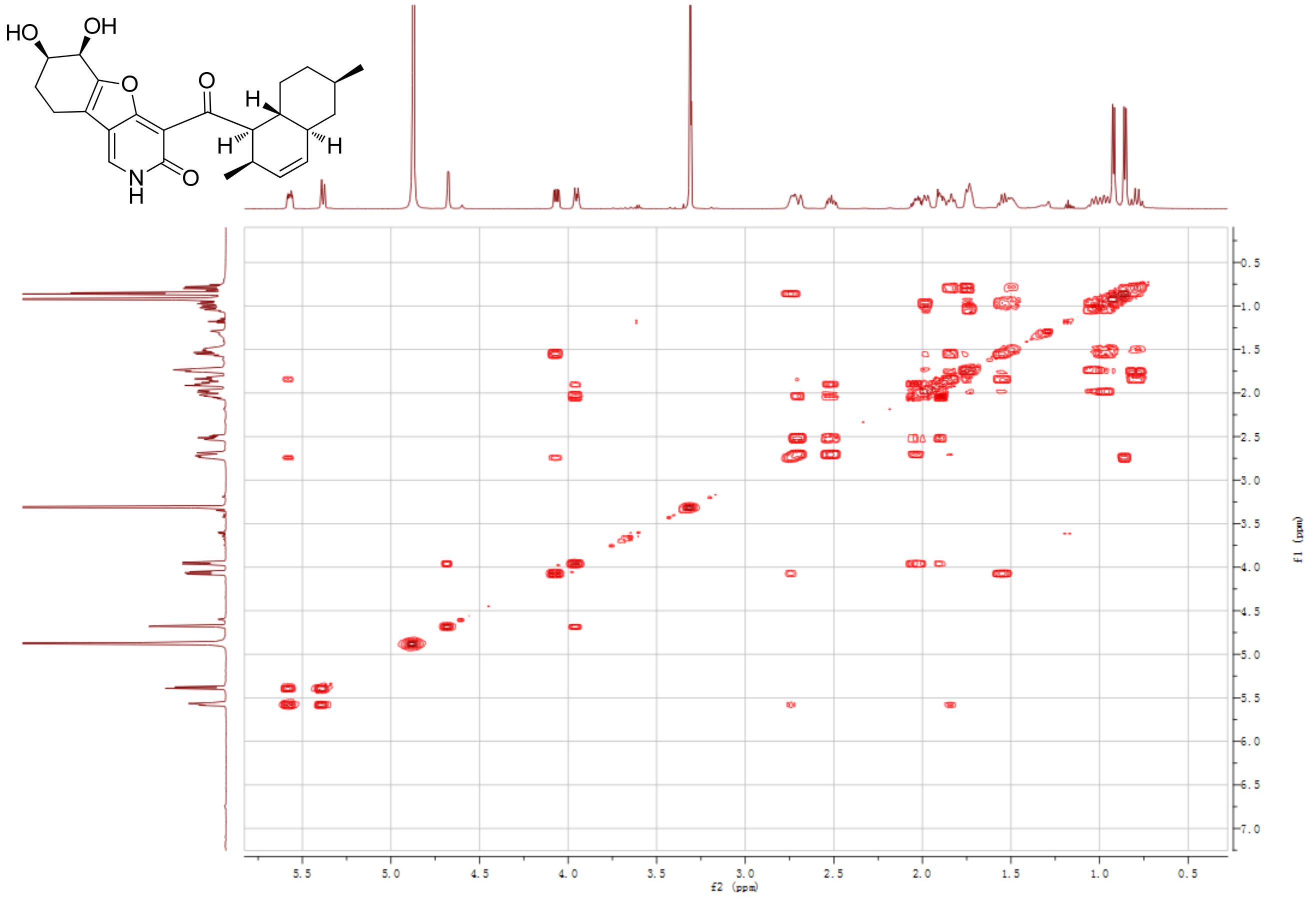

Figure S43. The HMBC spectrum of arthpyrone G **(4)** in CD_3OD

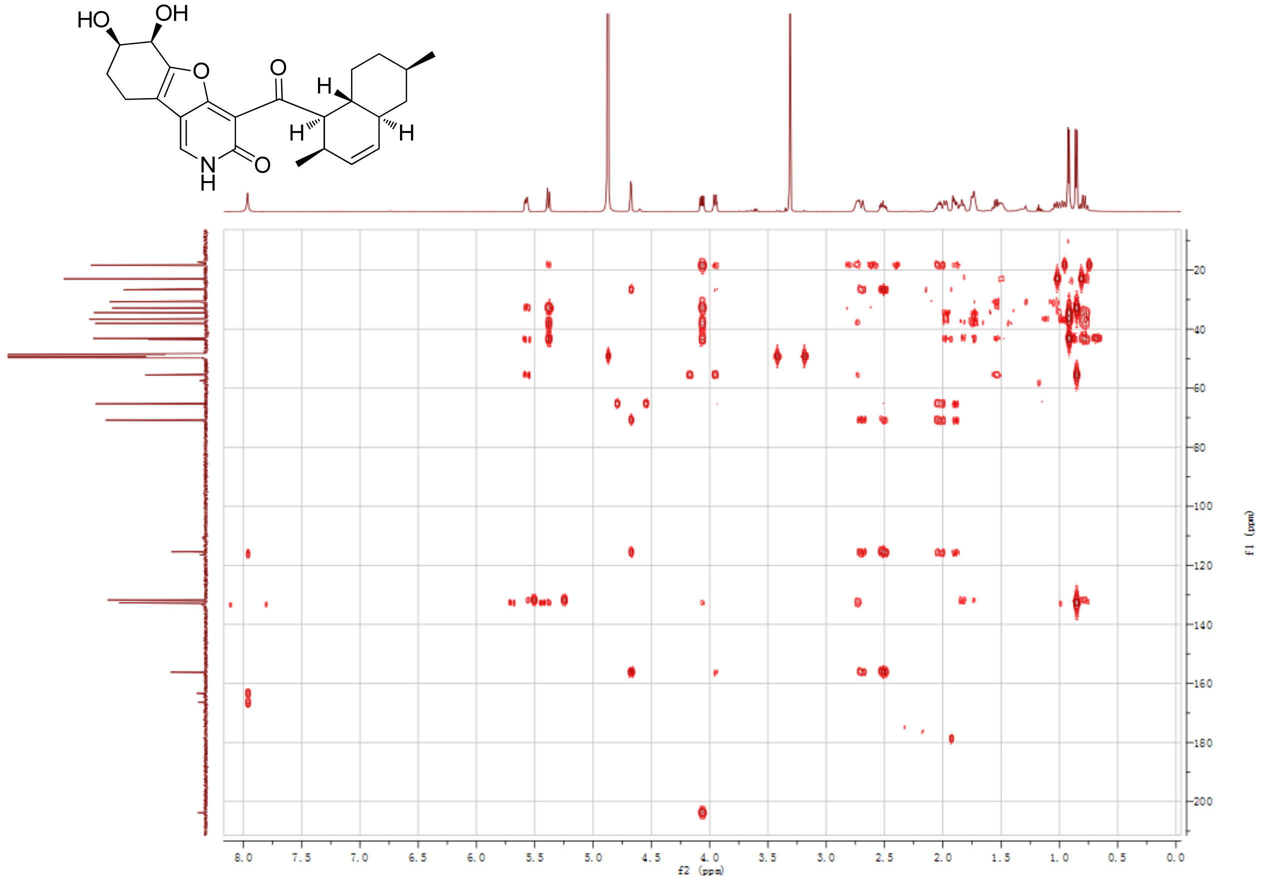

Figure S44. The NOESY spectrum of arthpyrone G **(4)** in CD_3OD

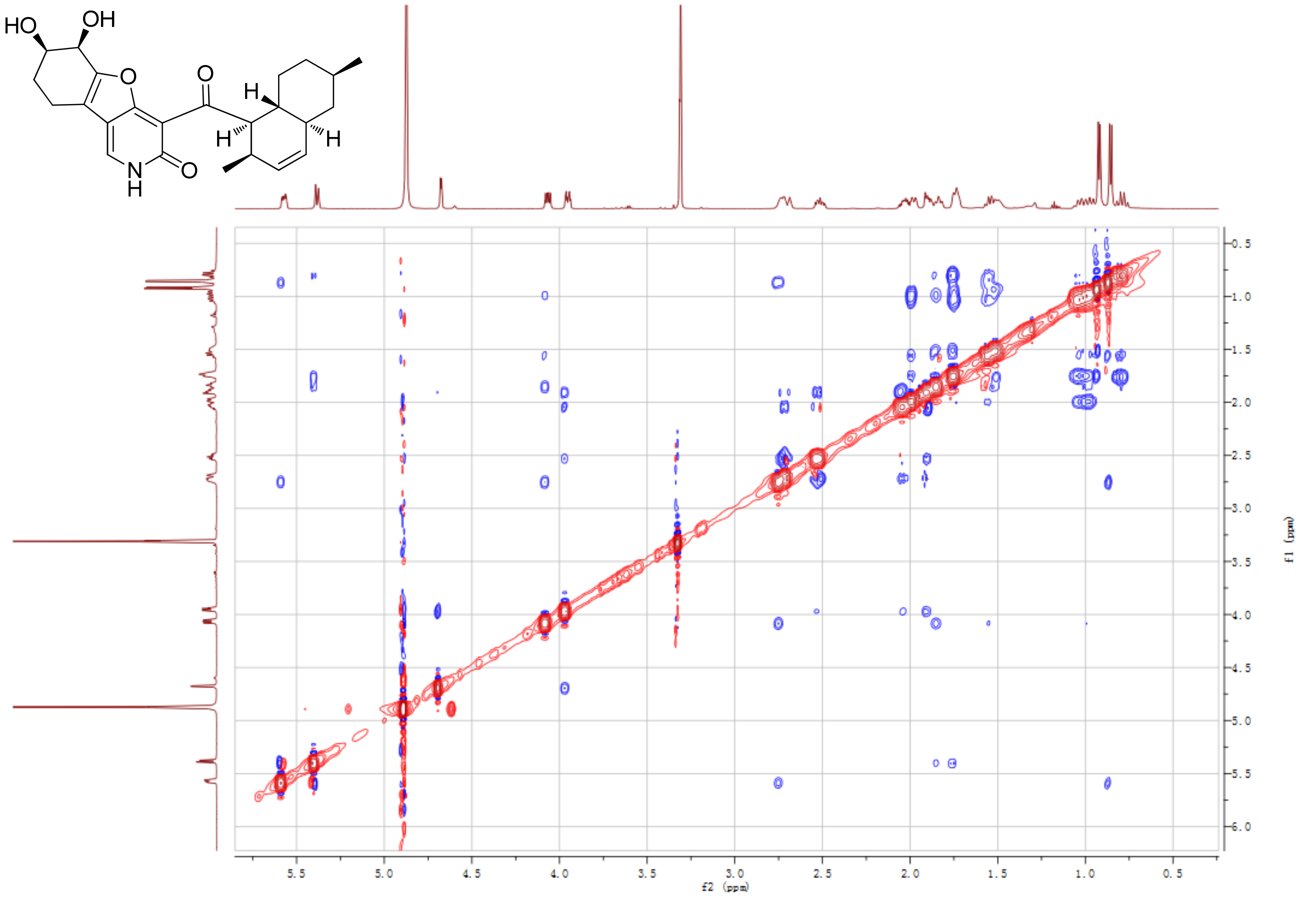

Figure S45. The (-)-HRESIMS spectrum of arthpyrone G **(4)**

**Sample Name**	BJ 70	**Position**	P1-B1	**Instrument Name**	Instrument 1
**User Name**		**Inj Vol**	2	**InjPosition**	
**Sample Type**	Sample	**IRM Calibration Status**	Success	**Data Filename**	BJ 70.d
**ACQ Method**	wss-isocratic elution-Negative.m	**Comment**		**Acquired Time**	11/23/2017 9:31:34 AM

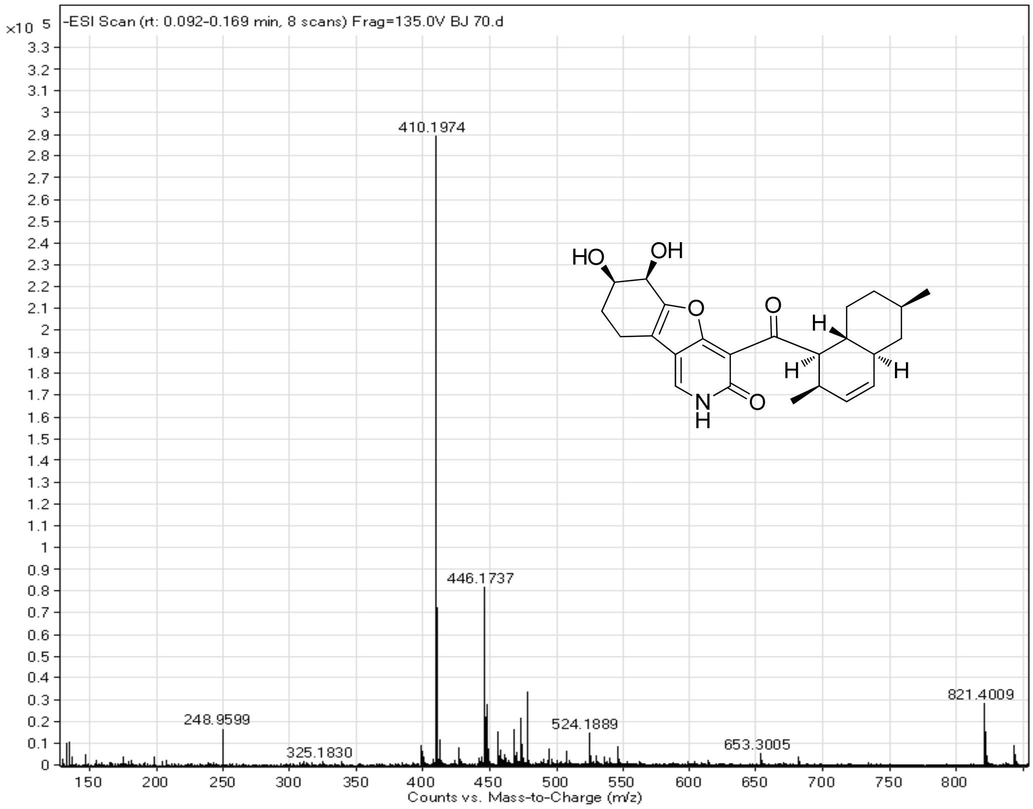

Figure S46. The ^{1}H-NMR spectrum of arthpyrone H **(5)** in CD_3OD

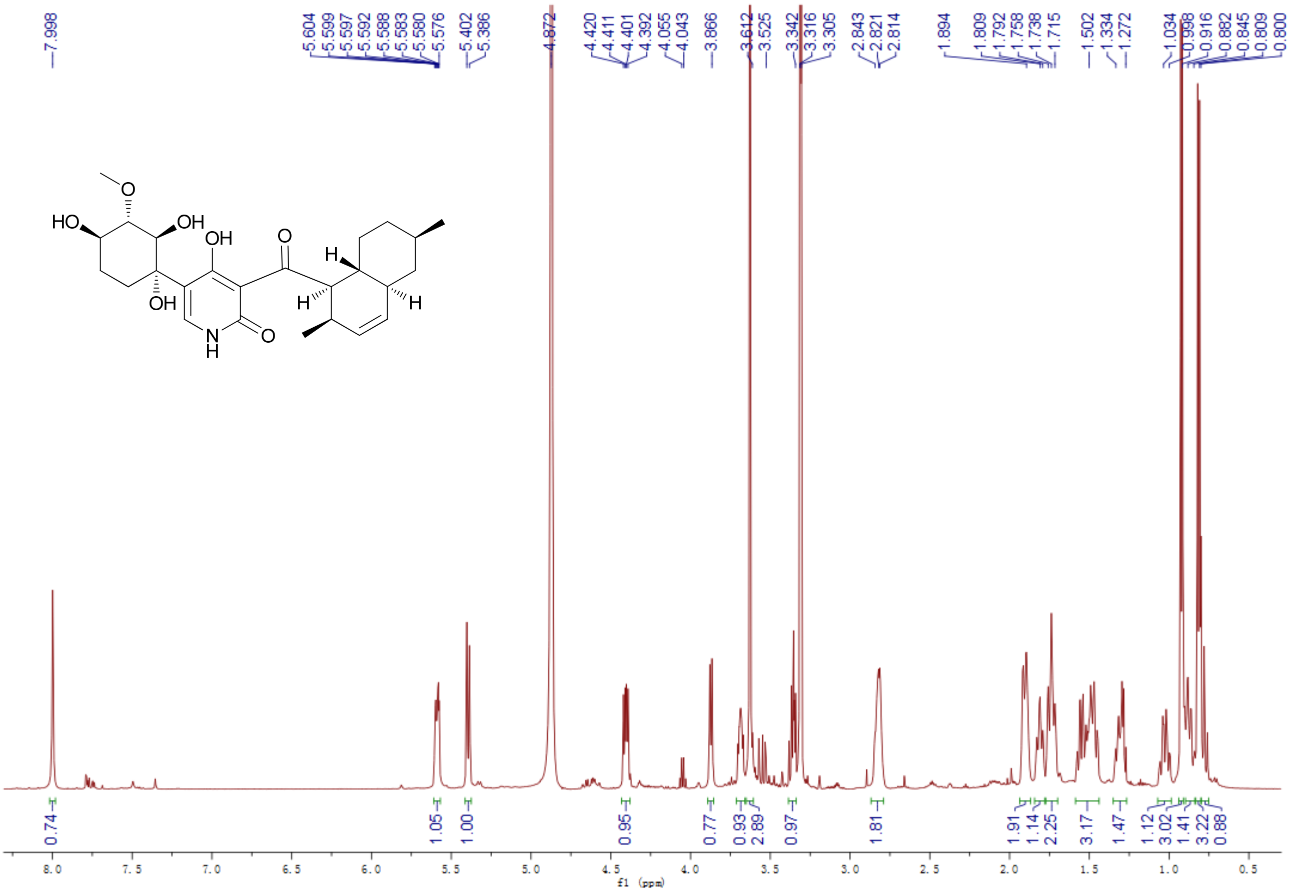

Figure S47. The ^{13}C-NMR spectrum of arthpyrone H **(5)** in CD_3OD

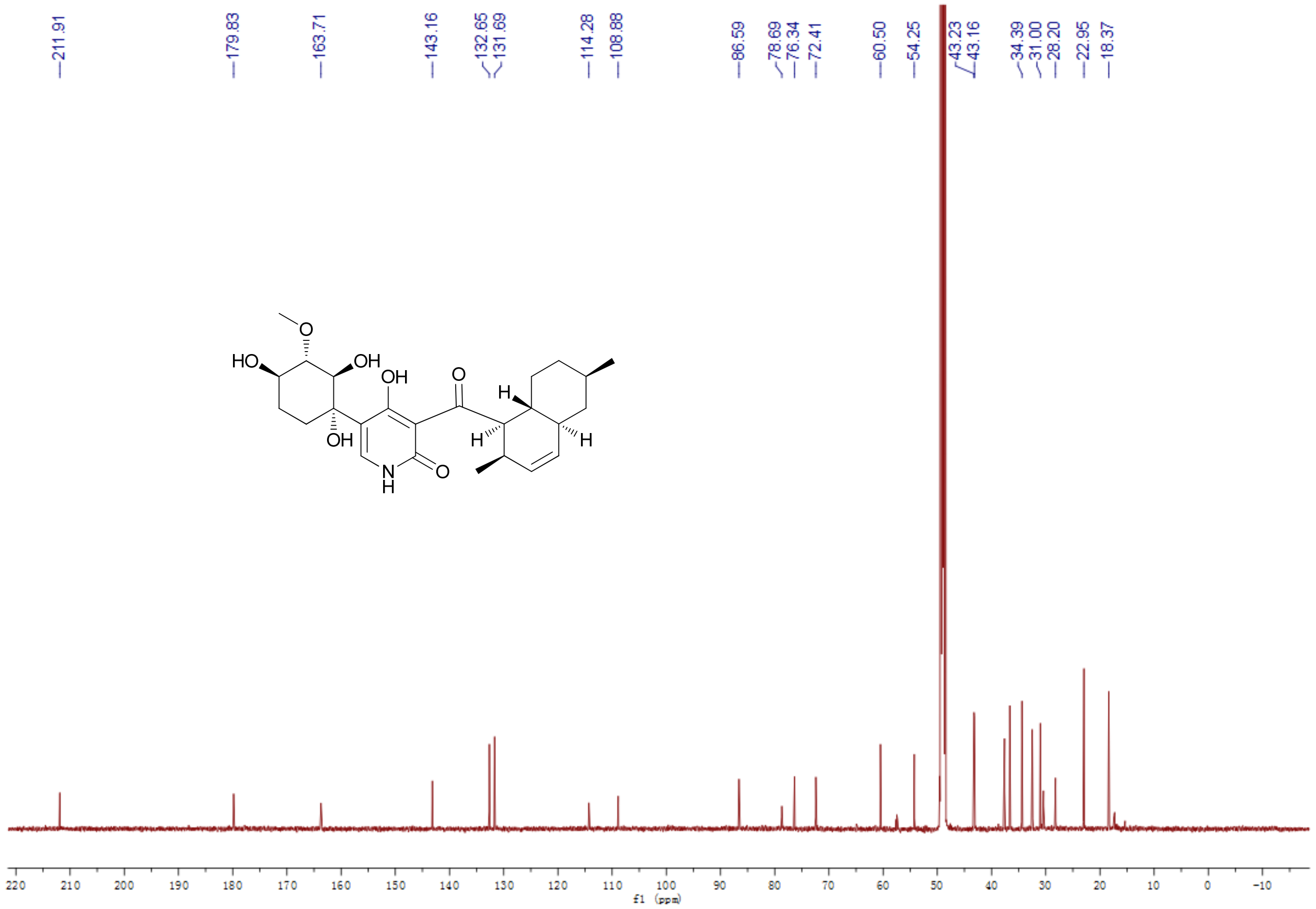

Figure S48. The DEPT 135 spectrum of arthpyrone H **(5)** in CD_3OD

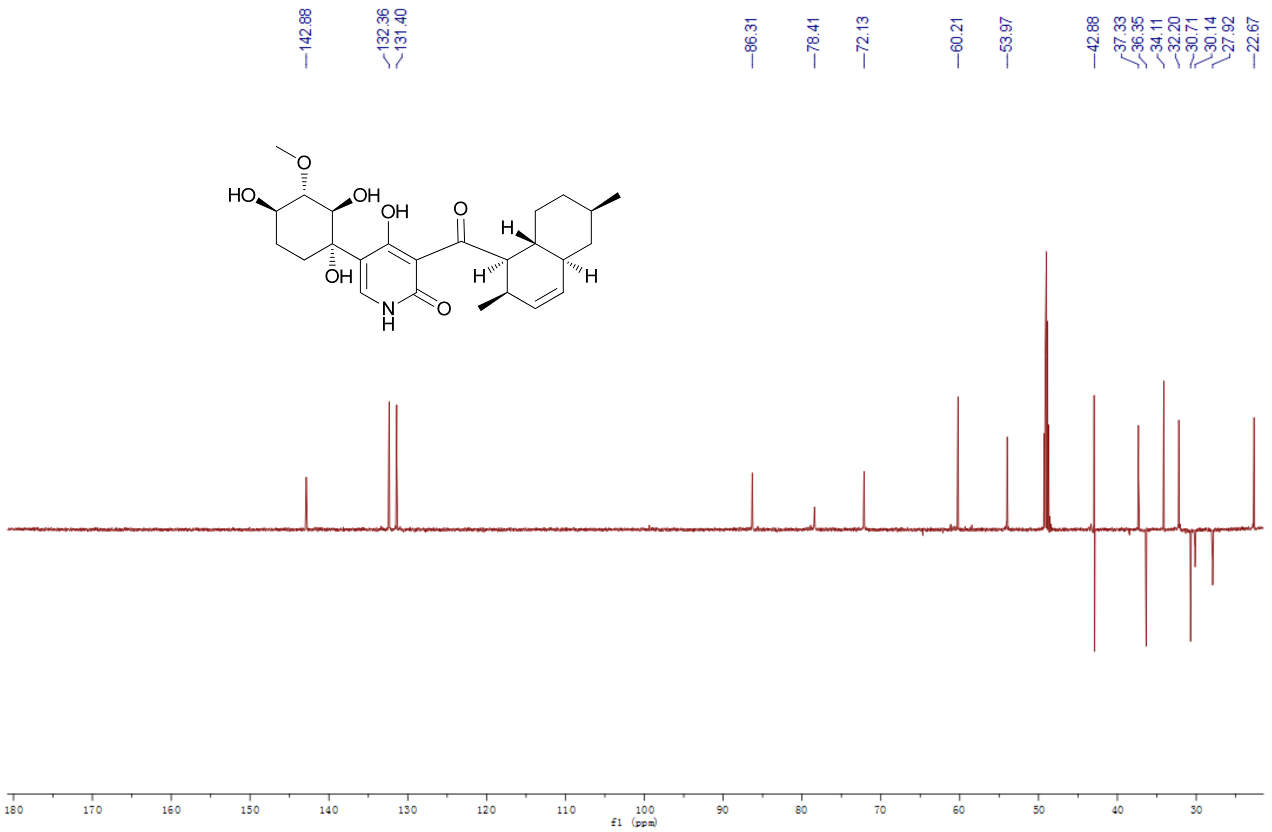

Figure S49. The HSQC spectrum of arthpyrone H **(5)** in CD_3OD

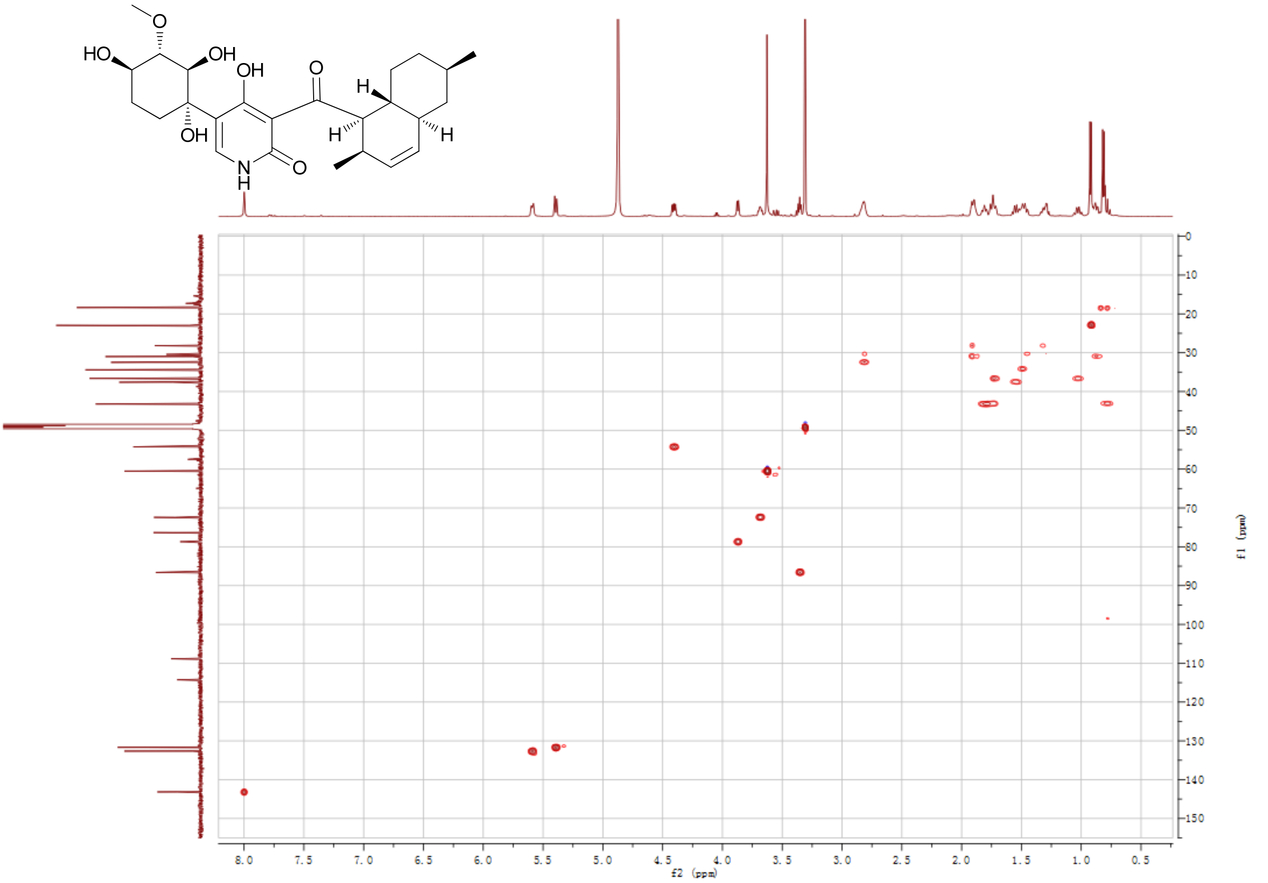

Figure S50. The ^{1}H-^{1}H COSY spectrum of arthpyrone H **(5)** inCD_3OD

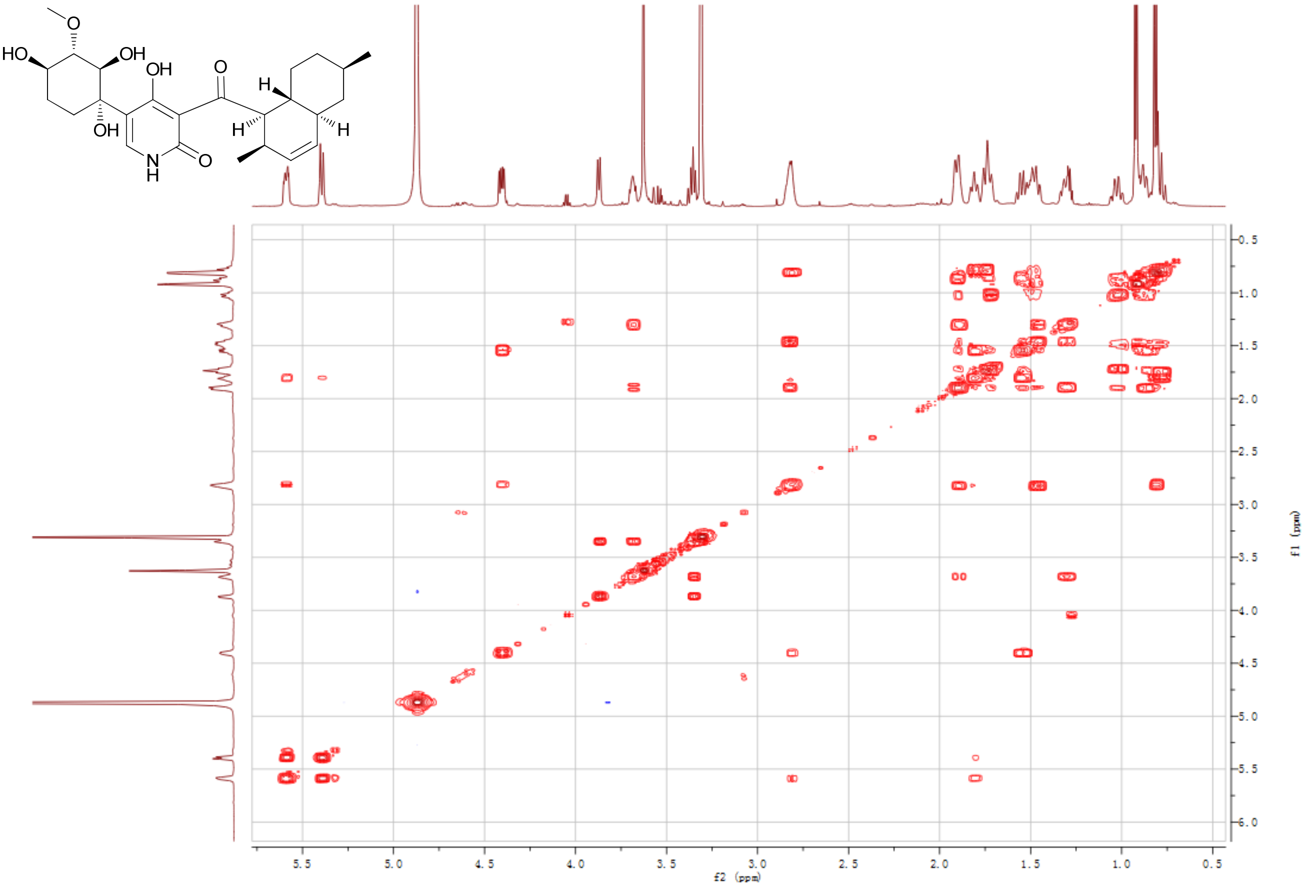

Figure S51. The HMBC spectrum of arthpyrone H **(5)** in CD_3OD

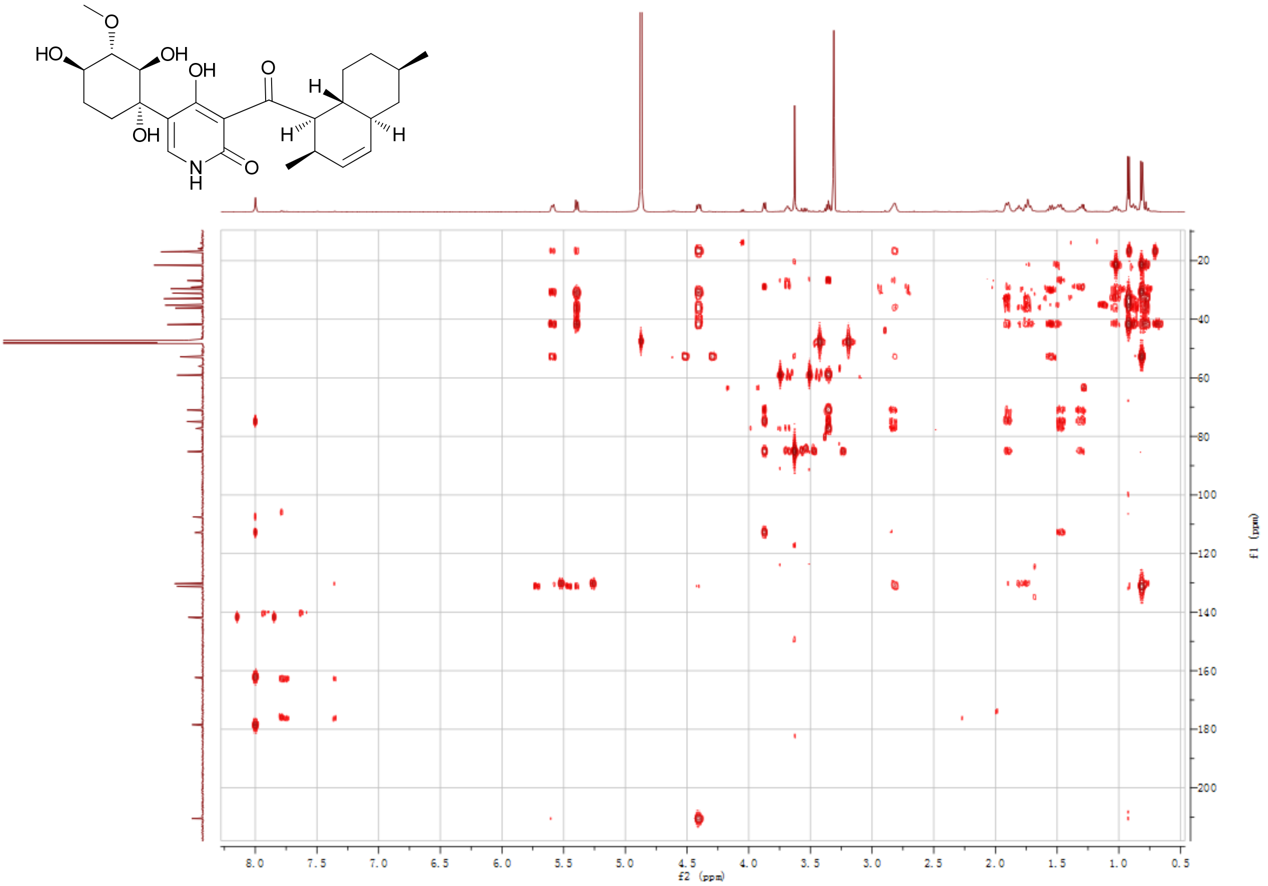

Figure S52. The NOESY spectrum of arthpyrone H **(5)** in CD_3OD

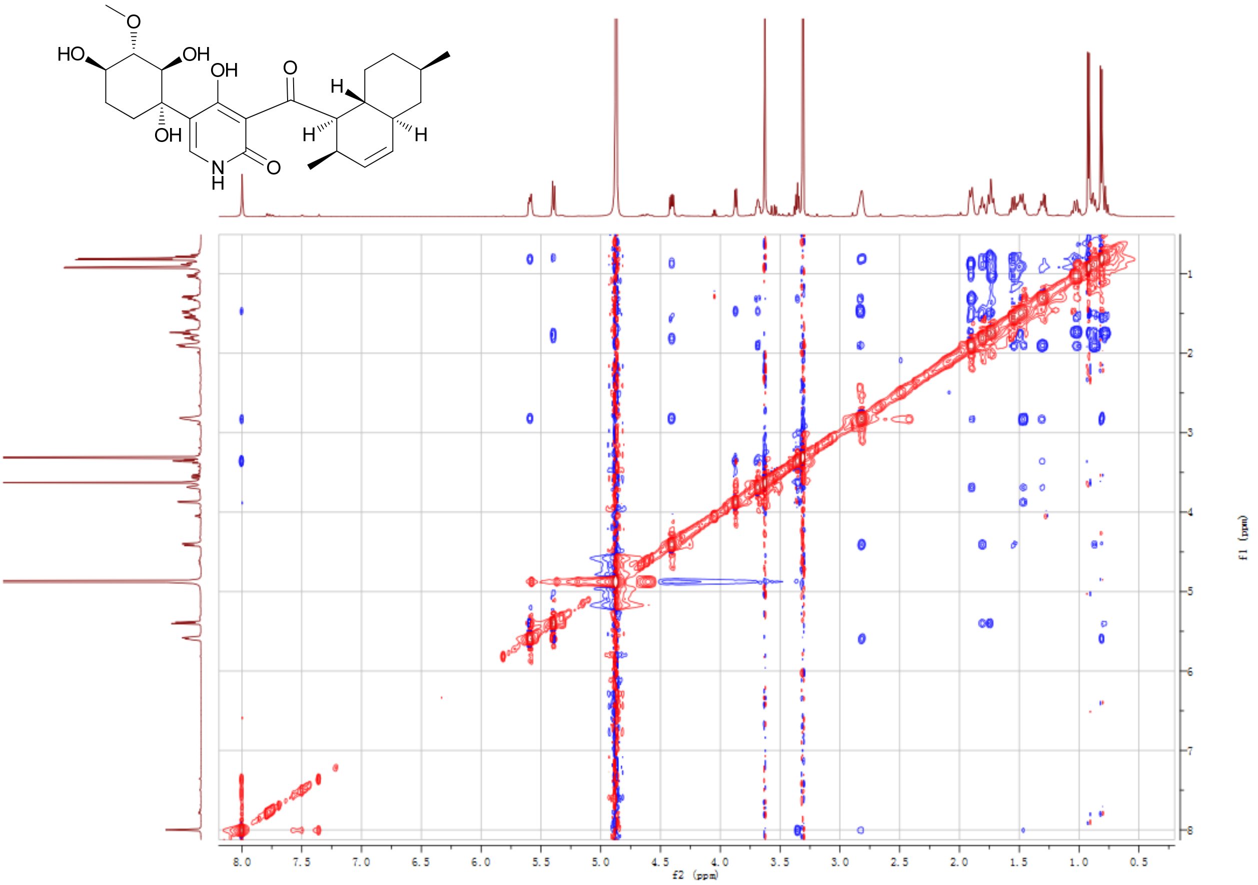

Figure S53. The (-)-HRESIMS spectrum of arthpyrone H **(5)**

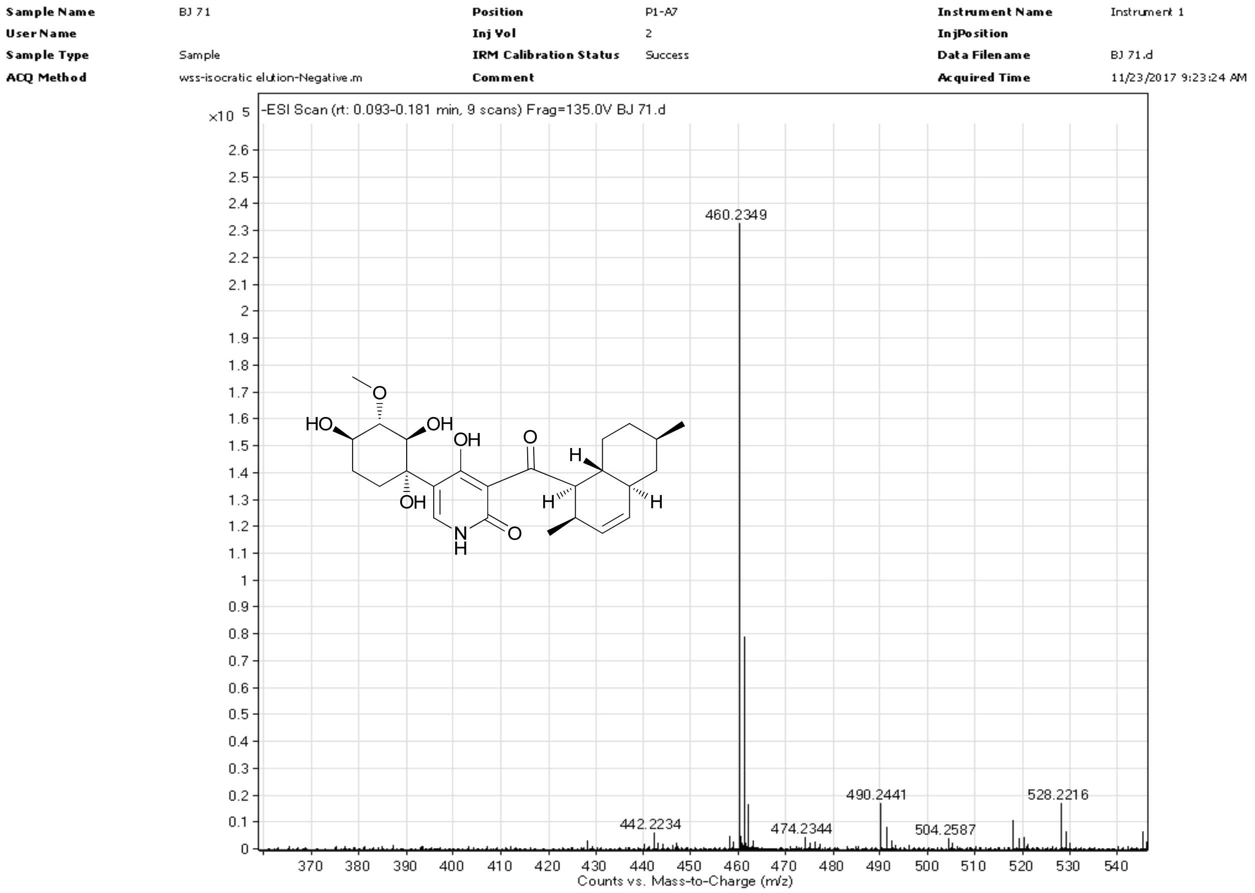

Figure S54. The ^{1}H-NMR spectrum of arthpyrone I **(6)** in CD_3OD

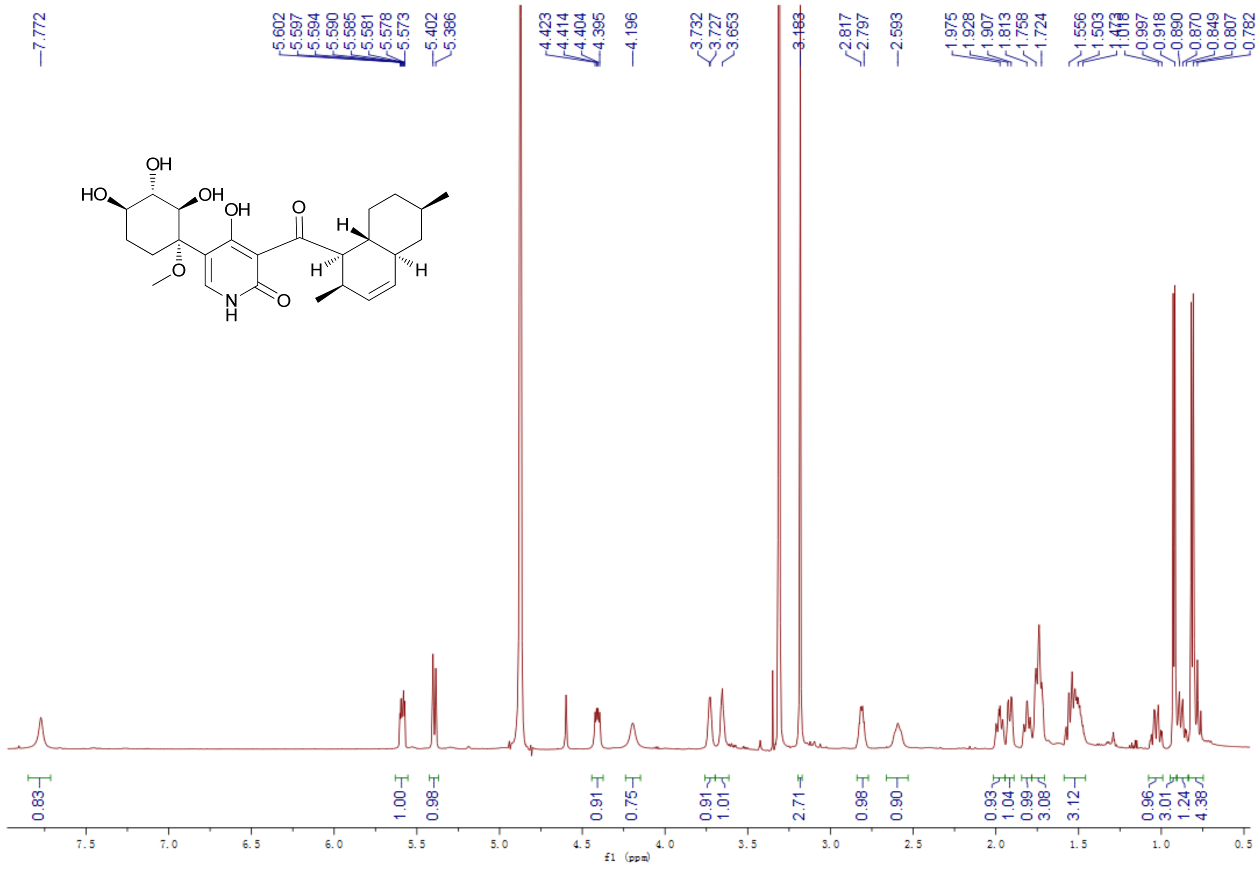

Figure S55. The ^{13}C-NMR spectrum of arthpyrone I **(6)** in CD_3OD

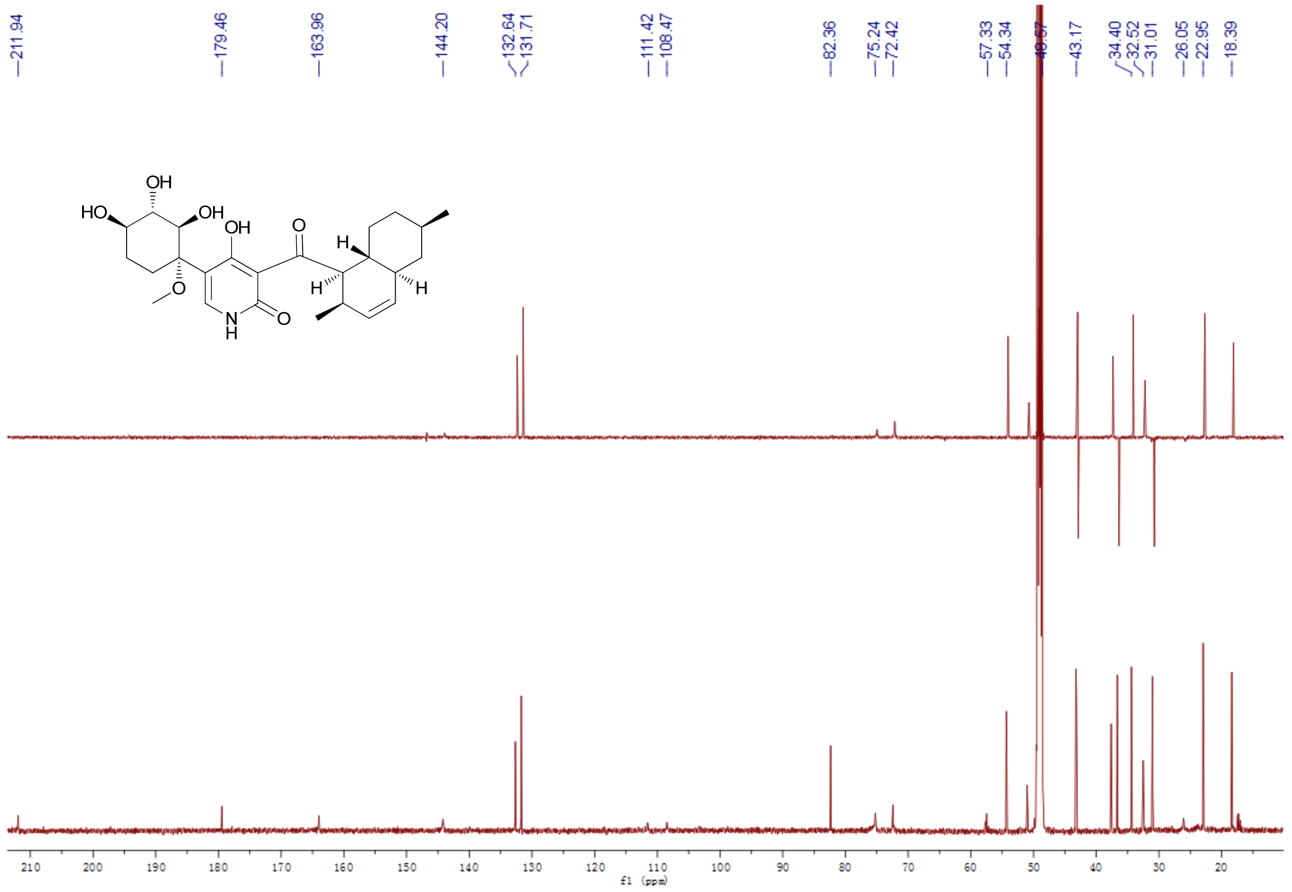

Figure S56. The HSQC spectrum of arthpyrone I **(6)** in CD_3OD

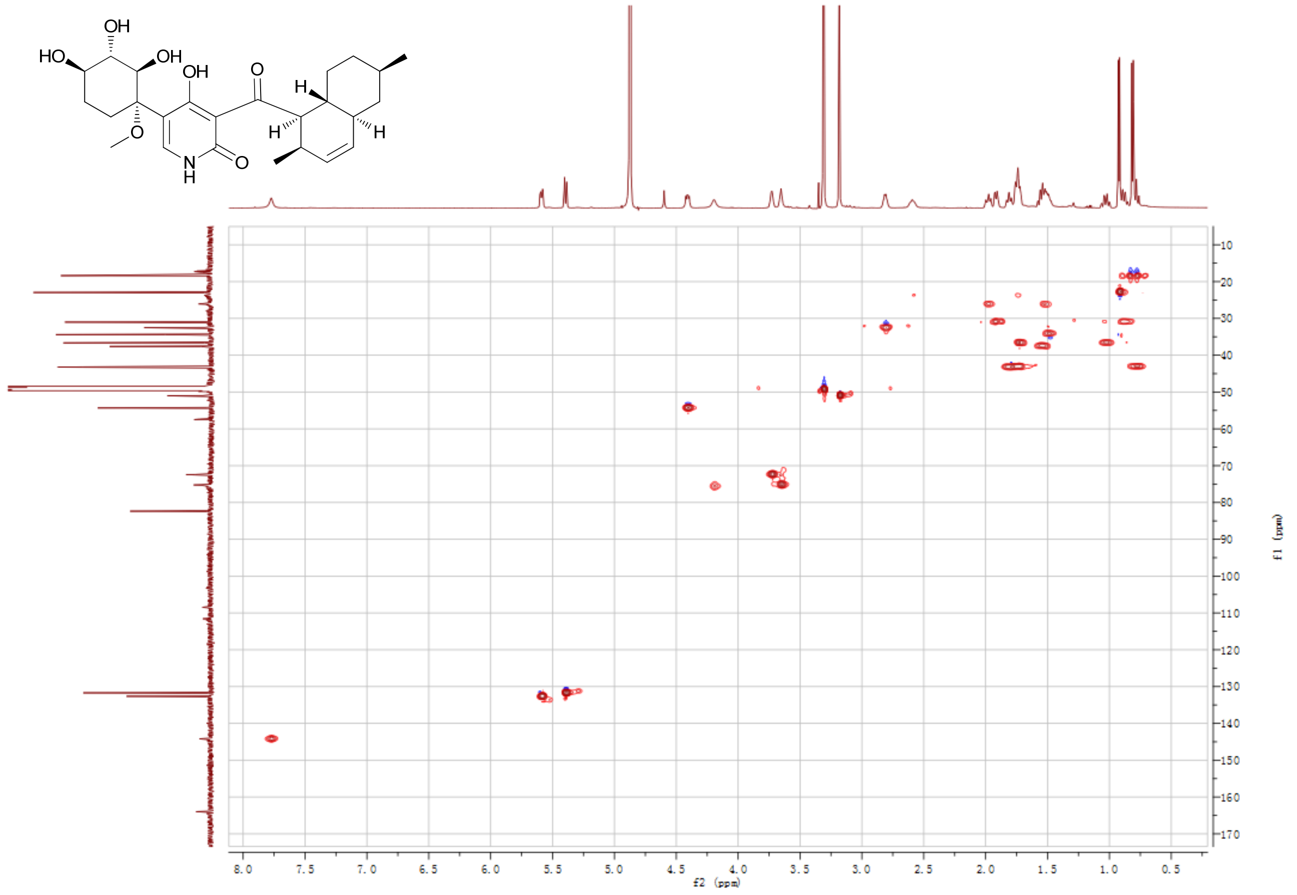

Figure S57. The ^{1}H-^{1}H COSY spectrum of arthpyrone I **(6)** inCD$_3$OD

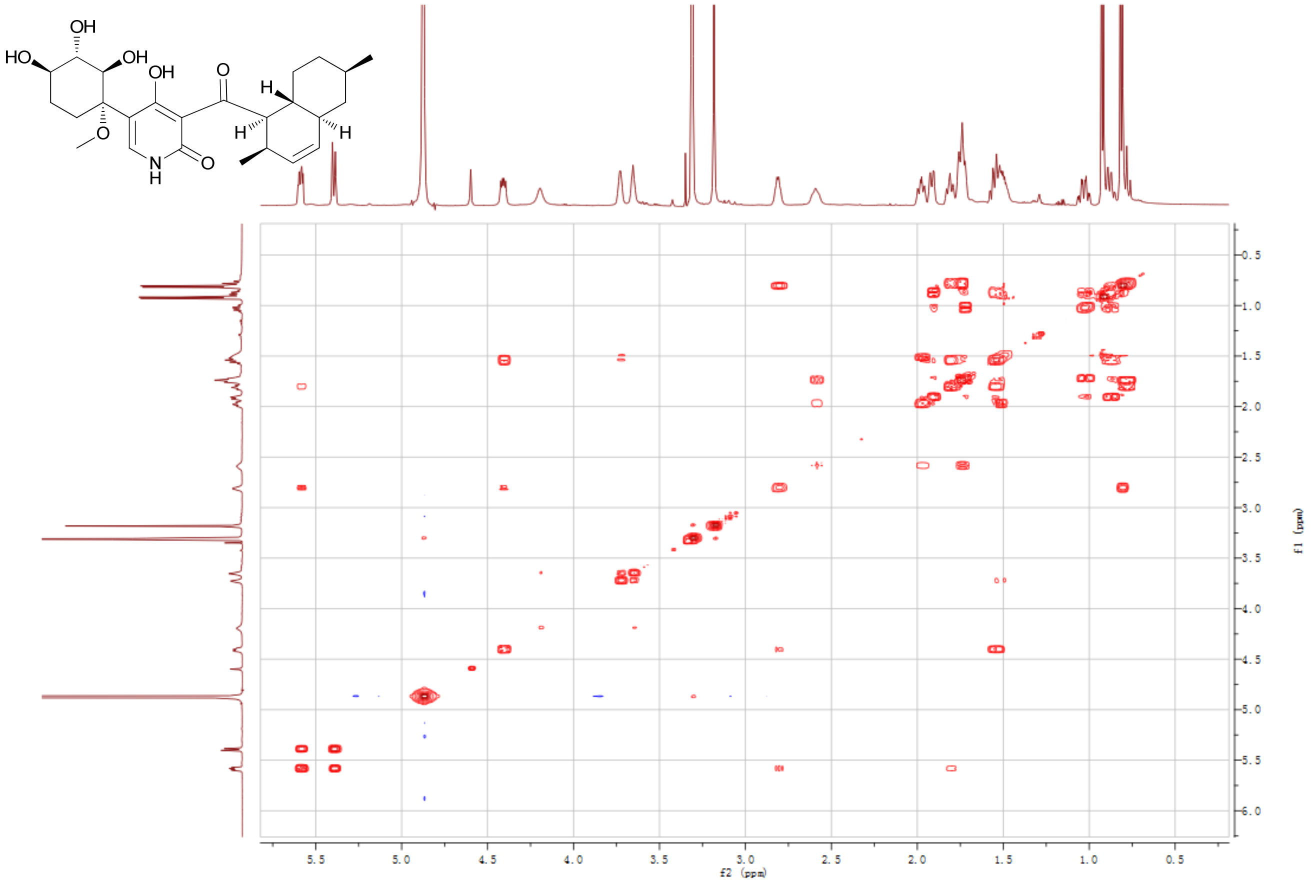

Figure S58. The HMBC spectrum of arthpyrone I **(6)** in CD$_3$OD

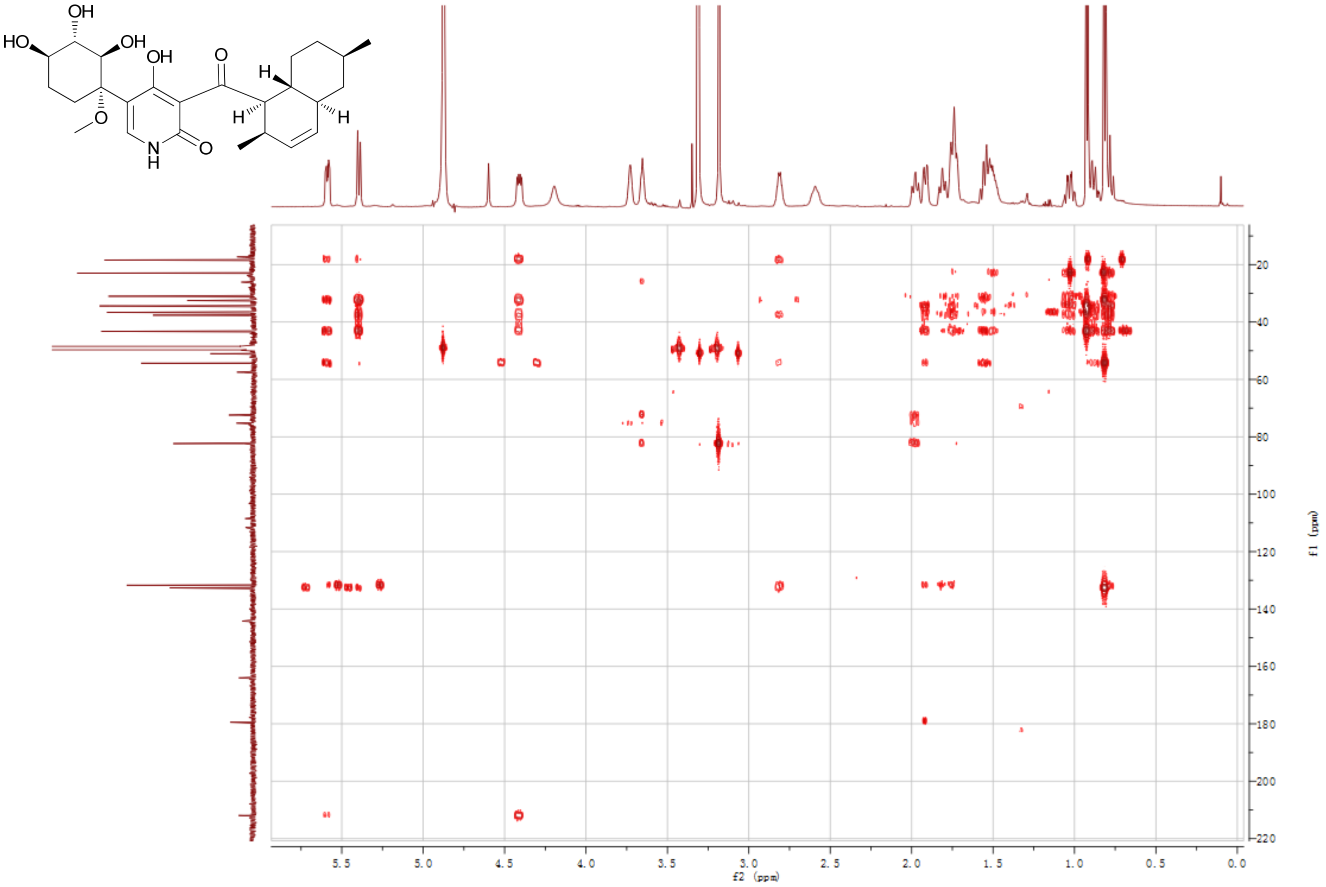

Figure S59. The NOESY spectrum of arthpyrone I **(6)** in CD_3OD

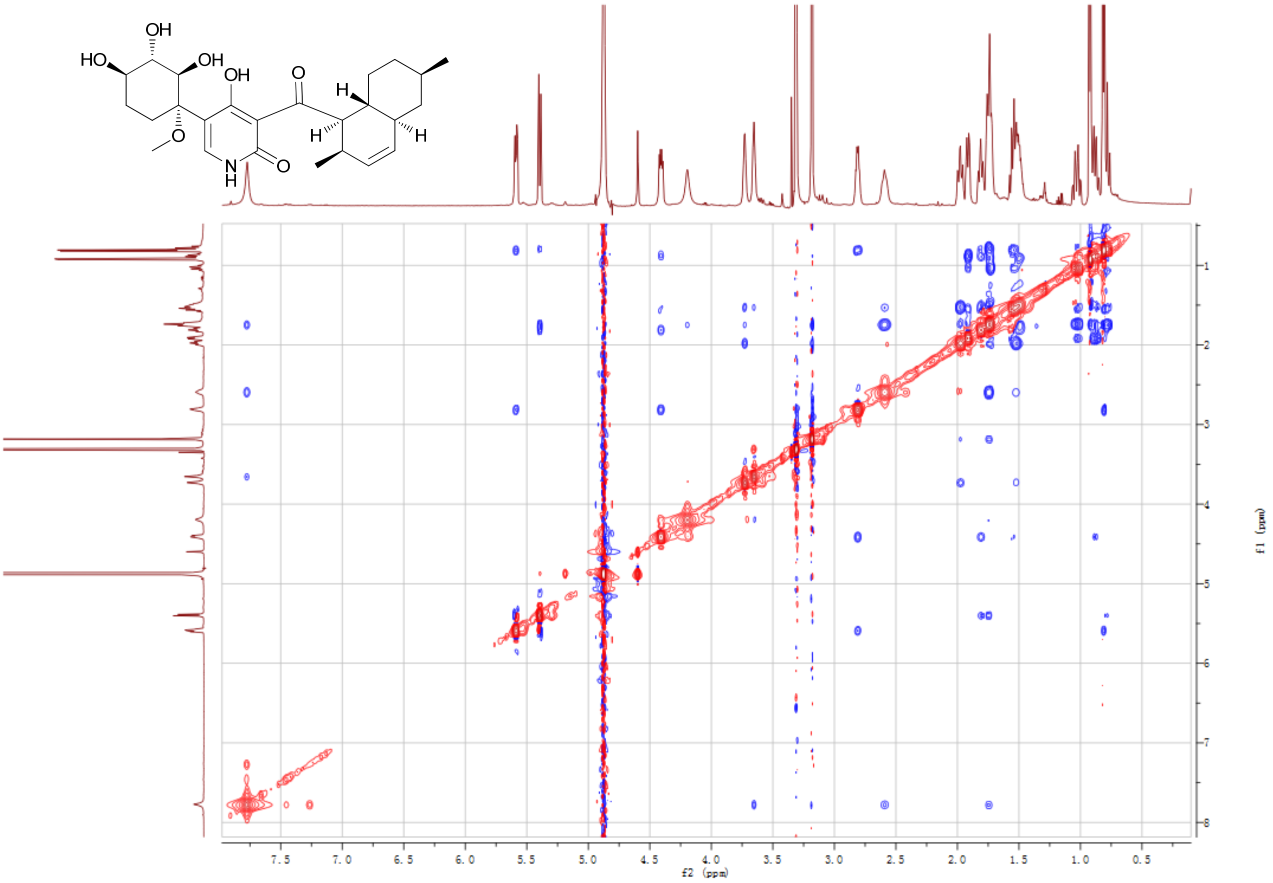

Figure S60. The (-)-HRESIMS spectrum of arthpyrone I **(6)**

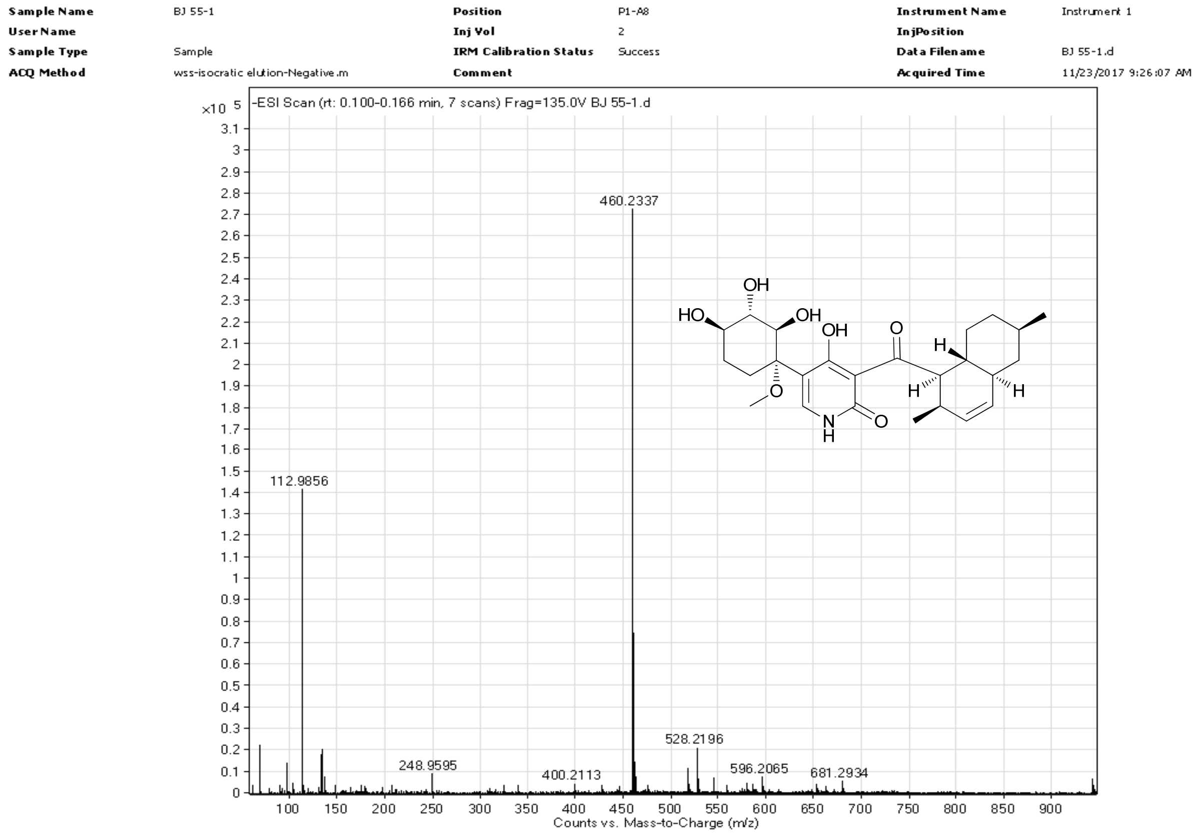

Figure S61. The ^{1}H-NMR spectrum of arthpyrone J **(7)** in CD_3OD

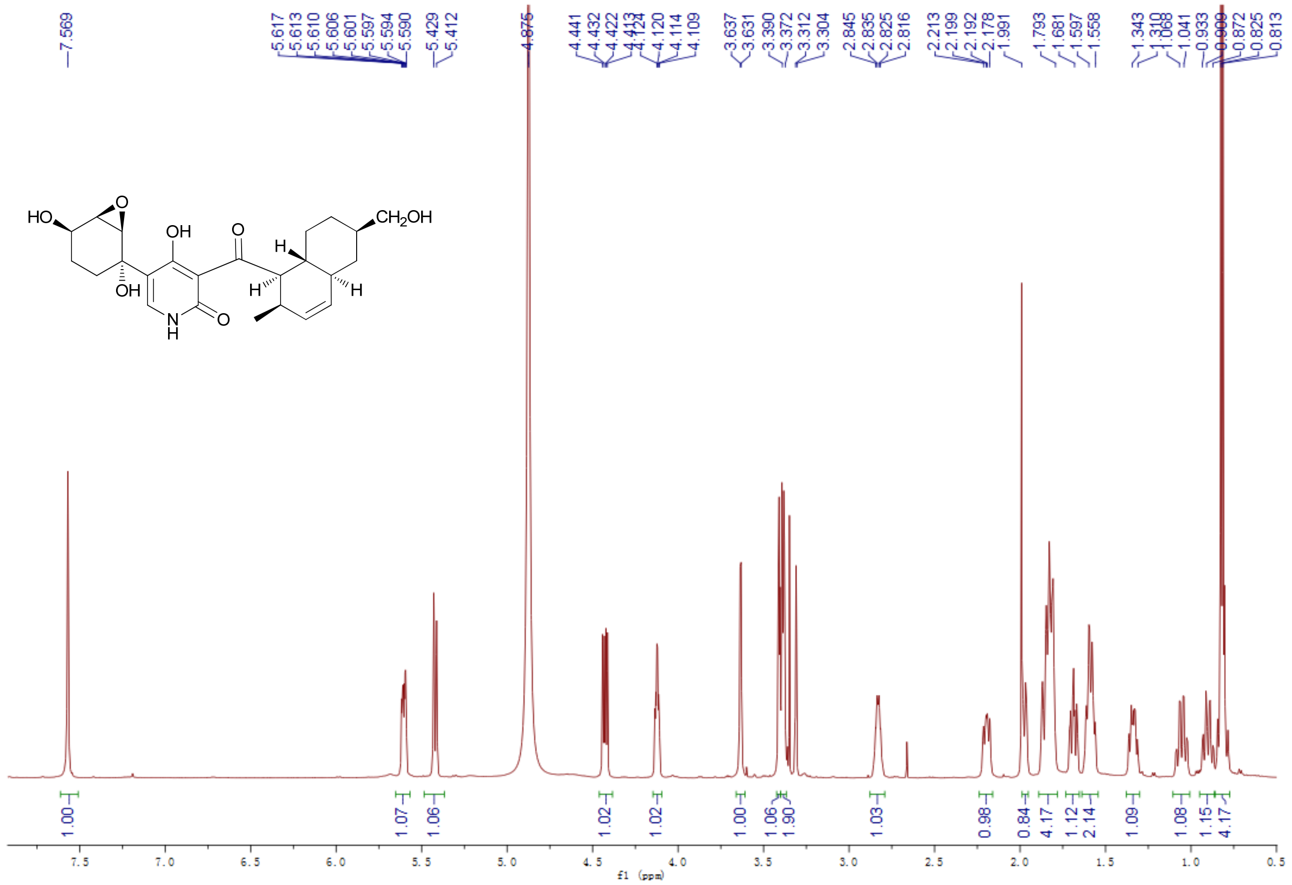

Figure S62. The ^{13}C-NMR spectrum of arthpyrone J **(7)** in CD_3OD

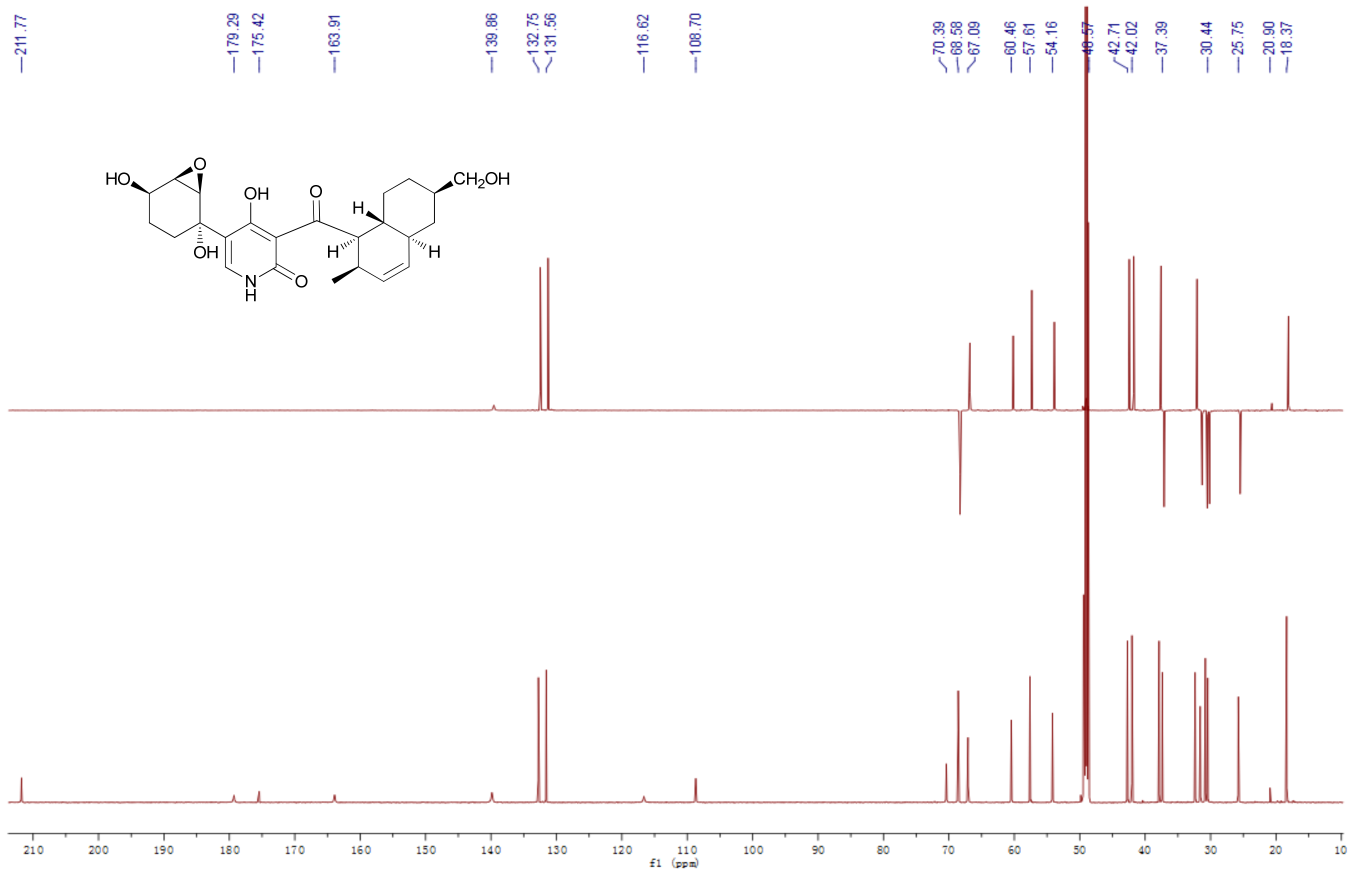

Figure S63. The HSQC spectrum of arthpyrone J **(7)** in CD_3OD

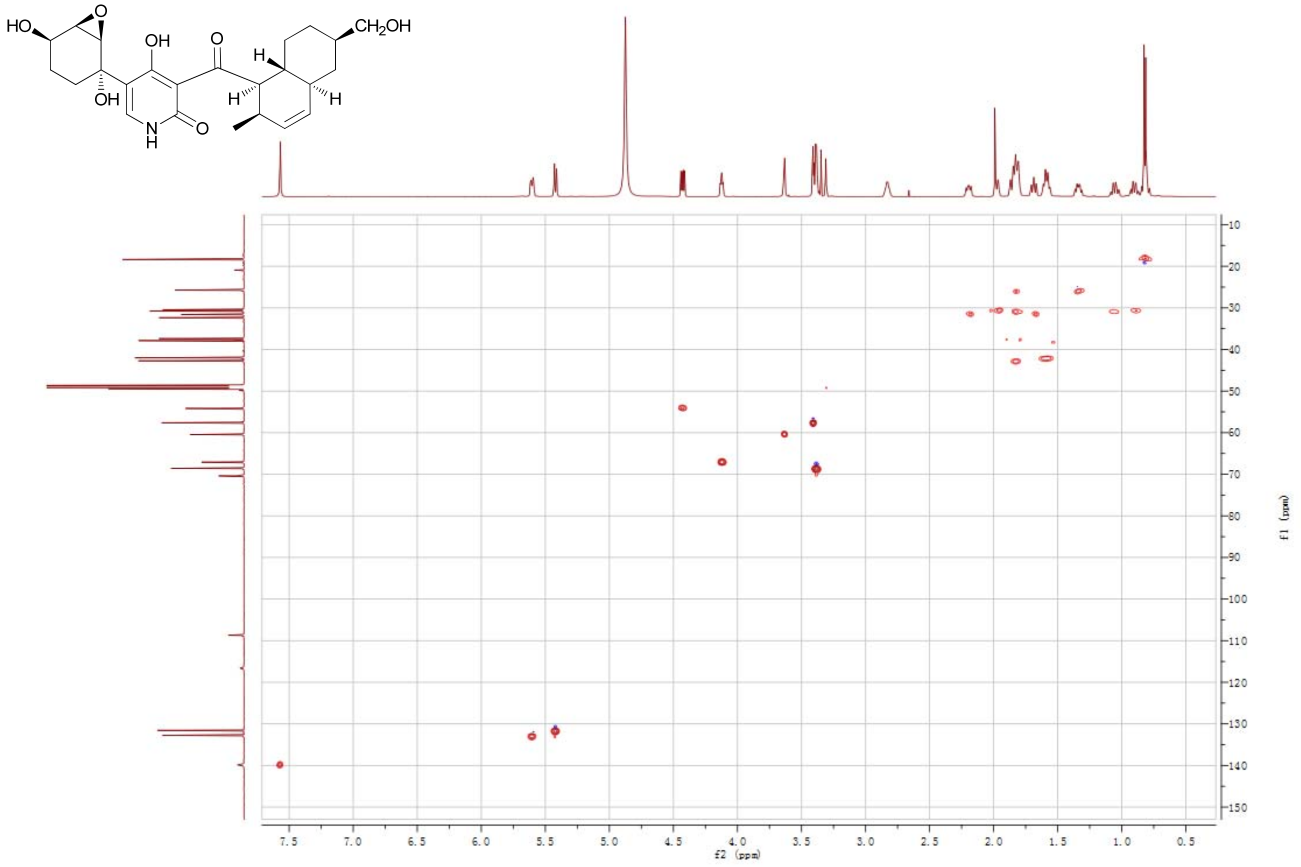

Figure S64. The ^1H-^1H COSY spectrum of arthpyrone J **(7)** in CD_3OD

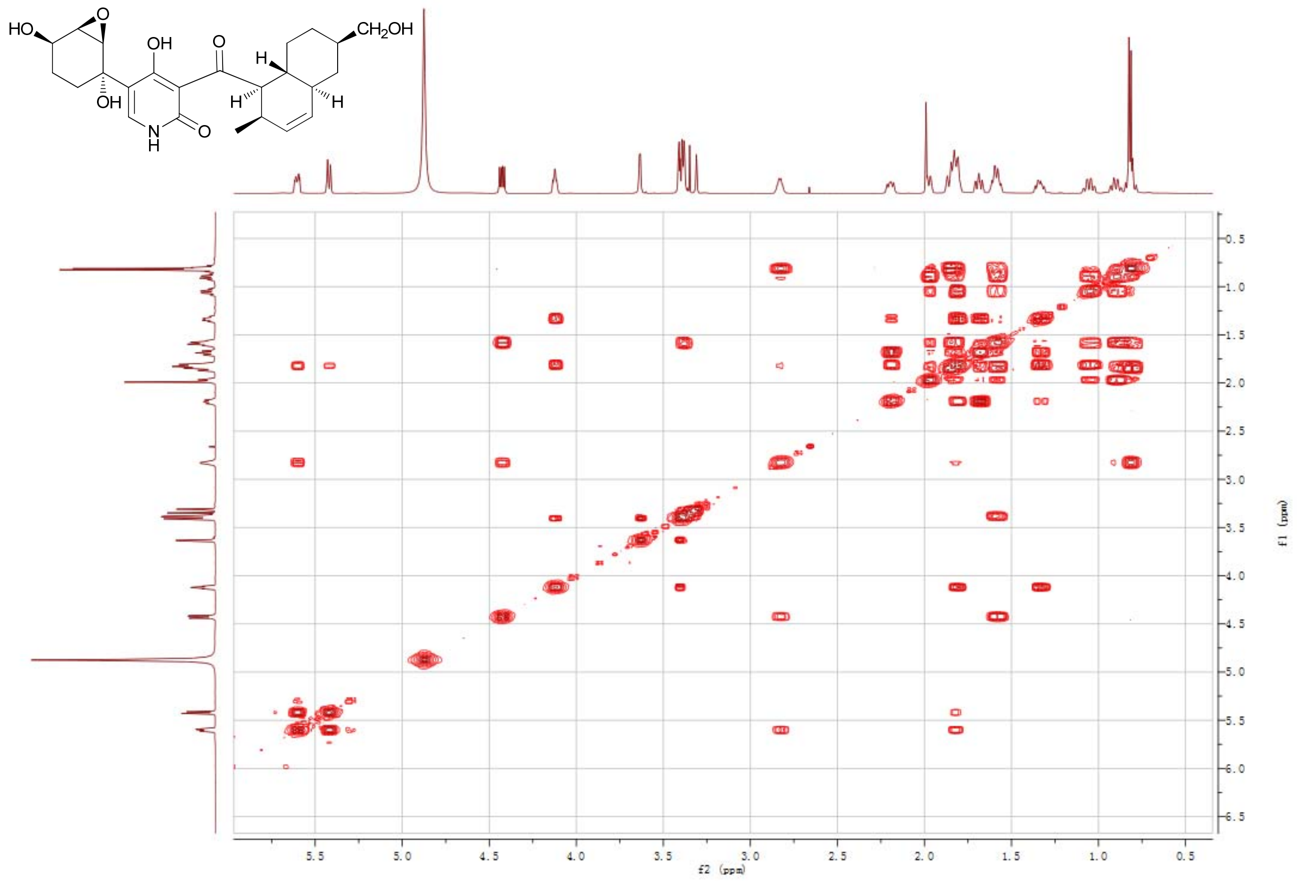

Figure S65. The HMBC spectrum of arthpyrone J **(7)** in CD_3OD

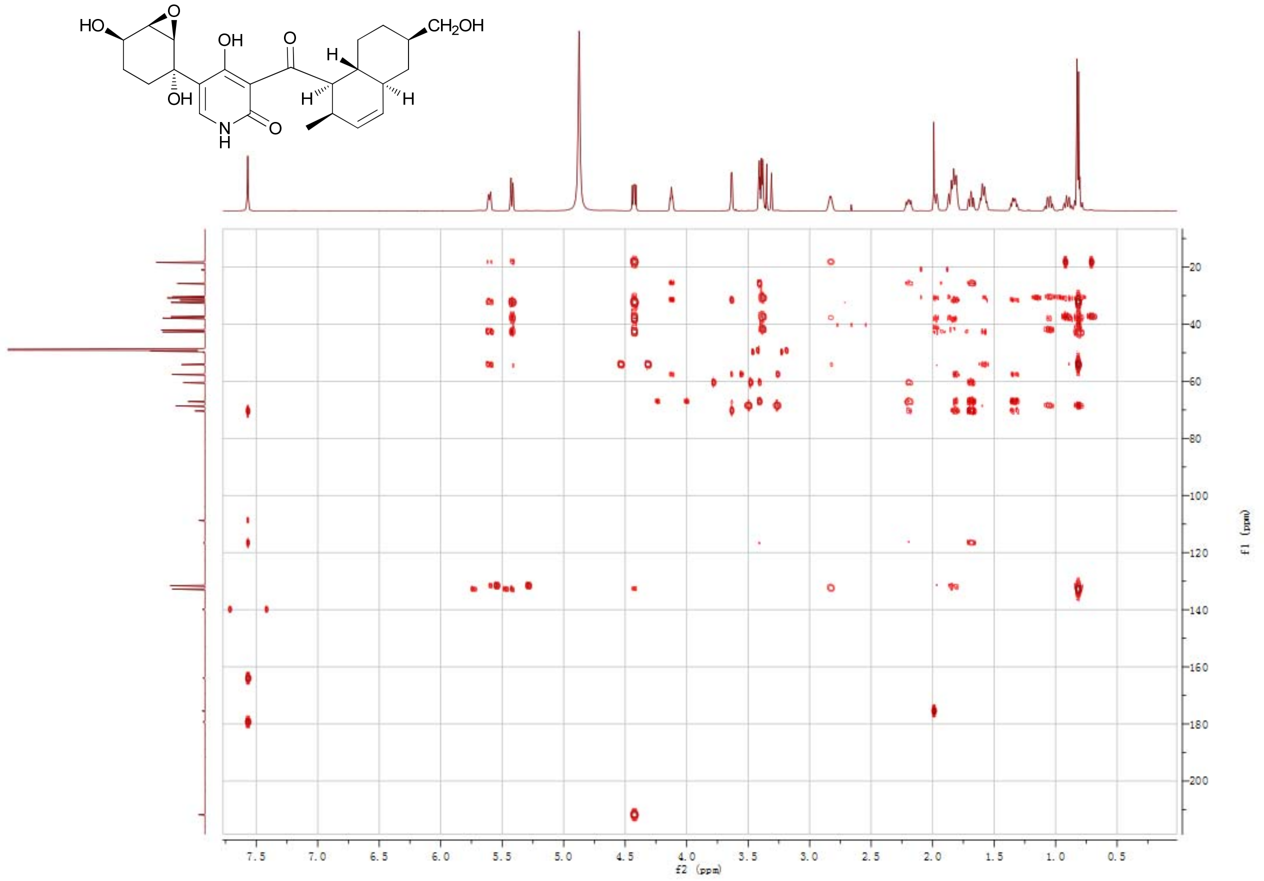

Figure S66. The NOESY spectrum of arthpyrone J **(7)** in CD_3OD

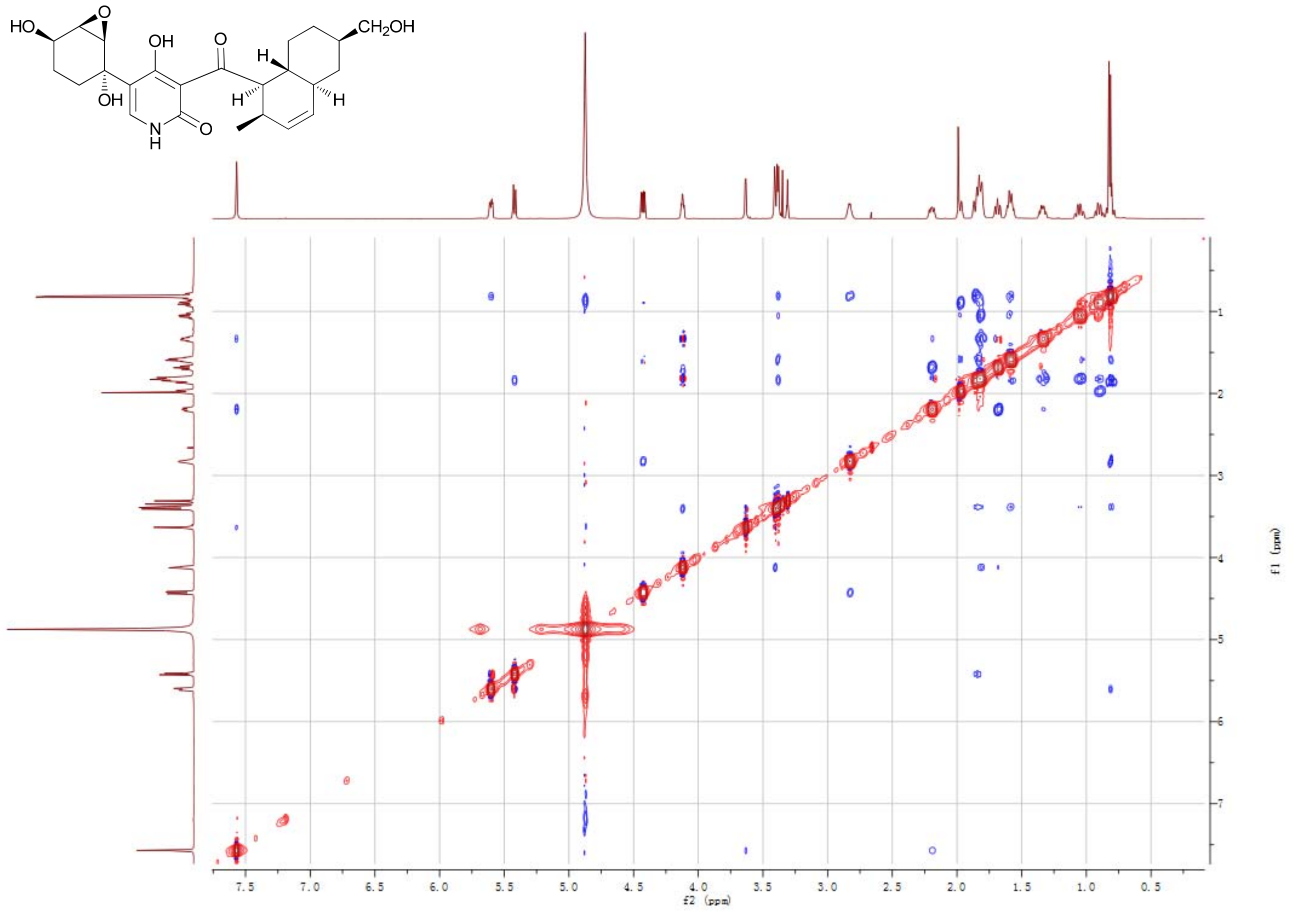

Figure S67. The (-)-HRESIMS spectrum of arthpyrone J **(7)**

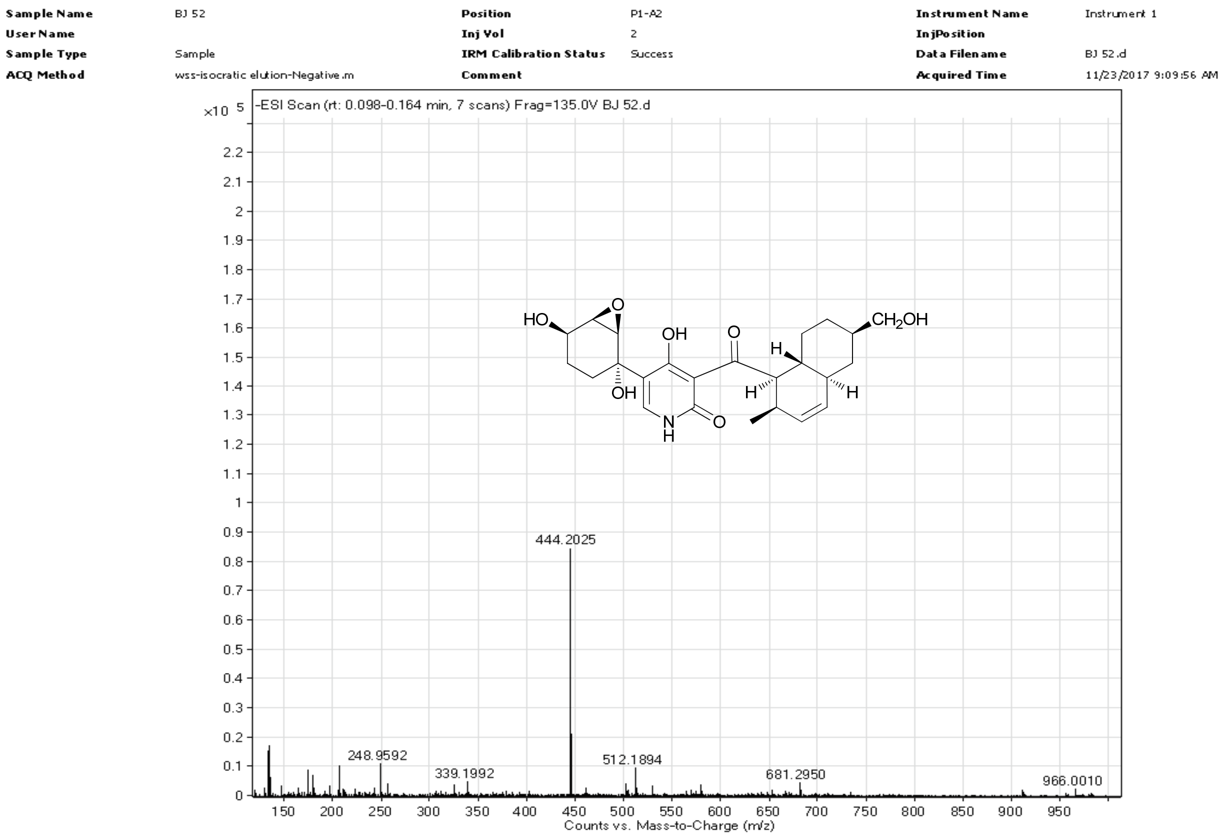

Figure S68. The ^{1}H-NMR spectrum of arthpyrone K **(8)** in CD_3OD

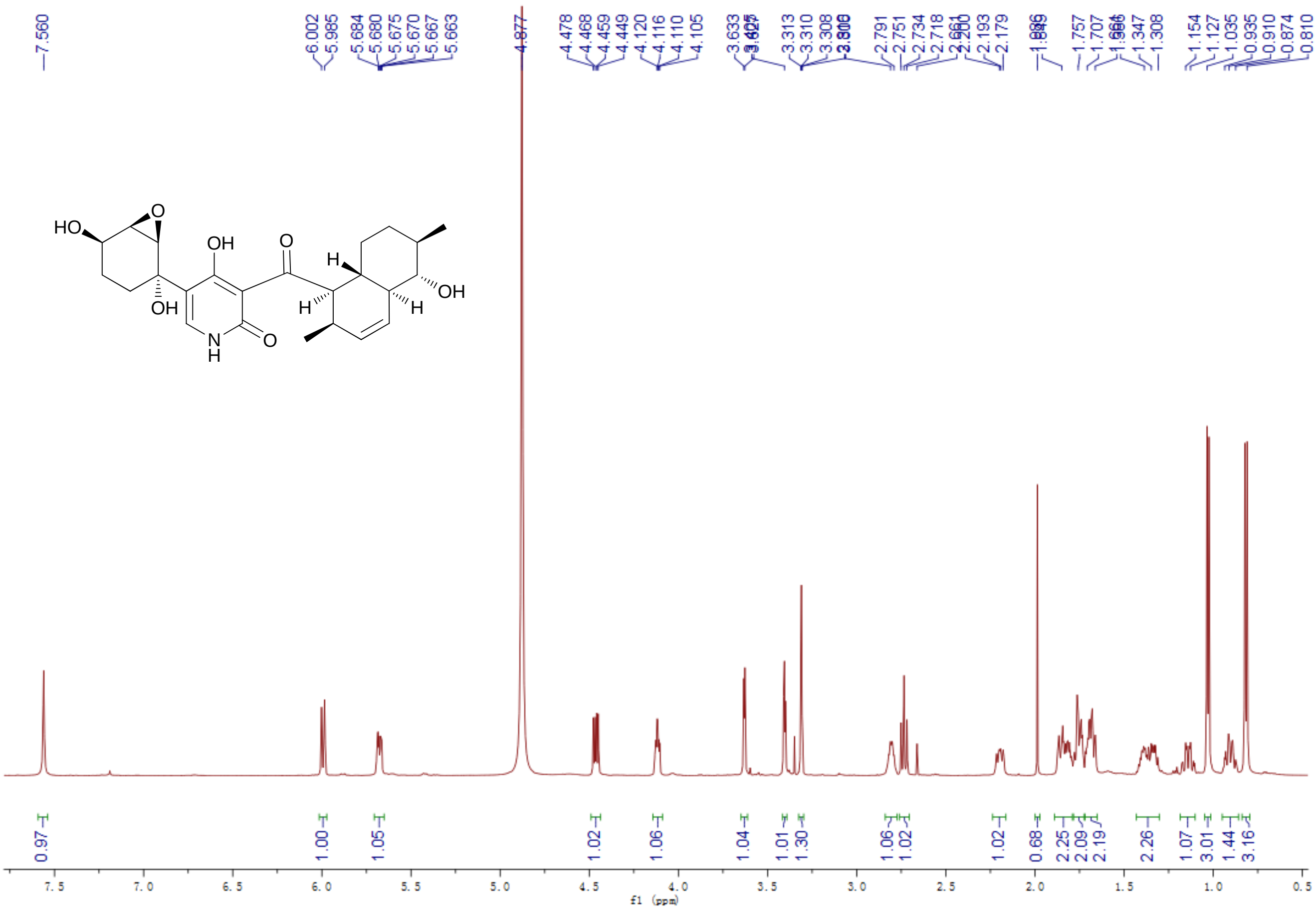

Figure S69. The ^{13}C-NMR spectrum of arthpyrone K **(8)** in CD_3OD

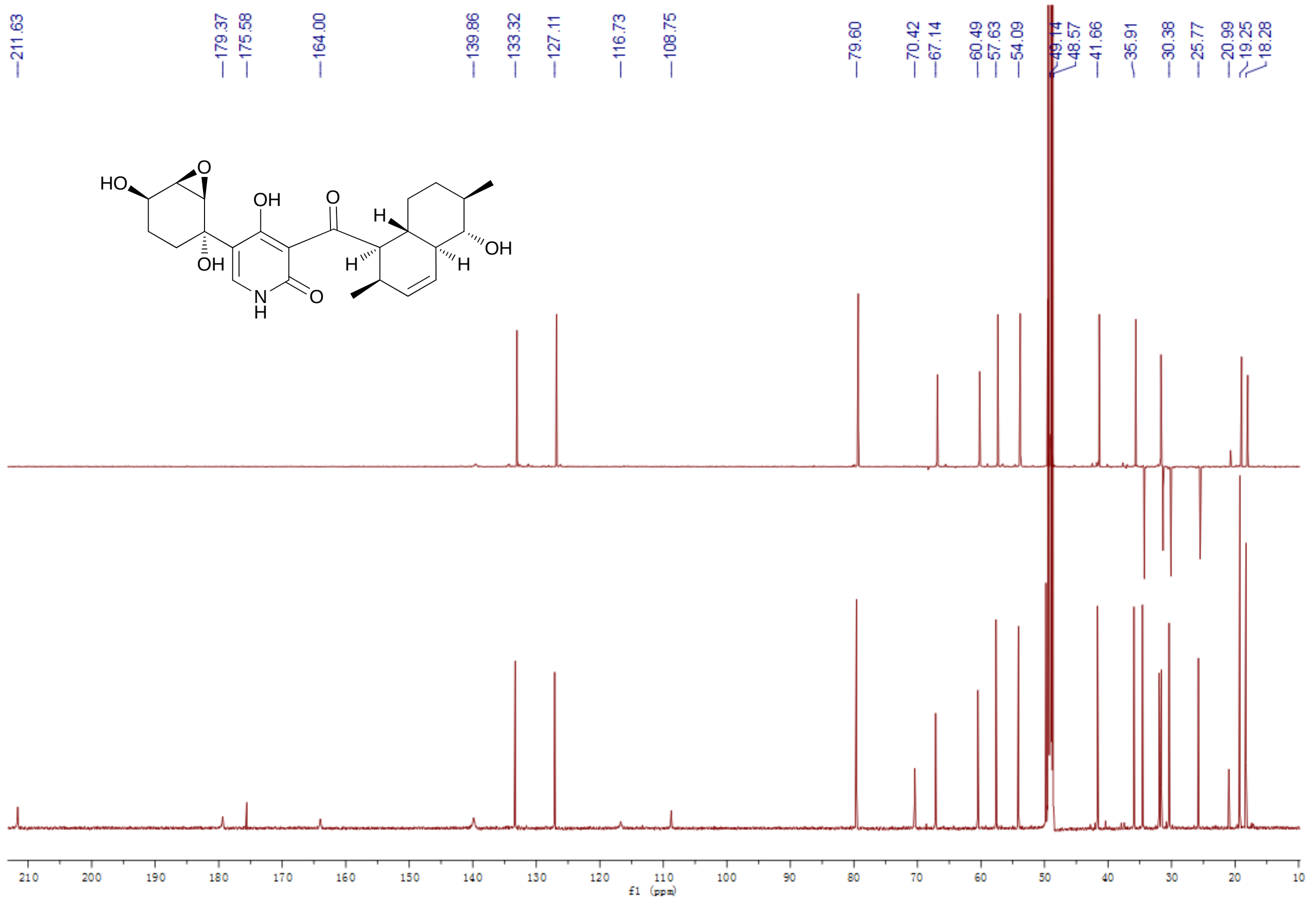

Figure S70. The HSQC spectrum of arthpyrone K **(8)** in CD_3OD

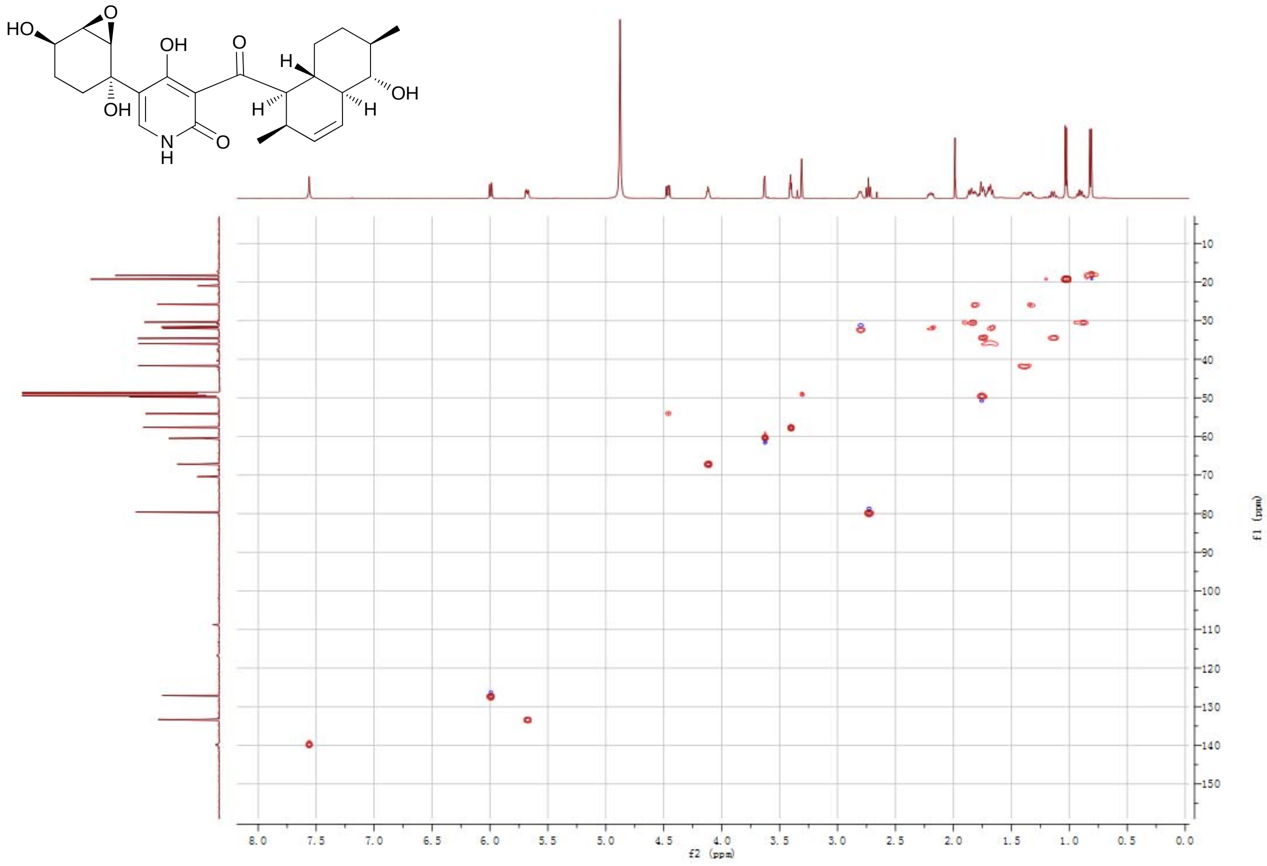

Figure S71. The ^{1}H-^{1}H COSY spectrum of arthpyrone K **(8)** inCD$_3$OD

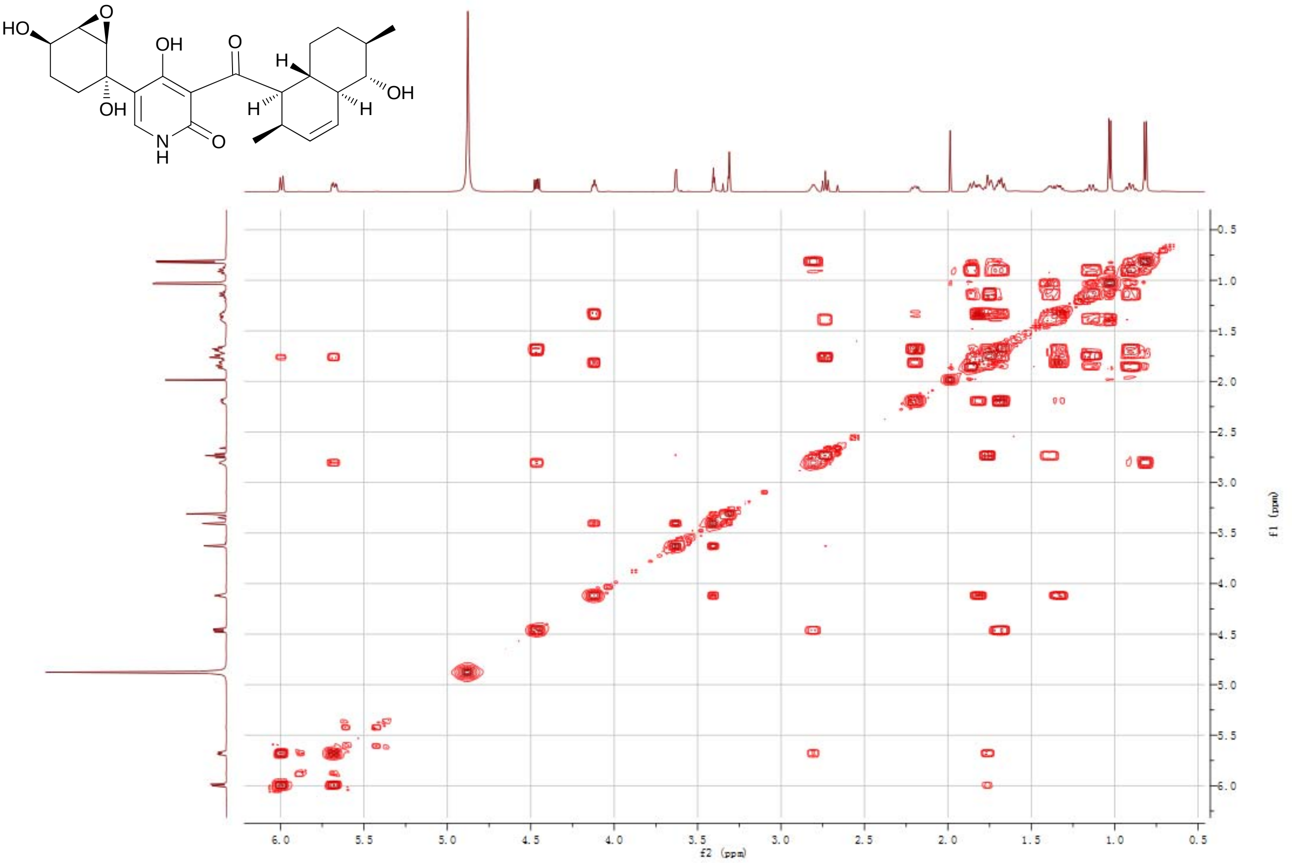

Figure S72. The HMBC spectrum of arthpyrone K **(8)** in CD$_3$OD

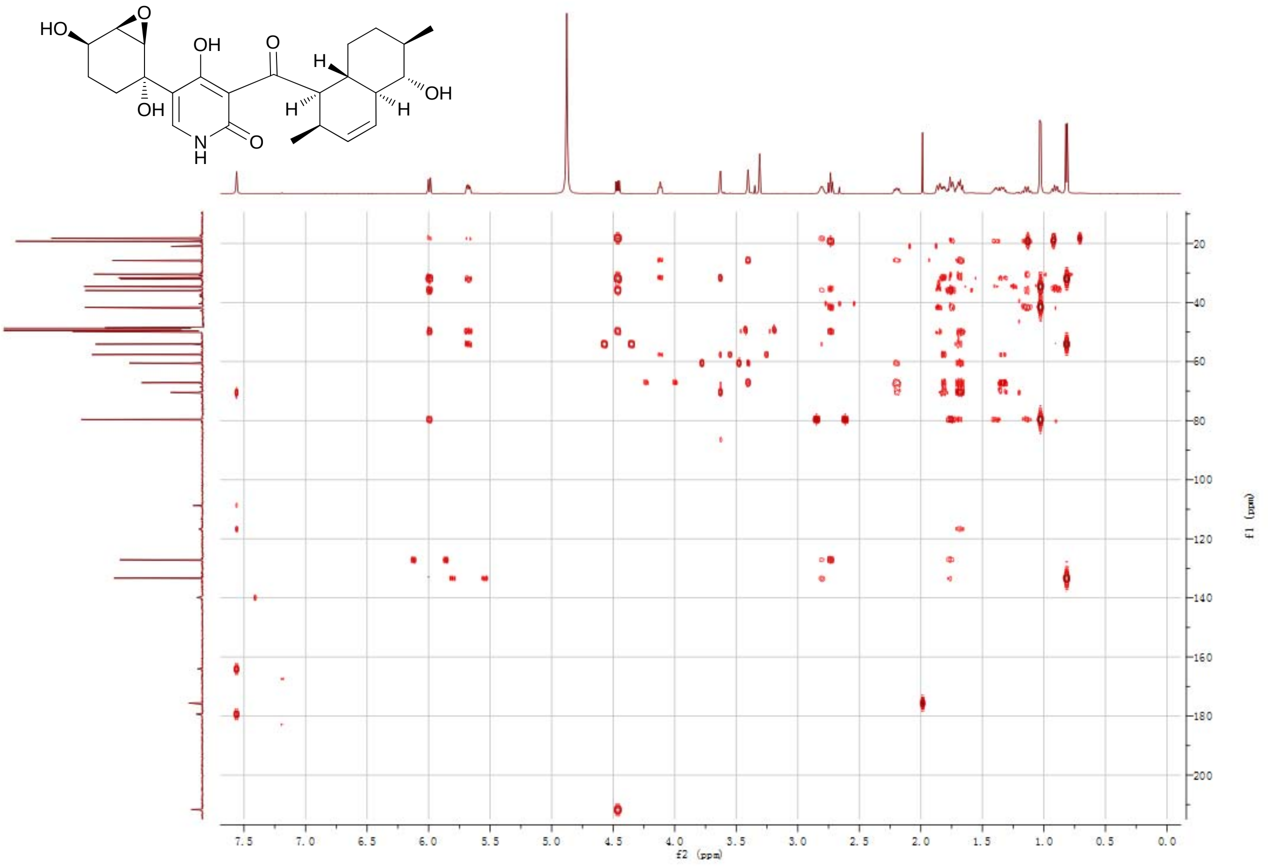

Figure S73. The NOESY spectrum of arthpyrone K **(8)** in CD_3OD

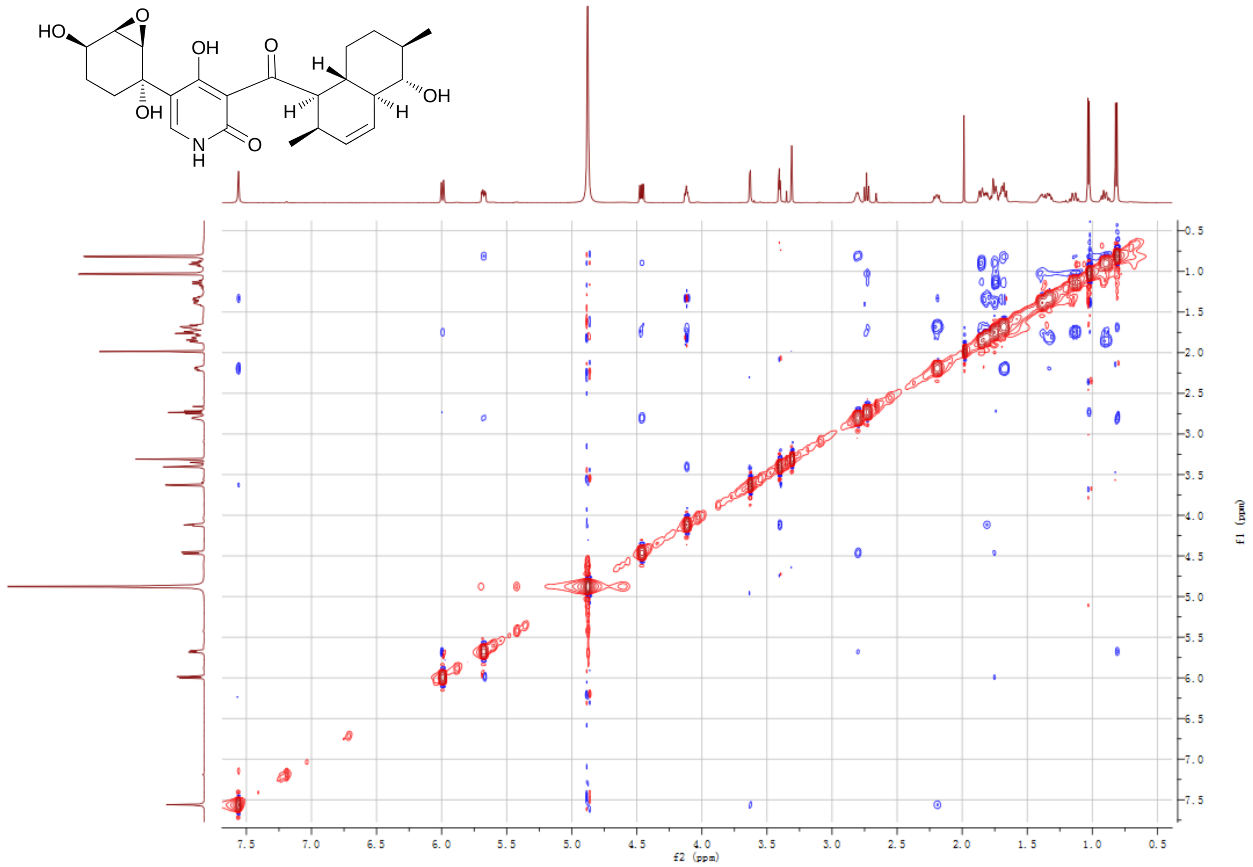

Figure S74. The (-)-HRESIMS spectrum of arthpyrone K **(8)**

**Sample Name**	BJ 53	**Position**	P1-A3	**Instrument Name**	Instrument 1
**User Name**		**Inj Vol**	2	**InjPosition**	
**Sample Type**	Sample	**IRM Calibration Status**	Success	**Data Filename**	BJ 53.d
**ACQ Method**	wss-isocratic elution-Negative.m	**Comment**		**Acquired Time**	11/23/2017 9:12:33 AM

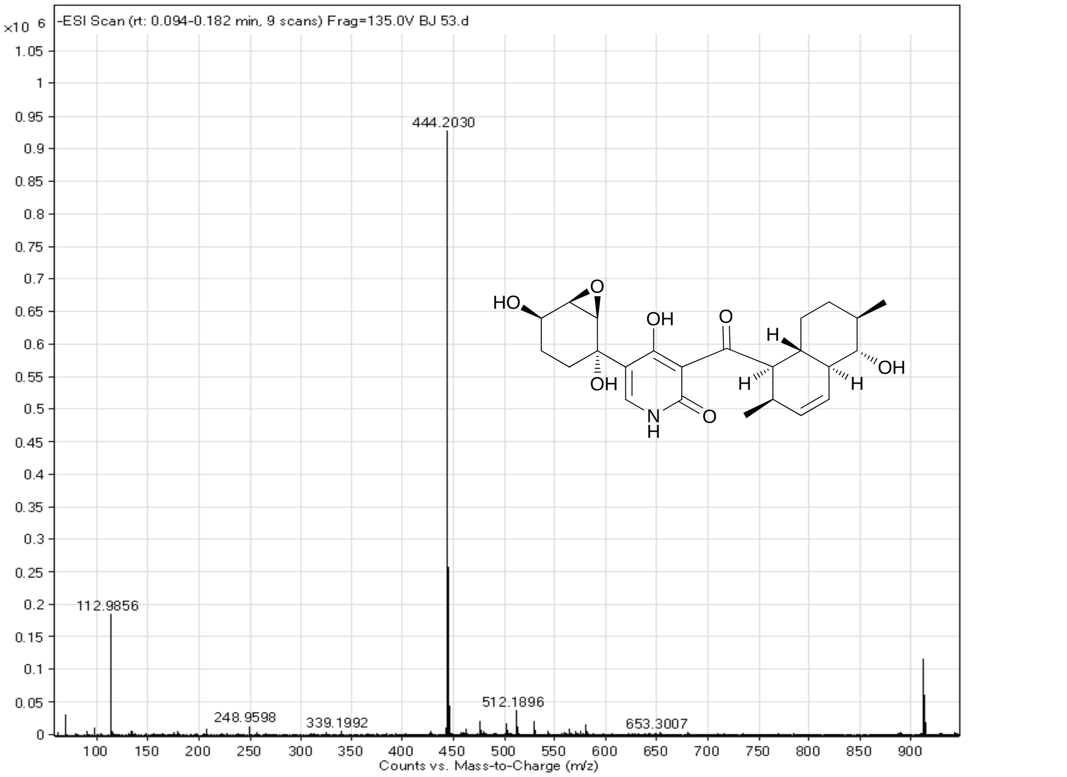

Figure S75. The ^{1}H-NMR spectrum of **10r** and arthpyrone D **(1)** in CD_3OD

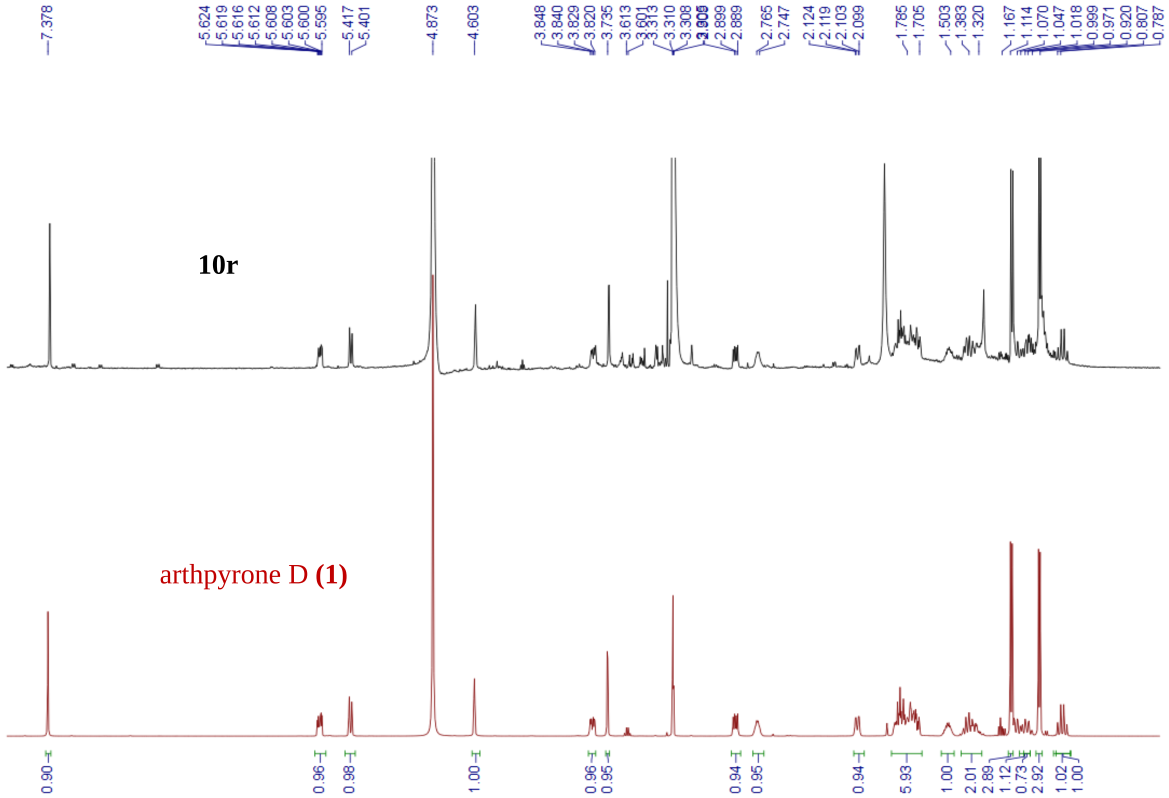

Figure S76. The (-)-ESIMS spectrum of **10r**

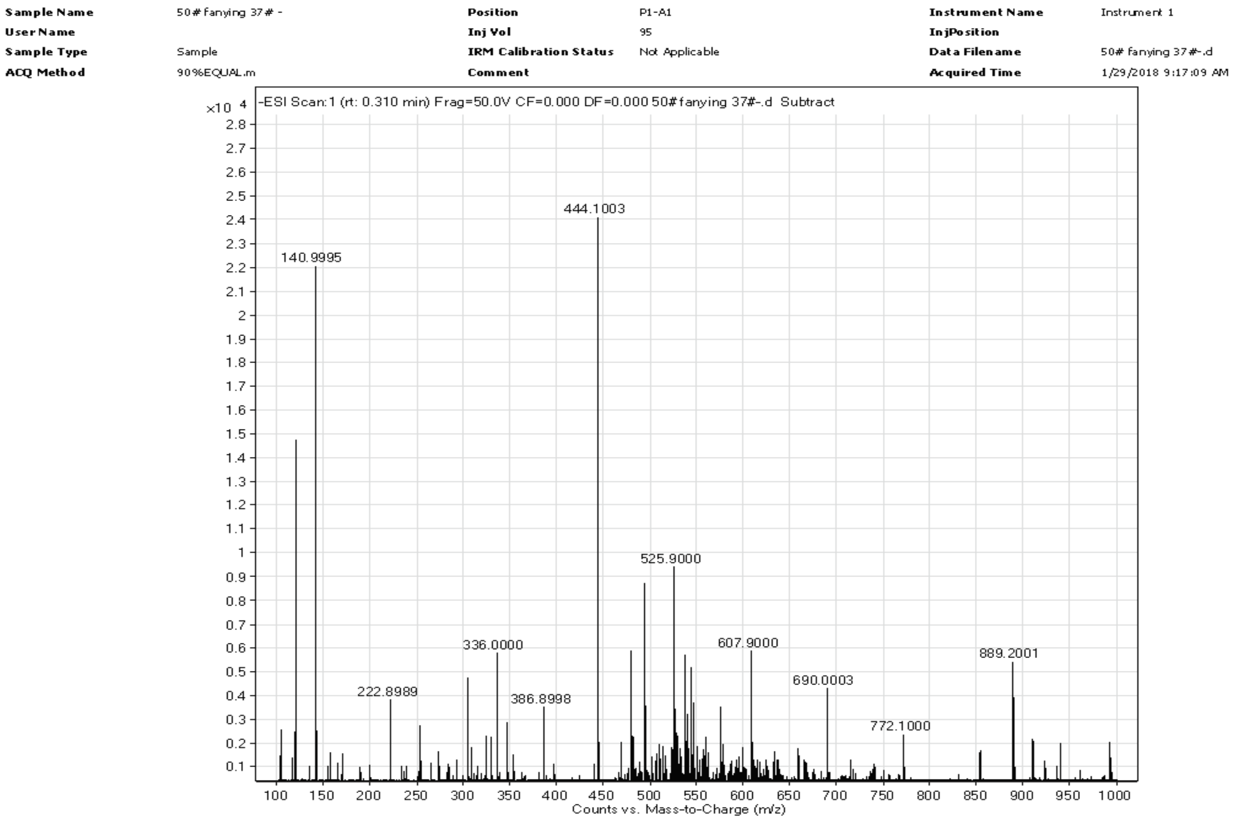

Supplement: Supplementary file 1 [file marinedrugs-16-00174-s001.pdf]
